# Bcl-xL Is a Key Mediator of Apoptosis Following KRAS^G12C^ Inhibition in *KRAS^G12C^*-mutant Colorectal Cancer

**DOI:** 10.1158/1535-7163.MCT-22-0301

**Published:** 2022-10-22

**Authors:** Hajrah Khawaja, Rebecca Briggs, Cheryl H. Latimer, Mustasin Rassel, Daryl Griffin, Lyndsey Hanson, Alberto Bardelli, Frederica Di Nicolantonio, Simon S. McDade, Christopher J. Scott, Shauna Lambe, Manisha Maurya, Andreas U. Lindner, Jochen H.M. Prehn, Jose Sousa, Chris Winnington, Melissa J. LaBonte, Sarah Ross, Sandra Van Schaeybroeck

**Affiliations:** 1 Drug Resistance Group, Patrick G. Johnston Centre for Cancer Research, School of Medicine, Dentistry and Biomedical Science, Queen's University Belfast, Belfast, United Kingdom.; 2 AstraZeneca, Cambridge, United Kingdom.; 3 Department of Oncology, University of Torino, Candiolo, Torino, Italy.; 4 Candiolo Cancer Institute, FPO-IRCCS, Candiolo, Torino, Italy.; 5 Precision Medicine Centre of Excellence, Health Sciences Building, Queen's University Belfast, Belfast, United Kingdom.; 6 Centre of Systems Medicine, Royal College of Surgeons in Ireland University of Medicine and Health Sciences, Dublin 2, Ireland.; 7 Personal Health Data Science Group, Sano. Centre for Computational Personalised Medicine, Krakow, Poland.

## Abstract

Novel covalent inhibitors of KRAS^G12C^ have shown limited response rates in patients with *KRAS*^G12C^-mutant (MT) colorectal cancer. Thus, novel KRAS^G12C^ inhibitor combination strategies that can achieve deep and durable responses are needed. Small-molecule *KRAS*^G12C^ inhibitors AZ’1569 and AZ’8037 were used. To identify novel candidate combination strategies for AZ’1569, we performed RNA sequencing, siRNA, and high-throughput drug screening. Top hits were validated in a panel of *KRAS*^G12C^MT colorectal cancer cells and *in vivo*. AZ’1569-resistant colorectal cancer cells were generated and characterized. We found that response to AZ’1569 was heterogeneous across the *KRAS*^G12C^MT models. AZ’1569 was ineffective at inducing apoptosis when used as a single agent or combined with chemotherapy or agents targeting the EGFR/KRAS/AKT axis. Using a systems biology approach, we identified the antiapoptotic BH3-family member *BCL2L1*/Bcl-xL as a top hit mediating resistance to AZ’1569. Further analyses identified acute increases in the proapoptotic protein BIM following AZ’1569 treatment. ABT-263 (navitoclax), a pharmacologic Bcl-2 family inhibitor that blocks the ability of Bcl-xL to bind and inhibit BIM, led to dramatic and universal apoptosis when combined with AZ’1569. Furthermore, this combination also resulted in dramatically attenuated tumor growth in *KRAS*^G12C^MT xenografts. Finally, AZ’1569-resistant cells showed amplification of KRAS^G12C^, EphA2/c-MET activation, increased proinflammatory chemokine profile and cross-resistance to several targeted agents. Importantly, KRAS amplification and AZ’1569 resistance were reversible upon drug withdrawal, arguing strongly for the use of drug holidays in the case of KRAS amplification. Taken together, combinatorial targeting of Bcl-xL and KRAS^G12C^ is highly effective, suggesting a novel therapeutic strategy for patients with *KRAS*^G12C^MT colorectal cancer.

## Introduction

In colorectal cancer, *KRAS* is the most mutated *RAS* isoform (∼86%), and mutations are most likely to occur in codon 12 (1, 2). KRAS cycles between its inactive, GDP-bound and an active, GTP-bound form that is regulated by either GTP-loading guanine nucleotide-exchange factors or GTPase-activating proteins (GAPs; ref. 3). *KRAS* mutations interfere with the rate of its intrinsic and GAP-induced GTP hydrolysis, favoring formation of the constitutively active GTP-bound form.

Although substantial advances have been made in the treatment of genetically defined subtypes, such as *RAS/BRAF* wild-type (4) and *BRAF*MT colorectal cancer (5), an effective therapeutic strategy for *KRAS*MT colorectal cancer, the most common genetically defined subtype (∼40%–45%) is still lacking. Current treatment options for *KRAS*MT colorectal cancer are primarily based on combinations of chemotherapy with antiangiogenic agents (6). KRAS proteins have long been considered “undruggable” due to their small size and the tight binding of KRAS to GTP in its active state. Recently, unique characteristics of the KRAS^G12C^ allele have been exploited for the design of a number of covalent inhibitors that bind specifically to the cysteine at position 12, thereby locking KRAS in its inactive state (7). *KRAS*^G12C^ can be found in approximately 14% and approximately 4% of lung cancer and colorectal cancer, respectively (8, 9). A recent trial with the KRAS^G12C^ inhibitor AMG510 (sotorasib) has shown remarkable single-agent activity in *KRAS*^G12C^MT lung cancer, but efficacy was not encouraging in *KRAS^G12C^*MT colorectal cancer (10).

Here, we characterize the activity of AZ’1569 (11), a novel KRAS^G12C^ inhibitor, in a panel of *KRAS*^G12C^MT colorectal cancer cells. Using RNA sequencing (RNA-seq), RNAi/compound screens, and mechanistic studies, we identified Bcl-xL as mediator of apoptosis and intrinsic resistance to AZ’1569. We also show that concomitant inhibition of Bcl-xL and KRAS^G12C^ leads to marked increases in therapeutic efficacy in *KRAS*^G12C^MT *in vitro* and xenograft models. Using genomic and proteomic analyses, we show that AZ’1569-acquired resistant models have high-level amplification of the KRAS^G12C^ allele with marked elevation of proinflammatory environment, resulting in resistance to several targeted agents and conventional chemotherapy.

## Materials and Methods

### Materials

AZ’1569 (compound-43), AZ’8037 (compound-25; ref. 11), AZD1480, and AZD6244 (Selumetinib/ARRY-142866) were obtained from AstraZeneca, 5-fluorouracil (5-FU) and oxaliplatin from the Belfast City Hospital Pharmacy, cetuximab from Merck Serono, and crizotinib from Pfizer. Ruloxitinib, capivasertib, ABT-737, and compound library were purchased from Selleckchem, S6K-18 and ABT-263 from Adooq Biosciences, and SN-38 from Abatra. See Supplementary Materials and Methods for references of non–FDA-approved drugs used. siRNA targeting *BCL2L1* and the ON-TARGETplus siRNA library was obtained from Dharmacon. See Supplementary Materials and Methods for details of plasmids.

### Cell culture

C106, SW837, SW1463, SNU1411, LIM2099, and V481 colorectal cancer cells were kindly provided by Prof. Bardelli (12). RW7213 cells were provided by Dr. Arango (University Hospital Vall d'Hebron, Spain; ref. 13). HCT116 cells were purchased as authenticated stocks from ATCC. Frozen stocks were immediately established from early passage cells. Cells were cultured for not more than 20 passages following thawing. All cell lines were screened monthly for *Mycoplasma* (MycoAlert Detection Kit, Lonza). KRAS^G12C^ status was confirmed using Sanger sequencing. See Supplementary Materials and Methods for detailed protocols.

### Generation of AZ’1569-resistant cells

Concentration of AZ’1569 was increased (0.125–1 μmol/L) until a single-cell density was obtained. Surviving RW7213 cells were expanded in the presence of AZ’1569 (maximum concentration 3 μmol/L). Resistance was determined using cell viability assays.

### Protein analysis and Western blotting

Western blotting has been described previously (14, 15). β-actin was used as a loading control. Details of antibodies are provided in Supplementary Table S1. Absolute protein quantification of BAK, BAX, BCL2, Bcl-xL, and MCL-1 was performed as described previously (16).

### Immunoprecipitation

Cells were transfected with 1 μg Myc-tagged Bcl-xL for 24 hours, after which cells were treated with AZ’1569 and/or navitoclax for a further 24 hours. Cells were lysed with SDS-free RIPA supplemented with protease inhibitors, followed by protein quantification, retrieval of inputs, and incubation with Myc-tag antibody conjugated dynabeads overnight. Dynabeads were then washed and boiled in loading buffer for 5 minutes at 95°C before Western blotting analysis.

### Absolute protein quantification for correlation analysis [log (EC_50_) to Bcl-xL/BAK ratio]

To calculate BAK and Bcl-xL protein molar concentrations, densitometry values of basal protein expression were obtained for the panel of *KRAS*^G12C^ MT colorectal cancer cell lines and HCT116 cells. ImageJ software analysis of Western blots for respective proteins, normalized to β-actin (loading control) was used. Fixed molar concentrations were considered for BAK and Bcl-xL in the HCT116 cell line (Bak = 677 nmol/L, Bcl-xL = 604 nmol/L) as determined previously (16). The equation used was protein (nmol/L) value = [(densitometry value (BAK or Bcl-xL) normalized to β-actin)/respective HCT116 value for BAK or Bcl-xL) × respective concentration in HCT116 (nmol/L)]. Correlation analyses were performed using GraphPad Prism 9.0.

### Caspase-3/7 activity assays

Caspase-Glo3/7 activity assays (Promega) have been described previously (15).

### Cell viability assay

Cell viability was determined using 3-(4,5-dimethylthiazol-2-yl)-2,5-diphenyltetrazolium bromide and CellTiter-Glo (CTG) assays (14), according to the manufacturer's instructions. IC_50_ values were calculated using the GraphPad Prism 8 (GraphPad Software, Inc.).

### siRNA and DNA transfections

siRNA and DNA transfections were performed using HiPerfect (Qiagen) and X-tremeGENE (Merck), respectively, described previously (14).

### RNA/DNA extraction and RT-PCR analysis

RNA and DNA extractions were performed using RNeasy and DNeasy Blood and Tissue Kits (Qiagen). A260/280 and 260/230 ratios were used for quality control. RT-PCR was performed as described previously (15). Probes were purchased from Roche and Thermo Fisher Scientific (TFS). See Supplementary Materials and Methods for primer sequences.

### Cytokine/receptor tyrosine kinase arrays and CXCL1/TGF-**α** ELISA

Cytokine, receptor tyrosine kinase (RTK) arrays and ELISAs (R&D systems) were used according to the manufacturer's instructions, as described previously (14). Densitometry was performed using ImageJ.

### RAS-GTP assay

KRAS-GTP expression was evaluated using an active RAS-pulldown kit (TFS) according to the manufacturer's instructions.

### RNA-seq

RNA-seq of AZ’1569-treated SW837 and SNU1411 cells was performed on a NextSeq 500 using a 150-cycle High-Output kit (Illumina) as described previously (14). Additional information is provided in the Supplementary Materials and Methods.

### Sanger sequencing

PCR products were cleaned up using Agencourt AM pure beads on the Hamilton Microlab STAR liquid handling robot and Sanger sequencing was performed. Electrophoresis of sequencing products was performed on the ABI-3730 48-capillary DNA analyzer. Chromatograms were visualized using Geneious software. See Supplementary Materials and Methods for primer sequences.

### MedExome sequencing and next-generation sequencing

MedExome and next-generation sequencing (NGS) of RW7213 parental and resistant cells was performed using the Illumina Novaseq 6000 and NextSeq 500 (Illumina), respectively. Additional information is provided in the Supplementary Materials and Methods.

### *In vitro* migration assays

Migration assays have been described previously (17). Additional information is provided in the Supplementary Materials and Methods.

### *In vivo* study

*In vivo* studies were conducted as previously described using 6- to 8-week-old, female NOD SCID mice (Envigo; ref. 14). Details of the initial xenograft growth curves and tolerability studies are provided in the Supplementary Materials and Methods (Supplementary Fig. S5A and S5B). For the efficacy study, 2.5 × 10^6^ SNU1411 or 10 × 10^6^ SW1463 cells were injected into the flank of NOD SCID mice. Mice received vehicle (10% ethanol, 30% polyethylene glycol 400, and 60% Phosal 50 PG orally), AZ’8037 (100 mg/kg orally), navitoclax (100 mg/kg orally), or AZ’8037 (100 mg/kg) with navitoclax (100 mg/kg). Each treatment group contained eight animals. AZ’8037 was administered daily and navitoclax was administered 5 out of 6 days. Experiments were carried out according to UKCCCR guidelines under license PPL2875, in accordance with the Animals (Scientific Procedures) Act 1986, approved by the Department of Health, Social Services and Public Safety, Northern Ireland and the Animal Welfare Ethical Review Body.

### Statistical analysis

Robust z-scores (rZ = median/median absolute deviation) were calculated from cell viability assays. All data were plotted (mean and SD, unless specified otherwise) and analyzed using GraphPad Prism 8.0. Significance was defined as *, *P* < 0.05; **, *P* < 0.01; ***, *P* < 0.001, with *P* > 0.05 not significant (ns). Experiments are representative of three independent repeats unless indicated otherwise. The nature of interaction between AZ’1569 and a second drug was determined by calculating combination index (CI) values according to the Chou-Talalay method (18), using CalcuSyn (Microsoft Windows). CI values <1, >1, and = 1 indicate synergy, antagonism, and additive effects, respectively. R index (RI) values were used when one compound had minimal and/or no effect on cell viability. RI values >1 indicate synergy (19).

### Data availability

Raw data for RNA-seq and MedExome sequencing experiments have been deposited at the relevant NCBI platforms, under the accession numbers GSE198530 and PRJNA815942, respectively.

## Results

### *KRAS* ^G12C^MT colorectal cancer cells show differential sensitivity to the KRAS^G12C^ inhibitor AZ’1569

**Figure 1.**
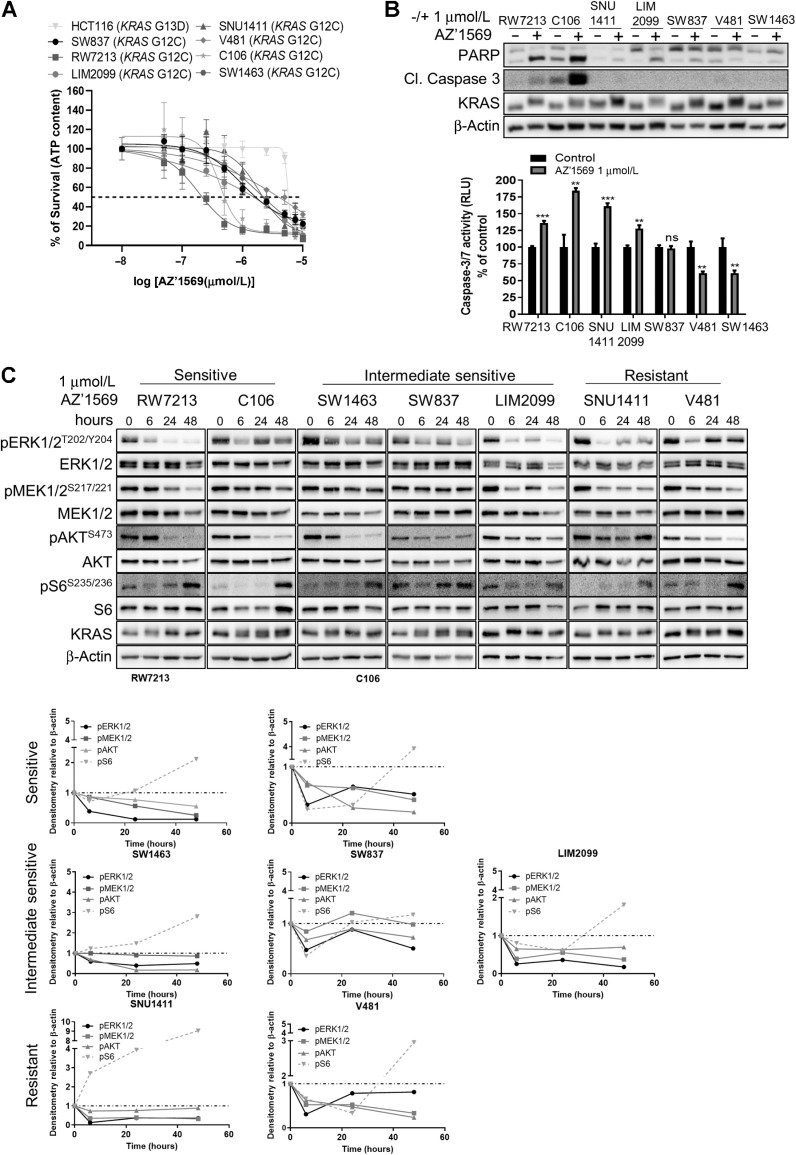
Response to AZ’1569 in *KRAS*^G12C^ MT colorectal cancer cells. **A,***KRAS*^G12C^MT colorectal cancer cells were treated with increasing concentrations of AZ’1569 for 120 hours and cell viability determined using CTG assay. IC_50_ values were calculated using Prism software package. Dashed line indicates 50% cell viability. Representative of three independent experiments is shown. **B,** Colorectal cancer cells were treated with AZ’1569 for 48 hours. PARP, cleaved C3, and KRAS were determined by WB (top), caspase-3/7 activity levels were measured with values presented as a percentage of their respective controls. Significance was analyzed using an unpaired *t* test (bottom). (Cl = cleaved). **C,** Signaling analysis upon AZ’1569 treatment. *KRAS*^G12C^ MT colorectal cancer cell lines were treated with 1 μmol/L AZ’1569 for the indicated times, and protein lysates were used for WB analysis for the KRAS downstream effectors. Densitometry on WB images was quantified using ImageJ software and normalized to the respective untreated control. Dashed lines on the graphs represent a value of 1.

To understand the mechanistic basis for the minor clinical responses to KRAS^G12C^ inhibition in colorectal cancer (10), we analyzed the effect of AZ’1569 in a panel of 7 *KRAS*^G12C^MT colorectal cancer cells. Initially, we validated the KRAS mutational status using Sanger sequencing of exon 2, confirming that four cell lines had a homozygous *KRAS*^G12C^ mutation and three had a heterozygous *KRAS*^G12C^ mutation (Fig. 1A). Five cell lines were found to have a *TP53* mutation, and the V481 cells, were *PIK3CA*MT (Q546P) with loss of PTEN, confirming the results of previous studies (refs. 20–23; https://web.expasy.org/cellosaurus/). Thus, the genetic background of these models captures some of the heterogeneity of *KRAS*MT colorectal cancer observed in tumors (24).

Response to AZ’1569 was measured using a cell viability assay (Fig. 1A; Supplementary Fig. S1A). RW7213 and C106 cells showed the highest sensitivity to AZ’1569 with IC_50_ values of 0.26 and 0.43 μmol/L, compared with IC_50_ values of 1.39, 1.54, 1.72 μmol/L for SW1463, SW837, LIM2099 cells (“moderately sensitive”) and 2.96 and 3.4 μmol/L for SNU1411 and V481 cells (“resistant”). Only RW7213 and C106 cells showed marked induction of apoptosis 24 hours after AZ’1569 treatment, as indicated by PARP cleavage and caspase-3/7 activity (Fig. 1B). Of note, AZ’1569 single-agent activity was not predicted by the G12C zygosity status, p53 mutational status, or by KRAS, EGFR, ERK1/2^T202/Y204^, AKT^S473^ or S6^S235/6^ expression levels (Supplementary Fig. S1A). As expected, *KRAS*^G13D^MT HCT116 cells were resistant to AZ’1569.

Next, we explored differences in depth, duration, and feedback signaling in response to AZ’1569 treatment between our colorectal cancer models with low, intermediate, and high AZ’1569 IC_50_ values. Colorectal cancer cells were treated with AZ’1569 in a time course (Fig. 1C). A similar KRAS electromobility shift, indicative of covalent compound binding, was observed following AZ’1569 treatment across the colorectal cancer panel, suggesting that differential sensitivity was unlikely due to differences in target engagement (25). KRAS^G12C^ inhibition resulted in profound downregulation in pERK1/2 levels as early as 6 hours after treatment in all cell lines. Further decreases in pERK1/2 levels were observed in RW7213 and LIM2099 cells 24 to 48 hours after treatment, while all the other cell lines showed a rebound in pERK1/2 levels. A total of 24 hours posttreatment, AZ’1569 caused sustained suppression of pAKT levels in the three most sensitive cell lines, although minor pAKT decreases were also observed in the most resistant cell line, V481. S6 phosphorylation was transiently reduced in the most sensitive cell lines, but marked reactivation was observed in all the cell lines. Altogether, a range of effects of AZ’1569 on downstream signaling dynamics was observed, and these were not sufficient to explain the differences in viability and/or apoptosis outcome following AZ’1569 treatment.

### KRAS^G12C^ inhibition does not sensitize *KRAS*^G12C^MT colorectal cancer cells to chemotherapy

**Figure 2.**
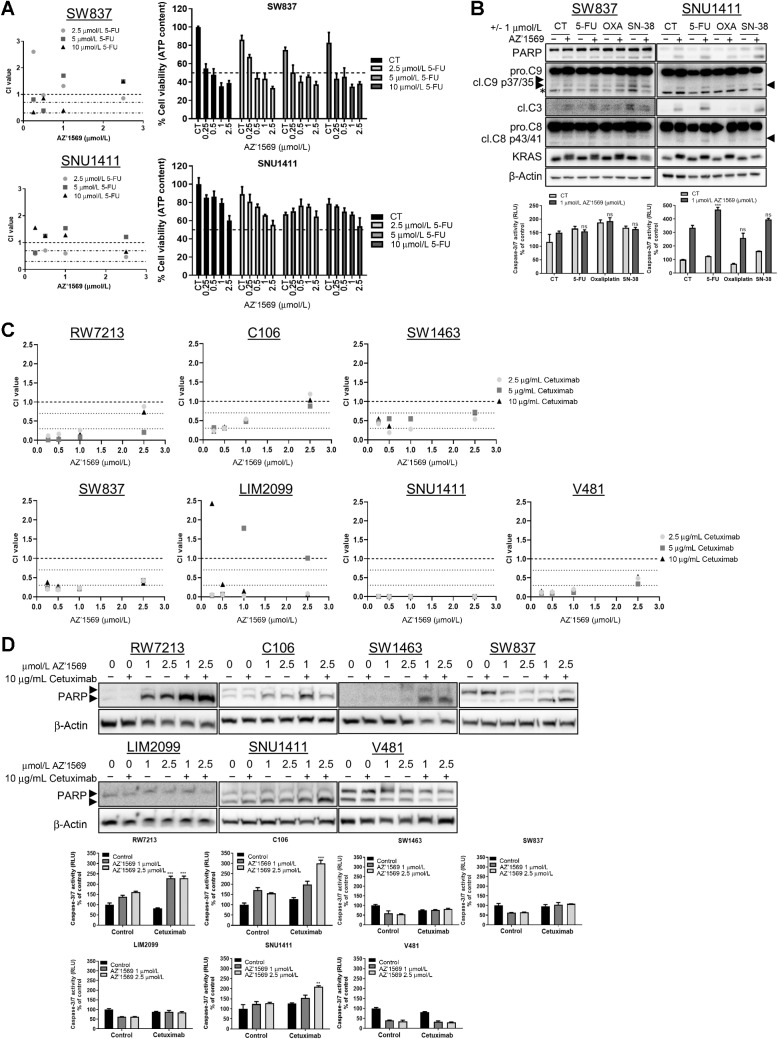
AZ’1569 combined with chemotherapy or cetuximab does not induce apoptosis in *KRAS*^G12C^ MT colorectal cancer cells. **A,** CTG assays in colorectal cancer cells treated with no drug (control), 5-FU, AZ’1569, or 5-FU in combination with AZ’1569 for 72 hours. CI values were calculated using the method of Chou and Talalay. CI values <1, >1, and equal to 1 indicate synergy, antagonism, and additive effects for the drug combinations, respectively. Dashed lines indicate CI values of 0.3, 0.7, and 1. Representative results of at least three experiments (left). Absolute cell viability for different combinations (right). Dashed line indicates 50% cell viability. **B,** Colorectal cancer cells were cotreated with AZ’1569 and 5-FU, oxaliplatin, or SN-38 for 48 hours. Top: PARP, caspase 9, caspase 3, caspase 8, and KRAS levels were determined by WB. Bottom: Apoptosis was assessed by caspase 3/7 activity assay. * = nonspecific band. **C,** CTG cell viability assays in *KRAS*^G12C^ MT colorectal cancer cells cotreated with AZ’1569 and cetuximab for 120 hours. CI values were calculated to evaluate the nature of interaction. **D,***KRAS*^G12C^ MT colorectal cancer cells were cotreated with AZ’1569 and cetuximab for 48 hours, apoptosis was assessed by WB analysis for PARP (top) and caspase-3/7 activity assays (bottom). A two-way ANOVA was used to analyze statistical significance.

5-FU–based doublet therapies (5-FU+oxaliplatin; 5-FU+irinotecan) remain the cornerstone of treatment for patients with *KRAS*MT colorectal cancer (6). We therefore evaluated whether AZ’1569/5-FU, AZ’1569/oxaliplatin, and AZ’1569/SN-38 combined treatments could effectively suppress the growth of *KRAS*^G12C^MT colorectal cancer cells and used the Chou-Talalay method to calculate CI values (18). CI values for combined AZ’1569/5-FU treatment were >0.7 for the majority of the concentrations, indicative of additive interactions (Fig. 2A). Moreover, the absolute cell viability remained > 40% for the majority of combinations. Similar results were obtained for AZ’1569/oxaliplatin and AZ’1569/SN-38 combinations (Supplementary Fig. S1B). In addition, except for combined AZ’1569/5-FU treatment in the SNU1411 cells, none of the AZ’1569/chemotherapy combinations resulted in further increases in apoptosis compared with the effect of each treatment alone (Fig. 2B).

We next sought to identify pharmacologic combinations that could overcome primary resistance to KRAS^G12C^ inhibition. On the basis of the known roles for MAPK, AKT, STAT3, and EGFR signaling in intrinsic and/or acquired resistance to targeted therapies and their relevance in colorectal cancer, we next evaluated whether combining AZ’1569 with either MEK1/2 inhibitor AZD6244, AKT inhibitor capivasertib, JAK/STAT inhibitors ruloxitinib/AZD1480 or EGFR inhibitor cetuximab could effectively suppress colorectal cancer cell viability (Fig. 2C and D; Supplementary Figs. S1C–S1H). Notably, only concurrent cetuximab/AZ’1569 treatment showed moderate and/or strong synergy across all *KRAS*^G12C^MT colorectal cancer cells tested (Fig. 2C). Although combined cetuximab/AZ’1569 treatment resulted in major reduction in cell viability (Supplementary Fig. S1G), efficient apoptosis induction occurred only in RW7213 and C106 cells (Fig. 2D).

### *BCL2L1* is a key regulator of apoptotic response to KRAS^G12C^ inhibition in *KRAS*^G12C^MT colorectal cancer cells

Cytostatic and cytotoxic drugs have been linked to clinically observed disease stabilization and objective responses, respectively (26). To gain further insight into the molecular mechanisms of apoptosis following AZ’1569 treatment, we performed RNA-seq analysis before the onset of cell death in SW837 and SNU1411 cells (Supplementary Fig. S2A and S2B; GSE198530). Significant downregulation of the MAPK pathway negative feedback mediators *DUSP4/6* and *SPRY4* was observed 6 hours post-AZ’1569 treatment in both SW837 and SNU1411 cells, confirming inhibition of ERK1/2 activity (Supplementary Fig. S2C). To identify pathways that are involved in resistance to KRAS^G12C^ inhibition, Ingenuity Pathway Analysis (IPA) was conducted using the three gene lists generated for both cell lines. IPA comparison analyses of upregulated and downregulated pathways showed that 63 pathways overlapped and were significantly deregulated across all the timepoints analyzed in both cell lines, with a significant enrichment of gene sets in cell death–related signaling pathways, including death receptor, apoptosis, necroptosis, and *TP53* signaling (Supplementary Fig. S2D and S2E).

**Figure 3.**
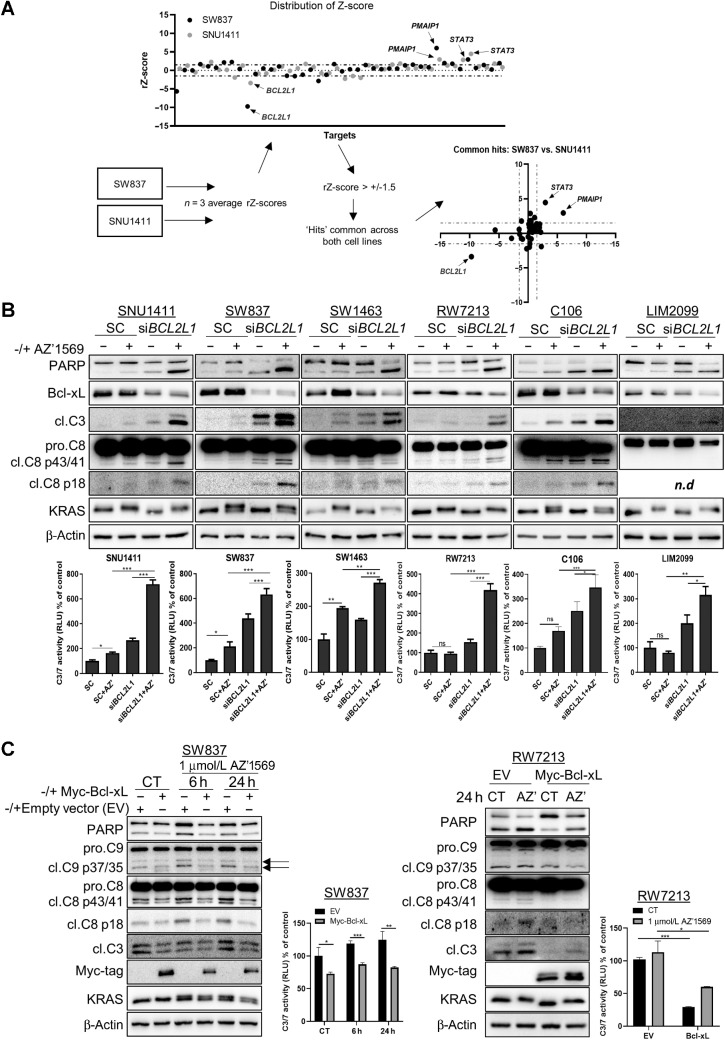
Bcl-xL regulates intrinsic resistance to KRAS^G12C^ inhibition in *KRAS*^G12C^ MT colorectal cancer**. A,** Targeted siRNA screen in SW837 and SNU1411 cells. Top: SW837 and SNU1411 cells were reverse transfected with 10 nmol/L ON-Targetplus siRNA's targeting 42 genes in the absence or presence of 1 μmol/L AZ’1569 for 72 hours and cell viability was evaluated using the CTG assay. Scatter plot showing rZ for siRNA screen in SW837 and SNU1411 cells. Positive scores indicate potential mediators of sensitivity to AZ’1569, while negative scores indicate mediators of resistance to AZ’1569. Dashed lines indicate rZ = 0, 1.5, and −1.5; cut-off thresholds of ±1.5 were applied to the data. Bottom: The siRNA approach and analysis. XY graph illustrates hits resulting in sensitization or resistance to AZ’1569 in both cell lines. Data show average rZ-scores from three independent experiments. **B,** Colorectal cancer cells were transfected with 10 nmol/L on-target SMARTpool siRNA against *BCL2L1* and cotreated with 1 μmol/L AZ’1569 (0.25 μmol/L AZ’1569 for RW7213 and C106 cells) for 24 hours (48 hours for SNU1411) and apoptosis assessed by WB for PARP and cleaved caspase 8 and 3 (top) and caspase 3/7 activity assay (bottom). n.d denotes not detected. A two-way ANOVA was used to evaluate significance. **C,** Expression of PARP, cleaved caspase 9, caspase 8, Myc-tag, and KRAS in SW837 and RW7213 cells transiently transfected with 1 μg of Myc-tagged Bcl-xL for 24 hours, followed by treatment with 1 μmol/L AZ’1569 (AZD) for the indicated times. Caspase-3/7 activity on cell lysates was also determined. A two-way ANOVA was used to evaluate significance.

To identify key functional genes and/or targets that, when inhibited, cooperate with KRAS^G12C^ inhibition to decrease survival, and increase apoptosis in *KRAS*^G12C^MT colorectal cancer cells, we used an RNAi screening approach targeting proteins that lie at nodal points in the identified cell death–related signaling pathways. The effect of downregulating each of these proteins on cell viability was tested in both SW837 and SNU1411 cells, using an ON-TARGETplus siRNA library against 42 targets (Supplementary Fig. S3A) in the absence and presence of AZ’1569 treatment and rZ values were calculated. Notably, only one of 42 siRNAs had a significant inhibitory effect on survival in the presence of AZ’1569 in both cell lines, and this was *BCL2L1*, the gene encoding the antiapoptotic BH3-family member Bcl-xL (Fig. 3A). To exclude cell line–specific effects, we extended these studies to a broader panel of *KRAS^G12C^*MT colorectal cancer cells and also confirmed the cytotoxic activity of the combination by using apoptotic cell death assays, described previously (Fig. 3B). *BCL2L1* silencing resulted in marked increases in apoptosis when combined with AZ’1569 in all *KRAS*^G12C^MT colorectal cancer models, compared with the effects of each treatment alone. In addition, transient overexpression of Myc-tagged Bcl-xL led to marked reduction in basal and AZ’1569-induced apoptosis in SW837 cells (Fig. 3C). Similar effects were observed in the RW7213 cells.

DR_MOMP was previously developed to predict the stress dose required in a cell to induce mitochondrial outer membrane permeabilization (MOMP), as a readout of sensitivity of colorectal cancer cells to genotoxic chemotherapy (16, 27). To evaluate whether sensitivity to AZ’1569 correlated with expression levels of proapoptotic and antiapoptotic BCL-2 family members or DR_MOMP stress dose, we initially determined the basal absolute BCL-2 proteins profiles (BAK, BAX, BCL-2, Bcl-xL, MCL1) in our *KRAS*^G12C^MT colorectal cancer cells (Supplementary Fig. S3B). Not surprisingly, given the heterogeneity of *KRAS*MT colorectal cancer, levels of BCL-2 proteins were variable across the cell line panel. Interestingly, Bcl-xL/BAK ratio correlated with response to AZ’1569 (Supplementary Fig. S3B, *r* = 0.54), indicating that cells with an increased Bcl-xL/BAK ratio show an unfavorable response to AZ’1569 treatment. There was no correlation between DR_MOMP calculated stress dose and sensitivity to AZ’1569 treatment. Next, we assessed basal and AZ’1569-induced levels of the proapoptotic and antiapoptotic BCL-2 family proteins, including the MOMP effector proteins BAX and BAK (Supplementary Fig. S3C). Notably, BIM levels were markedly higher in the AZ’1569-sensitive RW7213 and C106 cells, compared with the levels observed in the intermediate sensitive and resistant cell lines. Expression of BIM was also acutely increased following AZ’1569 treatment in all *KRAS*^G12C^MT colorectal cancer cell lines—in particular, the RW7213 and C106 cells. Immunoprecipitation of BIM confirmed that BIM levels were induced by AZ’1569, and the BH3-mimetic ABT-263 (navitoclax) completely disrupted the association of Bcl-xL with BIM under basal conditions and following BIM induction by AZ’1569 (Supplementary Fig. S3D). Collectively, these data indicate that concomitant suppression of the antiapoptotic protein Bcl-xL, thereby “freeing” BIM, is needed for a robust apoptotic response after KRAS^G12C^ inhibition in *KRAS*^G12C^MT colorectal cancer.

### The BH3-mimetic ABT-737 potently synergizes with KRAS^G12C^ inhibition

**Figure 4.**
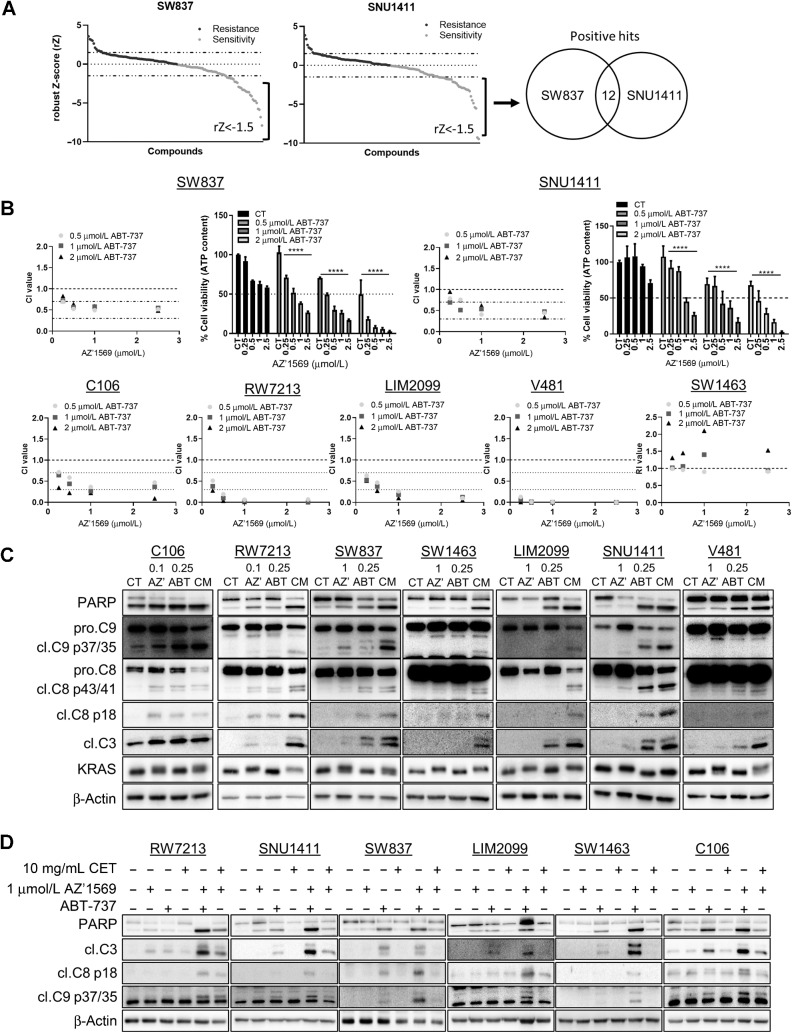
High-throughput drug screen reveals that pharmacologic inhibition of Bcl-xL synergizes with KRAS^G12C^ inhibition in *KRAS*^G12C^ MT colorectal cancer. **A,** SW837 and SNU1411 cells were cotreated with 1 μmol/L AZ’1569 alone or combined with a panel of 45 small-molecule inhibitors for 72 hours and cell viability assessed using the CTG assay. Three concentrations per drug were tested (Supplementary Table S2). Cell viability was analyzed using a CTG assay. Scatter plot showing rZ for each compound concentration used in the drug screen. Negative rZ-scores indicate agents that sensitize to AZ’1569, and *vice versa*. Dashed lines on graphs indicate values of 1.5 and −1.5. Venn diagram indicates number of compounds (past a threshold of rZ = −1.5) that resulted in sensitization to AZ’1569 in both cell lines. **B,** CTG cell viability assays in *KRAS*^G12C^ MT colorectal cancer cells cotreated with AZ’1569 and ABT-737 for 72 hours. CI values were calculated to evaluate the nature of interaction. Absolute cell viability for AZ’1569/ABT-737 combinations in SW847 and SNU1411 cell lines are also shown. Dashed lines on graphs represent 50% cell viability. **C,** PARP, cleaved C9, cleaved C8, cleaved C3, and KRAS expression levels in *KRAS*^G12C^ MT colorectal cancer cells cotreated with AZ’1569 and ABT-737 for 48 hours (24 hours for RW7213, SW1463, and V481 cells). CM = combination. **D,***KRAS*^G12C^ MT colorectal cancer cells were treated with AZ’1569 alone or combined with cetuximab or ABT-737 (0.25 μmol/L for C106, LIM2099, SNU1411; 0.5 μmol/L for SW837, V481, RW7213 and 2.5 μmol/L for SW1463) for 48 hours (24 hours for C106 cells) and PARP, cleaved C9, cleaved C8, and cleaved C3 determined by WB.

To complement the siRNA profiling results, we performed a focused drug screen to identify compounds that could effectively suppress viability of *KRAS*^G12C^MT colorectal cancer cells when combined with AZ’1569. We used a drug library targeting the top druggable pathways previously identified (Supplementary Fig. S2D). On the basis of potential for clinical application, we prioritized 45 compounds (Supplementary Fig. S4A), including activators of intrinsic and/or extrinsic cell death and cell-cycle regulators. The effect of these drugs in the absence and presence of AZ’1569 was tested in SW837 and SNU1411 cells. Positive hits were identified as compounds that resulted in rZ less than −1.5 in three independent experiments in both cell lines; this identified 12 hits (Fig. 4A). To further refine our hit list, we determined synergy between these 12 compounds and AZ’1569, using the Chou-Talalay method in SW837 and SNU1411 cells. ABT-737 and Entinostat were the most synergistic with AZ’1569 in both cell lines (Fig. 4B; Supplementary Fig. S4B and S4C), with ABT-737 resulting in the most potent growth suppression when combined with AZ’1569 in the extended panel of *KRAS*^G12C^MT colorectal cancer cells (Fig. 4B; Supplementary Fig. S4D and S4E). Combined ABT-737/AZ’1569 treatment resulted also in potent increases in apoptosis as indicated by increased PARP cleavage and caspase-9/8/3 processing in all *KRAS^G12C^*MT colorectal cancer cells (Fig. 4C; Supplementary Fig. S4F). Notably, combined ABT-737/AZ’1569 treatment resulted in higher levels of apoptosis, compared with the levels observed with cetuximab/AZ’1569 (Fig. 4D; Supplementary Fig. S4G), suggesting that the ABT-737/AZ’1569 combined strategy could have a more beneficial effect on tumor shrinkage and objective responses in a clinical setting (26).

### *KRAS* ^G12C^MT colorectal cancer xenograft models are sensitive to combinatorial Bcl-xL/KRAS^G12C^ inhibition

**Figure 5.**
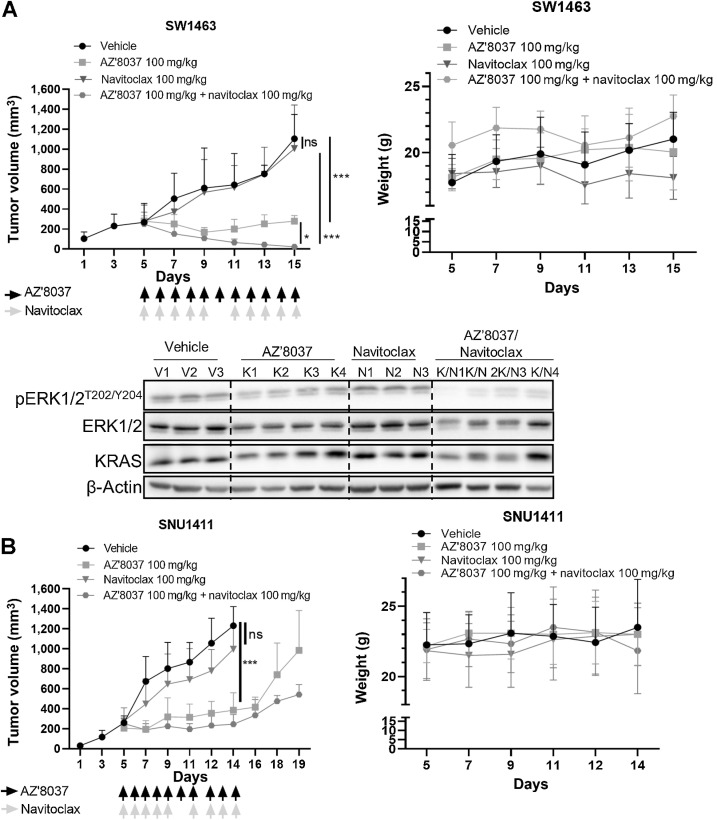
Combined KRAS^G12C^ and Bcl-xL inhibition results in reduction in growth of *KRAS*^G12C^ MT colorectal cancer *in vivo*. **A,** Growth rate (left) and mouse weight (right) of SW1463 xenografts in NOD/SCID mice treated with vehicle, AZ’8037, navitoclax, or AZ’8037 in combination with navitoclax. Differences in growth were determined using a one-way ANOVA with Tukey test for multiple comparisons. WB analysis for pERK1/2, ERK1/2, and KRAS in tumor samples collected at day 15. **B,** Growth rate (left) and mouse weight (right) of SNU1411 xenografts in NOD/SCID mice treated with vehicle, AZ’8037, navitoclax, or AZ’8037 in combination with navitoclax.

We next assessed the *in vivo* therapeutic efficacy of combined Bcl-xL/KRAS^G12C^ inhibition. We selected two different *KRAS*^G12C^MT colorectal cancer models, SW1463 and SNU1411 that showed exponential growth characteristics when grown as xenografts (Supplementary Fig. S5A) and used the orally bioavailable BH3-mimetic navitoclax and the orally bioavailable KRAS^G12C^ inhibitor and close analog of AZ’1569, AZ’8037. The SW1463 model was resistant to single-agent navitoclax treatment and exhibited slowed but persistent growth when mice were treated with AZ’8037 (Fig. 5A). Combination treatment of navitoclax/AZ’8037 resulted in marked tumor shrinkage in the treated animals. Strong pERK1/2 inhibition was observed, in particular, in the Navitoclax/AZ’8037 cotreated tumor samples. Similar to our results in the SW1463 model, single-agent AZ’8037 slowed SNU1411 tumor growth (Fig. 5B). There was also no effect of single-agent navitoclax. Although addition of navitoclax to AZ’8037 resulted in further reduction in tumor growth, there was no tumor regression in the SNU1411 xenografts. Treatment cessation resulted in tumor regrowth in AZ’8037 monotherapy and navitoclax/AZ’8037 combination groups (Fig. 5B). The navitoclax/AZ’8037 combination was less well tolerated in this second mouse model as shown by decreases in tumor weight in week 2 of the treatment (Fig. 5B). Navitoclax was therefore given as a 5-day-on, 1-day-off schedule. Collectively, these results indicate that Bcl-xL–targeted agents may be highly effective when used in combination with KRAS^G12C^ inhibitors in *KRAS*^G12C^MT colorectal cancer.

### Generation of colorectal cancer models with acquired resistance to KRAS^G12C^ inhibition

**Figure 6.**
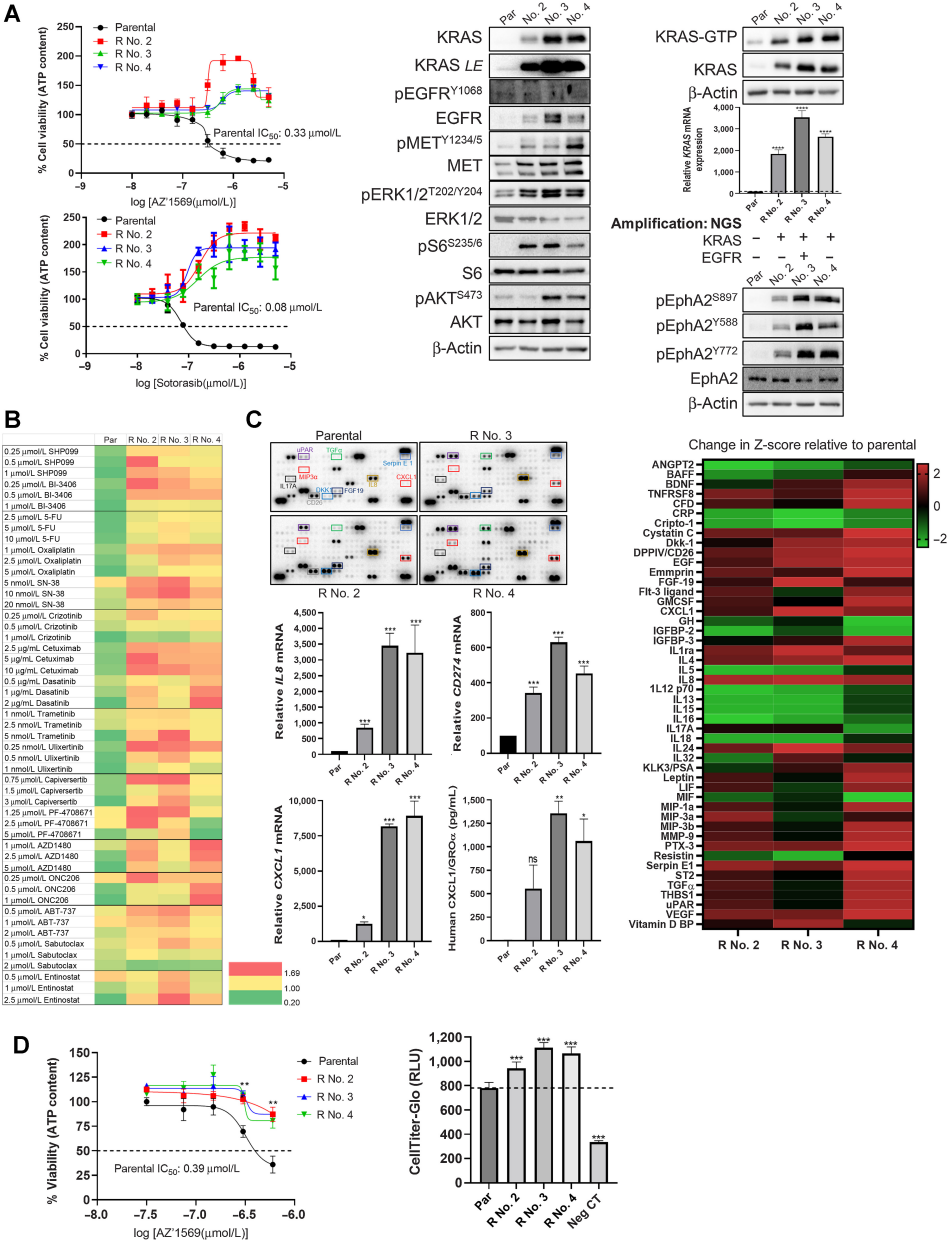
AZ’1569-acquired resistant cells exhibit increased PD-L1 expression and a proinflammatory phenotype. **A,** Left: RW7213 parental and AZ’1569-resistant clones (No. 2, No. 3, and No. 4) were treated for 72 hours with indicated concentrations of AZ’1569 or sotorasib, and cell viability was determined using CTG assays. Right: Lysates from RW7213 parental and AZ’1569-resistant clones were analyzed by WB for KRAS, pEGFR^Y1068^, EGFR, pMET^Y1234/1235^, MET, pERK1/2^T202/Y204^, ERK1/2, pS6^S235/6^, S6, pAKT^S473^, AKT, pEphA2^S897^, pEphA2^Y588^, pEphA2^Y772^, and EphA2. Active Raf1-bound Ras was isolated from RW7213 parental and resistant clones using an RAS-GTP assay and basal GTP-bound and total KRAS levels assessed by WB. *LE* = longer exposure. *KRAS* mRNA was quantified using RT-PCR. Raw values were normalized to *ACTB* and *GAPDH* expression and were analyzed using the ΔΔ*C*_T_ method. A one-way ANOVA was used to calculate statistical significance. Data are representative of three independent experimental repeats. Results of NGS of RW7213 Par and AZ’1569-R clones are shown. **B,** RW7213 parental and resistant cells were treated with SHP-099, BI-3406, 5-FU, SN-38, oxaliplatin, crizotinib, cetuximab, dasatinib, trametinib, ulixertinib, capivasertib, PF-4708671, AZD1480, ONC206, ABT-737, sabutoclax or entinostat for 72 hours, at the indicated concentrations and cell viability was assessed using CTG assays. Heatmap represents cell viability relative to control. Data are representative of three independent experimental repeats. **C,** Top left: Human cytokine array using conditioned medium of RW7213 parental and resistant clones. Right: Mean spot pixel density was analyzed using Image J, rZ (relative to parental cells) were calculated using densitometry data and presented in a heatmap. Bottom left: *CXCL1*, *CD274*, and *IL8* mRNA in parental and resistant clones were quantified using RT-PCR. Raw values were normalized to the expression of housekeeping genes *ACTB* and *GAPDH* and were analyzed using the ΔΔ*C*_T_ method. CXCL1 protein levels in the culture media of parental (Par) and resistant subpopulations were measured by ELISA. A one-way ANOVA was used to calculate statistical significance. Data are representative of three independent experimental repeats. **D,** Left: Dose–response curves for AZ’1569 in RW7213 cells, incubated with conditioned media from parental cells or drug-resistant clones No. 2, No. 3, or No. 4. Cells were treated for 72 hours and cell viability was determined using CTG assay. IC_50_ values were calculated using a Prism software package. Dashed line indicates 50% cell viability. A representative of three independent experiments is shown. Right: A 24-well 5-μm polycarbonate Transwell insert-plate system was used. 2.5 × 10^5^ PBMCs were resuspended in 2% FCS-supplemented DMEM and were added to the top chamber. The bottom chamber was filled with conditioned medium (medium = 2% FCS-supplemented DMEM) obtained from RW7213 parental and resistant cells. Cells were incubated for 4 hours, following which CellTiter-Glo was used to measure PBMC migration to the bottom chamber (RLU = relative luminescence). Serum-free DMEM was used in the bottom chamber as a negative control (neg CT). Data are representative of three independent experimental repeats.

Although recent clinical trials of KRAS^G12C^ inhibition have shown modest efficacy in *KRAS*^G12C^MT colorectal cancer, emergence of acquired resistance limits further clinical benefit (28). To identify mechanisms underlying acquired drug resistance to KRAS^G12C^ inhibition and therapeutic strategies to overcome this limitation, we generated a preclinical AZ’1569-resistant colorectal cancer model. We selected the RW7213 cell line, which shows the highest sensitivity to AZ’1569 and cultured this cell line until resistant derivatives and/or clones emerged in the presence of AZ’1569. Three independent resistant (R) RW7213 cell populations were obtained, and these were therefore indicated as resistant No. 2, No. 3, and No. 4 (Supplementary Fig. S6A). Resistance to AZ’1569 was confirmed by cell viability assays comparing parental and resistant cell derivatives (Fig. 6A). All resistant models also showed cross-resistance to the KRAS^G12C^ inhibitors sotorasib and adagrasib (MRTX849; Fig. 6A; Supplementary Fig. S6A). Notably, both AZ’1569 and sotorasib seemed to increase the growth rate of AZ’1569-R clones, suggesting that AZ’1569-R clones had become addicted to the presence of KRAS^G12C^ inhibition for proliferation.

### Acquired AZ’1569-R clones display KRAS amplification and activation of several RTKs

Prior studies indicated that tumors with acquired resistance to KRAS^G12C^ agents can have multiple resistance mechanisms, including alterations within the RAS/MAPK pathway and bypass activating alterations (28, 29). Although all resistant cell populations retained the original KRAS^G12C^ mutation, MedExome sequencing of the RW7213-R clones did not reveal any secondary mutations within KRAS, BRAF/MEK/ERK, PIK3CA/AKT (Supplementary Fig. S6B). Notably, total KRAS, KRAS-GTP, and mRNA levels together with pERK1/2 levels were markedly upregulated in the RW7213-R clones (Fig. 6A). Moreover, NGS confirmed that *KRAS* was amplified in all three clones and clone 3 had an additional amplification in EGFR (Supplementary Fig. S6C). Of note, withdrawal of AZ’1569 for 8 weeks resulted in loss of KRAS overexpression, reversal of hyperactivated signaling to ERK1/2, and resensitization to AZ’1569 (Supplementary Fig. S6D).

We also assessed the phosphorylation status of 49 RTKs in parental and AZ’1569-R clones. AZ’1569-R derivatives showed increased phosphorylation of a number of RTKs such as c-MET and EphA2 (Supplementary Fig. S6E); these were validated using Western blotting (WB) analysis (Fig. 6A). Collectively, our data revealed KRAS amplification and coincidental bypass RTK acquired alterations in our AZ’1569-resistant clones, suggesting that the cell models generated in this work have the potential to recapitulate clinically relevant resistance mechanisms.

### Acquired AZ’1569-R clones driven by KRAS amplification represents a therapeutic challenge

Classically, the identification of molecular mechanisms underlying acquired resistance should enable rational intervention with small-molecule inhibitors to overcome resistance. On the basis of the results from our RTK array validation, we used a focused drug screen targeting KRAS/ERK signaling, including SOS1-KRAS inhibitor BI-3406, SHP2 inhibitor SHP-099, MEK1/2 inhibitor trametinib, and ERK1/2 inhibitor ulixertinib. We also used inhibitors of AKT (capivasertib), S6K (PF-4708671), JAK/STAT3 (AZD1480), EphA2 (dasatinib), c-MET (crizotinib) kinases, and BCL-2/Bcl-xL inhibitors ABT-737 and sabutoclax (Fig. 6B). Bcl-xL inhibition did not affect survival of AZ’1569-R clones (Fig. 6B; Supplementary Fig. S6F). Surprisingly, we found that AZ’1569-R derivatives displayed reduced sensitivity to the diverse kinase inhibitors compared with their parental counterpart. Moreover, AZ’1569-R clones were also more resistant to the chemotherapeutic agents 5-FU, SN-38, and oxaliplatin. As expected, all AZ’1569-R clones were highly resistant to cetuximab.

### Acquired AZ’1569-R clones overproduce a wide array of proinflammatory factors

We have previously shown that oncogenic KRAS regulates growth factor and/or cytokine shedding and ADAM17 activity (30), a protease involved in acute resistance to chemotherapy and targeted therapies (31). We therefore investigated the growth factors/cytokines released by the AZ’1569-resistant cells, using a cytokine array (Fig. 6C). Of the 105 cytokines examined by the array, 15 targets were >1.5-fold upregulated in all three resistant clones, and these included cytokines and/or chemokines involved in innate/adaptive immunity (e.g., IL8, CXCL1) and growth factors (e.g., TGFα). We validated our array results using real-time PCR and/or specific ELISAs, showing that AZ’1569-resistant clones exhibited higher levels of IL8, CXCL1, IFNγ, and TGFα (Fig. 6C; Supplementary Fig. S6G and S6H). Given the marked cytokine and/or chemokine abundance in the drug-resistant lines, we also determined PD-L1 levels and found >300-fold increased levels of *CD274* (encoding PD-L1) in the AZ’1569-R clones (Fig. 6C). Of note, conditioned medium of all three AZ’1569-R clones markedly reduced sensitivity of parental RW7213 cells to AZ’1569 (Fig. 6D). In addition, exposure to conditioned medium of all three AZ’1569-R clones increased peripheral blood mononuclear cell (PBMC) migration, indicating their importance for lymphocytic infiltration (Fig. 6D). Taken together, these results show that long-term exposure to AZ’1569 dramatically increases the immunogenicity of these cells and suggests that the induction of proinflammatory factors may produce a tumor microenvironment that is conducive to increased tumor infiltration by immune cells.

## Discussion

The efficacy of anticancer targeted therapies has often been compromised by the occurrence of intrinsic and acquired resistance mechanisms, involving intratumoral heterogeneity and various compensatory signaling. Recently, drugs such as sotorasib and adagrasib, which inhibit KRAS^G12C^, have emerged as promising targeted therapies for patients with *KRAS*^G12C^MT lung cancer (32, 33). However, clinical trials, such as CodeBreaK100 and KRYSTAL-1 using single-agent sotorasib and adagrasib, respectively, have shown substantial differences in response rates between patients with lung cancer and colorectal cancer (10, 34, 35). On the basis of these studies, sotorasib was granted FDA approval, but only for patients with *KRAS*^G12C^MT lung cancer (36). Understanding the factors underlying intrinsic and/or acquired resistance to this new class of compounds is critical, in particular to benefit patients with colorectal cancer. There is a growing body of evidence suggesting that deficient apoptosis induction following targeted therapy treatments can lead to a lack of efficacy (37, 38). We found that KRAS^G12C^ inhibitor monotherapy was relatively ineffective at inducing apoptosis *in vitro* in *KRAS*^G12C^MT colorectal cancer.

There are, as of yet, no available predictive biomarkers for response to KRAS^G12C^ inhibitors. In agreement with previous studies, we found that sensitivity to AZ’1569 was not predicted by the KRAS allele zygosity status, the presence of concomitant mutations (including *TP53* mutations), or baseline levels of kinases within the EGFR/KRAS axis (32, 39). We previously used DR_MOMP, an apoptosis predictor model that requires protein profiling of Bcl-2 family proteins to predict therapeutic response and prognosis in colorectal cancer (16, 27). Although not significant, we found that the Bcl-xL/BAK ratio correlated with response to AZ’1569 treatment. Previous *in vitro* and clinical studies have shown positive correlations between BIM expression and response to anti-EGFR and BRAF drugs in EGFR and BRAF addicted tumors (40, 41). Interestingly, our study showed that pretreatment BIM levels were associated with sensitivity and apoptotic response to AZ’1569 in *KRAS*^G12C^MT colorectal cancer cells. Further biomarker analysis of tissue samples from patients treated with KRAS^G12C^ inhibitors will be needed to confirm the predictive role of BIM.

Previous studies evidenced that RTK/kinase feedback activation is among the main mechanisms of adaptive resistance to KRAS^G12C^ inhibitors and that therefore vertical combinations with RTK/SHP2 inhibitors (42) or kinase inhibitors (e.g., MEK1/2, PI3K/mTORC1/2; refs. 32, 39, 43) are the most attractive combination options. In support of these studies, we found consistent evidence of rapid ERK1/2, AKT, and/or S6 feedback reactivation following AZ’1569 treatment, although this was heterogeneous across the *KRAS*^G12C^MT models. Combinations of AZ’1569 with inhibitors of these feedback loops showed differential effects across the *KRAS*^G12C^MT colorectal cancer cell lines. Conversely, inhibition of EGFR markedly enhanced sensitivity to AZ’1569 in all *KRAS*^G12C^MT colorectal cancer cells, supporting the findings of a recent study (12). However, addition of cetuximab to AZ’1569 only resulted in potent increases in cell death in three of seven *KRAS*^G12C^MT colorectal cancer models.

Using RNA-seq, IPA, and a siRNA screening approach, we identified that *BCL2L1* was a critical mediator of resistance to cell death following KRAS^G12C^ inhibition in colorectal cancer cells. Moreover, using focused drug screens, we identified that the BCL-2/Bcl-xL inhibitor ABT-737 was an effective inducer of apoptosis when combined with AZ’1569 in the panel of *KRAS*^G12C^MT colorectal cancer cells. Treatment with AZ’1569 resulted in acute increases in the proapoptotic protein BIM, which may “prime” cells for death, but was insufficient to cause apoptosis in five of seven *KRAS*^G12C^MT colorectal cancer models due to the presence of inhibitory antiapoptotic proteins, such as Bcl-xL. Consistent with previous studies, we showed that ABT-737 abrogates the inhibitory complex between Bcl-xL and BIM (Supplementary Fig. S3D; ref. 44), leading to robust increases in apoptosis when ABT-737 was combined with KRAS^G12C^ inhibition in our study. Alongside its pivotal role in regulating MOMP, Bcl-xL has been identified as a critical mediator of stem cell survival through the adeno-to-colon carcinoma sequence (45). In addition, a number of studies have shown that Bcl-xL plays an important role in regulating sensitivity to chemotherapy and other targeted therapies (45–47).

The importance of Bcl-xL as a mediator of acute resistance to KRAS^G12C^ inhibitors was shown *in vivo*, where combined treatment of *KRAS*^G12C^MT colorectal cancer xenografts with the BCL-2/Bcl-xL and KRAS^G12C^ inhibitors, navitoclax and AZ’8037, resulted in supraadditive reductions in tumor growth or regression. During this article's preparation, initial results of the phase Ib study of cetuximab with adagrasib were released, showing a response rate of 43% in patients with *KRAS*^G12C^MT colorectal cancer (48). Although initial results of this small study are encouraging, it also suggests that a major part of this population will not respond to this combination, indicating the need for alternative treatment combinations. Our data suggest that combined Bcl-xL/KRAS^G12C^ inhibition is another potential novel treatment strategy for this molecular subgroup of patients with colorectal cancer. Although less well tolerated in our *in vivo* strain, combination treatments with navitoclax have been widely trialed in other *in vivo* strains and patients without major reported toxicities (46, 49). In further support of our data, a recent study showed that the Bcl-xL–targeted PROTAC, DT2216, enhanced the therapeutic efficacy of sotorasib in the SW837 *KRAS*^G12C^MT model, and demonstrated also a good tolerability (50).

Acquired resistance is a major problem limiting clinical efficacy of targeted therapies. We observed amplification of the KRAS^G12C^ allele in all three AZ’1569-acquired resistant clones, which also coincided with acquired bypass activations in a number of RTK. Interestingly, this is consistent with analysis of clinical samples from patients treated in phase I/II studies with adagrasib (28). Contrastingly, no acquired mutations affecting the switch II pocket of KRAS (R68S, H95D/Q/R, Y96C) or other pathogenic mutations in other RTK-RAS-MAPK pathway members were detected. Importantly, we also show that acquired resistance driven by KRAS amplification is reversible upon drug withdrawal, likely because KRAS amplification confers a selective disadvantage in the absence of KRAS^G12C^ inhibition.

The AZ’1569-R cells showed a high level of resistance to a range of targeted therapies, particularly SOS1 and MEK/ERK inhibition. Importantly, AZ’1569-R cells also showed markedly reduced sensitivity to the three chemotherapies used in colorectal cancer treatment. Thus, our results would indicate that, at least in cases where KRAS^G12C^ inhibitor resistance is driven by KRAS amplification, patients who progress following upfront treatment with KRAS^G12C^ inhibition may be poor candidates for other targeted therapies or chemotherapies. A previous study has shown that sotorasib has an early impact on tumor immune cell infiltration (33). Interestingly, our acquired AZ’1569-R cells showed a markedly increased proinflammatory cytokine and/or chemokine profile, which resulted in increased lymphocytic infiltration. These data further suggest major changes in the immune microenvironment of KRAS^G12C^ inhibitor–resistant tumors, which may affect their response to immune-targeted therapies.

In conclusion, using a systems biology approach, we identified Bcl-xL as an important mediator of intrinsic resistance to KRAS^G12C^ inhibition in *KRAS^G12C^*MT colorectal cancer. We show that KRAS^G12C^ inhibition primes cells for death through acute induction of BIM, with coneutralization of Bcl-xL resulting in potent increases in cell death. From a cancer therapeutics perspective, the substantial tumor growth inhibition observed in our xenografts provides a strong rationale to combine Bcl-xL blockade, using navitoclax or HDAC1–3 inhibition (Supplementary Fig. S4C) with KRAS^G12C^ inhibitors in patients with colorectal cancer. We also demonstrate the importance of drug holidays, to delay and/or overcome emergent resistance to KRAS^G12C^ inhibition. Finally, cross-resistance to other targeted therapies and importantly conventional chemotherapy in the AZ’1569-R cells poses a challenge, with implications for the optimal use of KRAS^G12C^ inhibitors as a second- or third-line option.

## Supplementary Material

Supplementary Data 1 antibodies

Supplementary Data 2 concentrations of drugs

Supplementary Materials and Methods SM1 Supplementary materials and methods

Supplementary Figures S1-S6 Supplementary figures

## References

[1] Cox AD , Fesik SW, Kimmelman AC, Luo J, Der CJ. Drugging the undruggable RAS: mission possible? Nat Rev Drug Discov 2014;13:828–51. 25323927 10.1038/nrd4389 PMC4355017

[2] Prior IA , Hood FE, Hartley JL. The frequency of Ras mutations in cancer. Cancer Res 2020;80:2969–74. 32209560 10.1158/0008-5472.CAN-19-3682 PMC7367715

[3] Simanshu DK , Nissley DV, McCormick F. RAS proteins and their regulators in human disease. Cell 2017;170:17–33. 28666118 10.1016/j.cell.2017.06.009 PMC5555610

[4] Tejpar S , Stintzing S, Ciardiello F, Tabernero J, Van Cutsem E, Beier F, . Prognostic and predictive relevance of primary tumor location in patients with RAS wild-type metastatic colorectal cancer: retrospective analyses of the CRYSTAL and FIRE-3 trials. JAMA Oncol 2017;3:194–201. 27722750 10.1001/jamaoncol.2016.3797 PMC7505121

[5] Kopetz S , Grothey A, Yaeger R, Van Cutsem E, Desai J, Yoshino T, . Encorafenib, binimetinib, and cetuximab in BRAF V600E-mutated colorectal cancer. N Engl J Med 2019;381:1632–43. 31566309 10.1056/NEJMoa1908075

[6] Kubicka S , Greil R, Andre T, Bennouna J, Sastre J, Van Cutsem E, . Bevacizumab plus chemotherapy continued beyond first progression in patients with metastatic colorectal cancer previously treated with bevacizumab plus chemotherapy: ML18147 study KRAS subgroup findings. Ann Oncol 2013;24:2342–9. 23852309 10.1093/annonc/mdt231

[7] Ostrem JM , Peters U, Sos ML, Wells JA, Shokat KM. K-Ras(G12C) inhibitors allosterically control GTP affinity and effector interactions. Nature 2013;503:548–51. 24256730 10.1038/nature12796 PMC4274051

[8] Blons H , Emile JF, Le Malicot K, Julie C, Zaanan A, Tabernero J, . Prognostic value of KRAS mutations in stage III colon cancer: post hoc analysis of the PETACC8 phase III trial dataset. Ann Oncol 2014;25:2378–85. 25294886 10.1093/annonc/mdu464

[9] Dunnett-Kane V , Burkitt-Wright E, Blackhall FH, Malliri A, Evans DG, Lindsay CR. Germline and sporadic cancers driven by the RAS pathway: parallels and contrasts. Ann Oncol 2020;31:873–83. 32240795 10.1016/j.annonc.2020.03.291 PMC7322396

[10] Hong DS , Fakih MG, Strickler JH, Desai J, Durm GA, Shapiro GI, . KRAS(G12C) inhibition with sotorasib in advanced solid tumors. N Engl J Med 2020;383:1207–17. 32955176 10.1056/NEJMoa1917239 PMC7571518

[11] Kettle JG , Bagal SK, Bickerton S, Bodnarchuk MS, Breed J, Carbajo RJ, . Structure-based design and pharmacokinetic optimization of covalent allosteric inhibitors of the mutant GTPase KRAS(G12C). J Med Chem 2020;63:4468–83. 32023060 10.1021/acs.jmedchem.9b01720

[12] Amodio V , Yaeger R, Arcella P, Cancelliere C, Lamba S, Lorenzato A, . EGFR blockade reverts resistance to KRAS(G12C) inhibition in colorectal cancer. Cancer Discov 2020;10:1129–39. 32430388 10.1158/2159-8290.CD-20-0187 PMC7416460

[13] Tibbetts LM , Chu MY, Vezeridis MP, Miller PG, Tibbetts LL, Poisson MH, . Cell culture of the mucinous variant of human colorectal carcinoma. Cancer Res 1988;48:3751–9. 2837323

[14] Van Schaeybroeck S , Kalimutho M, Dunne PD, Carson R, Allen W, Jithesh PV, . ADAM17-dependent c-MET-STAT3 signaling mediates resistance to MEK inhibitors in KRAS mutant colorectal cancer. Cell Rep 2014;7:1940–55. 24931611 10.1016/j.celrep.2014.05.032

[15] Khawaja H , Campbell A, Roberts JZ, Javadi A, O'Reilly P, McArt D, . RALB GTPase: a critical regulator of DR5 expression and TRAIL sensitivity in KRAS mutant colorectal cancer. Cell Death Dis 2020;11:930. 33122623 10.1038/s41419-020-03131-3 PMC7596570

[16] Lindner AU , Concannon CG, Boukes GJ, Cannon MD, Llambi F, Ryan D, . Systems analysis of BCL2 protein family interactions establishes a model to predict responses to chemotherapy. Cancer Res 2013;73:519–28. 23329644 10.1158/0008-5472.CAN-12-2269

[17] Bradley CA , Dunne PD, Bingham V, McQuaid S, Khawaja H, Craig S, . Transcriptional upregulation of c-MET is associated with invasion and tumor budding in colorectal cancer. Oncotarget 2016;7:78932–45. 27793046 10.18632/oncotarget.12933 PMC5346688

[18] Chou TC , Talalay P. Quantitative analysis of dose-effect relationships: the combined effects of multiple drugs or enzyme inhibitors. Adv Enzyme Regul 1984;22:27–55. 6382953 10.1016/0065-2571(84)90007-4

[19] Romanelli S , Perego P, Pratesi G, Carenini N, Tortoreto M, Zunino F. *In vitro* and *in vivo* interaction between cisplatin and topotecan in ovarian carcinoma systems. Cancer Chemother Pharmacol 1998;41:385–90. 9523734 10.1007/s002800050755

[20] Medico E , Russo M, Picco G, Cancelliere C, Valtorta E, Corti G, . The molecular landscape of colorectal cancer cell lines unveils clinically actionable kinase targets. Nat Commun 2015;6:7002. 25926053 10.1038/ncomms8002

[21] Mouradov D , Sloggett C, Jorissen RN, Love CG, Li S, Burgess AW, . Colorectal cancer cell lines are representative models of the main molecular subtypes of primary cancer. Cancer Res 2014;74:3238–47. 24755471 10.1158/0008-5472.CAN-14-0013

[22] Ku JL , Shin YK, Kim DW, Kim KH, Choi JS, Hong SH, . Establishment and characterization of 13 human colorectal carcinoma cell lines: mutations of genes and expressions of drug-sensitivity genes and cancer stem cell markers. Carcinogenesis 2010;31:1003–9. 20176655 10.1093/carcin/bgq043

[23] Liu Y , Bodmer WF. Analysis of P53 mutations and their expression in 56 colorectal cancer cell lines. Proc Natl Acad Sci U S A 2006;103:976–81. 16418264 10.1073/pnas.0510146103 PMC1327731

[24] Guinney J , Dienstmann R, Wang X, de Reynies A, Schlicker A, Soneson C, . The consensus molecular subtypes of colorectal cancer. Nat Med 2015;21:1350–6. 26457759 10.1038/nm.3967 PMC4636487

[25] Zeng M , Lu J, Li L, Feru F, Quan C, Gero TW, . Potent and selective covalent quinazoline inhibitors of KRAS G12C. Cell Chem Biol 2017;24:1005–16. 28781124 10.1016/j.chembiol.2017.06.017

[26] Rixe O , Fojo T. Is cell death a critical end point for anticancer therapies or is cytostasis sufficient? Clin Cancer Res 2007;13:7280–7. 18094408 10.1158/1078-0432.CCR-07-2141

[27] Lindner AU , Salvucci M, Morgan C, Monsefi N, Resler AJ, Cremona M, . BCL-2 system analysis identifies high-risk colorectal cancer patients. Gut 2017;66:2141–8. 27663504 10.1136/gutjnl-2016-312287

[28] Awad MM , Liu S, Rybkin II, Arbour KC, Dilly J, Zhu VW, . Acquired resistance to KRAS(G12C) inhibition in cancer. N Engl J Med 2021;384:2382–93. 34161704 10.1056/NEJMoa2105281 PMC8864540

[29] Tanaka N , Lin JJ, Li C, Ryan MB, Zhang J, Kiedrowski LA, . Clinical acquired resistance to KRAS(G12C) inhibition through a novel KRAS switch-II pocket mutation and polyclonal alterations converging on RAS-MAPK reactivation. Cancer Discov 2021;11:1913–22. 33824136 10.1158/2159-8290.CD-21-0365 PMC8338755

[30] Van Schaeybroeck S , Kyula JN, Fenton A, Fenning CS, Sasazuki T, Shirasawa S, . Oncogenic Kras promotes chemotherapy-induced growth factor shedding via ADAM17. Cancer Res 2011;71:1071–80. 21148749 10.1158/0008-5472.CAN-10-0714 PMC3073126

[31] Kyula JN , Van Schaeybroeck S, Doherty J, Fenning CS, Longley DB, Johnston PG. Chemotherapy-induced activation of ADAM-17: a novel mechanism of drug resistance in colorectal cancer. Clin Cancer Res 2010;16:3378–89. 20570921 10.1158/1078-0432.CCR-10-0014 PMC2896550

[32] Hallin J , Engstrom LD, Hargis L, Calinisan A, Aranda R, Briere DM, . The KRAS(G12C) inhibitor MRTX849 provides insight toward therapeutic susceptibility of KRAS-mutant cancers in mouse models and patients. Cancer Discov 2020;10:54–71. 31658955 10.1158/2159-8290.CD-19-1167 PMC6954325

[33] Canon J , Rex K, Saiki AY, Mohr C, Cooke K, Bagal D, . The clinical KRAS(G12C) inhibitor AMG 510 drives anti-tumour immunity. Nature 2019;575:217–23. 31666701 10.1038/s41586-019-1694-1

[34] Jänne PA , Rybkin II, Spira AI, Riely GJ, Papadopoulos KP, Sabari JK, . KRYSTAL-1: activity and safety of adagrasib (MRTX849) in advanced/metastatic non–small-cell lung cancer (NSCLC) harboring KRAS G12C mutation. Eur J Cancer 2020;138:S1–2.

[35] Johnson ML , Ignatius Ou S-H, Barve M, Rybkin II, Papadopoulos KP, Leal TA, . KRYSTAL-1: activity and safety of adagrasib (MRTX849) in patients with colorectal cancer (CRC) and other solid tumors harboring a KRAS G12C mutation. Eur J Cancer 2020;138:S2.

[36] Blair HA . Sotorasib: first approval. Drugs 2021;81:1573–9. 34357500 10.1007/s40265-021-01574-2 PMC8531079

[37] Vaishnavi A , Scherzer MT, Kinsey CG, Parkman GL, Truong A, Ghazi P, . Inhibition of MEK1/2 forestalls the onset of acquired resistance to entrectinib in multiple models of NTRK1-driven cancer. Cell Rep 2020;32:107994. 32755586 10.1016/j.celrep.2020.107994 PMC7478141

[38] Faber AC , Farago AF, Costa C, Dastur A, Gomez-Caraballo M, Robbins R, . Assessment of ABT-263 activity across a cancer cell line collection leads to a potent combination therapy for small-cell lung cancer. Proc Natl Acad Sci U S A 2015;112:E1288–96. 25737542 10.1073/pnas.1411848112 PMC4371986

[39] Misale S , Fatherree JP, Cortez E, Li C, Bilton S, Timonina D, . KRAS G12C NSCLC models are sensitive to direct targeting of KRAS in combination with PI3K inhibition. Clin Cancer Res 2019;25:796–807. 30327306 10.1158/1078-0432.CCR-18-0368

[40] Faber AC , Corcoran RB, Ebi H, Sequist LV, Waltman BA, Chung E, . BIM expression in treatment-naive cancers predicts responsiveness to kinase inhibitors. Cancer Discov 2011;1:352–65. 22145099 10.1158/2159-8290.CD-11-0106 PMC3229203

[41] Costa C , Molina MA, Drozdowskyj A, Gimenez-Capitan A, Bertran-Alamillo J, Karachaliou N, . The impact of EGFR T790M mutations and BIM mRNA expression on outcome in patients with EGFR-mutant NSCLC treated with erlotinib or chemotherapy in the randomized phase III EURTAC trial. Clin Cancer Res 2014;20:2001–10. 24493829 10.1158/1078-0432.CCR-13-2233

[42] Ryan MB , Fece de la Cruz F, Phat S, Myers DT, Wong E, Shahzade HA, . Vertical pathway inhibition overcomes adaptive feedback resistance to KRAS(G12C) inhibition. Clin Cancer Res 2020;26:1633–43. 31776128 10.1158/1078-0432.CCR-19-3523 PMC7124991

[43] Molina-Arcas M , Moore C, Rana S, van Maldegem F, Mugarza E, Romero-Clavijo P, . Development of combination therapies to maximize the impact of KRAS-G12C inhibitors in lung cancer. Sci Transl Med 2019;11:eaaw7999. 31534020 10.1126/scitranslmed.aaw7999 PMC6764843

[44] Weber K , Harper N, Schwabe J, Cohen GM. BIM-mediated membrane insertion of the BAK pore domain is an essential requirement for apoptosis. Cell Rep 2013;5:409–20. 24120870 10.1016/j.celrep.2013.09.010 PMC3898696

[45] Ramesh P , Lannagan TRM, Jackstadt R, Atencia Taboada L, Lansu N, Wirapati P, . BCL-XL is crucial for progression through the adenoma-to-carcinoma sequence of colorectal cancer. Cell Death Differ 2021;28:3282–96. 34117376 10.1038/s41418-021-00816-w PMC8630104

[46] Corcoran RB , Cheng KA, Hata AN, Faber AC, Ebi H, Coffee EM, . Synthetic lethal interaction of combined BCL-XL and MEK inhibition promotes tumor regressions in KRAS mutant cancer models. Cancer Cell 2013;23:121–8. 23245996 10.1016/j.ccr.2012.11.007 PMC3667614

[47] Minn AJ , Rudin CM, Boise LH, Thompson CB. Expression of bcl-xL can confer a multidrug resistance phenotype. Blood 1995;86:1903–10. 7655019

[48] Weiss J Yaeger RD , Johnson ML, Spira A, Klempner SJ, Barve MA, . KRYSTAL-1: Adagrasib (MRTX849) as monotherapy or combined with cetuximab (Cetux) in patients (Pts) with colorectal cancer (CRC) harboring a KRASG12C mutation. Ann Oncol 2021;32:S1283–1346.

[49] Corcoran RB , Do KT, Cleary JM, Parikh AR, Yeku OO, Weekes CD, . Phase I/II study of combined BCL-XL and MEK inhibition with navitoclax (N) and trametinib (T) in KRAS or NRAS mutant advanced solid tumours. Ann Oncol 2019;30:v164. 10.1158/1078-0432.CCR-23-3135 PMC11061595 38456660

[50] Khan S , Wiegand J, Zhang P, Hu W, Thummuri D, Budamagunta V, . BCL-XL PROTAC degrader DT2216 synergizes with sotorasib in preclinical models of KRAS(G12C)-mutated cancers. J Hematol Oncol 2022;15:23. 35260176 10.1186/s13045-022-01241-3 PMC8905794

