# Supplementary material for: Bcl-xL Is a Key Mediator of Apoptosis Following KRASG12C Inhibition in KRASG12C-mutant Colorectal Cancer
Source: Mol Cancer Ther. 2022 Oct 22;22(1):135–49. doi: 10.1158/1535-7163.MCT-22-0301 (PMC9808374; doi:10.1158/1535-7163.MCT-22-0301)
Supplement: Supplementary Materials and Methods SM1 — Supplementary materials and methods [file mct-22-0301_supplementary_materials_and_methods_sm1_suppsm1.docx]

**Bcl-xL** **is a key mediator of apoptosis following KRAS^G12C^ inhibition in *KRAS^G12C^* mutant colorectal cancer.**

**SUPPLEMENTARY MATERIALS AND METHODS**

**Non-FDA approved compounds**

Non-FDA approved compounds used in this manuscript include AZD1480 (1), Capivasertib (AZD5363) (2), ABT-737 (3), S6K-18 (4), ABT-263 (5), BI-3406 (6) , SHP-099 (7), Ulixertinib (8), PF-4708671 (9), Sabutoclax (10), Entinostat (11), Saracatinib (AZD0530) (12), Alisertib (13), Roscovitine (14), Nutlin-3 (15), Barasertib (16), Ganetespib (STA-9090) (17), BIBR-1532 (18), Epothilone A (19), AZD7762 (20), Degrasyn (WP1130) (21), AT-406 (SM-406)/Xevinapant (22), I-BET151 (23), Sirtinol (24), IMD-0354 (25), Salubrinal (26), JNK-IN-8 (27), Birinapant (28), RG-7112 (29), AZD1208 (30), UNC1999 (31), Tasisulam (32), TH287 (33), AZD6738 (34), CB-5083 (35), AZD1390 (36), CX-5461 (37), NU7026 (38), Palifosfamide (39), EPZ5676 (40), AZD5991 (41), AZD4573 (42), ONC-206 (43) and IZ-TRAIL (44).

**Cell culture**

SNU1411 and LIM2099 were cultured in RPMI-1640, C106 in IMDM, RW7213 in DMEM, SW837 and SW1463 in DMEM/F12 and V481 in MEM2+. Each media was supplemented with 10% FCS and 1mM sodium pyruvate. V481 cells were cultured in MEM2+, supplemented with 1µg/ml hydrocortisone, 10µg/ml Insulin, 2µg/ml Apotransferrin and 0.86ng/ml Sodium Selenite (Merck).

**DNA expression constructs**

Myc-tagged Bcl-xL was purchased from Sino Biological (China). FLAG-MCL-1 (Addgene plasmid#25392) and Flag-BCL2 (Addgene plasmid#18003) were gifts from Roger Davis and Clark Distelhorst, respectively (45).

**Primers**

Primers were purchased from Eurofins Genomics.

RT-PCR

*IFNG* Forward: 5’-TGGCTTTTCAGCTCTGCATC-3’, Reverse: 5’-CCGCTACATCTGAATGACCTG -3’;

Sanger sequencing

KRAS primers used here have been described in a previous study (46).

*KRAS* exon 2 FW: GGTGGAGTATTTGATAGTGTATTAACC

*KRAS* exon 2 RV: AGAATGGTCCTGCACCAGTAA

*KRAS* exon 3 FW: AAAGGTGCACTGTAATAATCCAGAC

*KRAS* exon 3 RV: ATGCATGGCATTAGCAAAGA

*KRAS* exon 4 FW: TGGACAGGTTTTGAAAGATATTTG

*KRAS* exon 4 RV: ATTAAGAAGCAATGCCCTCTCAAG

**Migration assays**

5µm Transwell polycarbonate membrane inserts from Corning were used. 2.5x10^5^ peripheral blood mononuclear cells (PBMCs) were re-suspended in 200µl of 2% FCS-supplemented DMEM and were added to the top chamber. The bottom chamber was filled with 750µl conditioned medium (2% FCS-supplemented DMEM) obtained from RW7213 parental or resistant cells. Cells were incubated for four hours, following which CellTiter-Glo® was used to measure PBMC migration to the bottom chamber. Serum-free DMEM was used as a negative control. PBMCs were obtained from a healthy donor with written consent and ethical approval granted by the Northern Ireland Blood Transfusion Service, and were provided by Ms. Anne Jordan.

**Sequencing**

RNA, MedExome and NGS sequencing was performed by the Genomics Core Technology Unit and the Precision Medicine Centre for Excellence, respectively, at Queen’s University Belfast.

RNA-sequencing and analysis (GSE198530)

Bulk RNA-sequencing was performed for SW837 and SNU1411 cells treated with 1µM AZ’1569 for 6, 12 and 24 hours. Three biological replicates for each sample were included in the experiment. Total RNA was extracted as described in the main materials and methods section. Each sample underwent QC, and library preparation was performed with 100ng RNA using the KAPA RNA HyperPrep Kit with RiboErase (HMR) (Roche, USA). Libraries were pooled, underwent QC and were sequenced on a NextSeq 500 using a 150 cycle High Output kit (Illumina, USA) to yield >15M paired end 75bp reads per sample.

Generated FASTQ files were analysed using a workflow on Partek® Flow software, v10.0. Briefly, FASTQ files were imported into the software and assigned associated attributes (cell line and time-point), and were aligned with STAR (v2.7.3a) using default settings (assembly=human genome hg38, aligner index=whole genome). Post-alignment QA/QC and quantification of aligned reads to an annotation model (Partek E/M, default settings; min reads=10) was performed, generating Ensembl counts. Differential expression analysis was performed using the GSA (gene specific analysis) tool, with in-built default normalisation applied (normalised to total count, CPM, add 0.0001). ‘Time-point’ and ‘cell line’ were chosen as attributes to compare for GSA analysis. The resulting gene lists were downloaded and imported into Ingenuity Pathway Analysis (IPA^®^) software (Qiagen, UK) to identify significantly enriched pathways for each time-point following AZ’1569 treatment in both cell lines. A cut-off threshold of fold-change >1.3 or <-1.3, and p-value<0.05 was applied to gene lists for pathway analysis. Comparison analysis in IPA^®^ was used to compare significantly enriched (-log_10_ p-value ≥1.3) pathways across both cell lines and all time-points. For volcano plots, log_2_ratios of time-point vs. control and -log_10_ p-values for genes were plotted on the x- and y-axis respectively, in Prism 9.0.

MedExome sequencing and analysis (PRJNA815942)

DNA was extracted from RW7213 parental and resistant cells as described in the main materials and methods section. Targeted DNA-sequencing was performed with 200ng genomic DNA using the Roche KAPA HyperPlus kit (Roche, USA) for whole genome library preparation and Roche SeqCap EZ MedExome Probes in conjunction with the HyperCap Target Enrichment Kit (Roche) to enrich for targets. This kit enriches coding exons and 20bp of the flanking intronic sequences from around 4600 protein-coding genes, including medically relevant genes (47). Sequencing was performed on the Illumina Novaseq 6000 with a SP 200 cycle reagent kit (Illumina, USA), applying paired end sequencing for 2x 100bp reads.

FASTQ files were aligned to the hg38 genome, sorted, and indexed with bwa mem and samtools (48). Duplicates were marked and all reads assigned to a new read group with Picard (49). Somatic short variants were identified from the samples with a matched normal using GATK (50) (v4.1.9.0) best practices workflow (51, 52). GATK base quality score recalibration was applied, and variants were called with Mutect2. Variants were filtered for contamination and orientation bias artefacts, including standard hard filtering, with FilterMutectCalls as per guidelines. On-target and pass variants were annotated with ANNOVAR (53) for further downstream analysis. VCF files were converted to MAF format using vcf2maf (54) which uses VEP (55). Further analysis and visualisation of MAF files was conducted using maftools (56).

Next Generation Sequencing (NGS) and analysis

DNA was extracted from RW7213 parental and AZ’1569-resistant cells as described in the main methods section. 200ng genomic DNA was used as the input for NGS libraries using the KAPA HyperPlus Kit (Roche Sequencing Solutions, Pleasanton, CA, US) according to the manufacturer’s instructions and in-house SOPs. Prepared DNA libraries were hybridised with a custom-designed hybrid-capture next generation sequencing panel (Roche Sequencing Solutions). Following hybridisation, samples were sequenced on a NextSeq 500 (Illumina, San Diego, CA, USA) using the NextSeq 550 System Mid-Output Kit v2 (150 cycles) with 76bp paired-end reads. Binary base call (BCL) files were processed using custom-designed in-house bioinformatics pipelines which converted BCL files to FASTQ files, in accordance with Genome Analysis Toolkit (GATK) best practices (50), including alignment to the hg38 build of the human reference genome using the Burrows-Wheeler Alignment (BWA) tool. Following deduplication and quality scoring, variant calling was performed using Vardict (57) for SNP/INDEL variant calling, Manta for structural variant calling (58), CNVPanelizer (59) for copy number estimation and MSIsensor-Pro (60) for microsatellite instability assessment. Variant filtering and annotation was performed using SnpEff (61) and customised scripts developed specifically for this assay.

***In vivo* study**

Growth curve studies

Tumour growth was assessed following injection of 2.5 x10^6^ and 5x10^6^ SNU1411, 7.5 x10^6^ and 10x10^6^ SW837 and 7.5x10^6^ and 10x10^6^ SW1463 cells in the left and right flank of mice respectively. Mice weight and xenograft growth (using the formula: *(shortest tumour diameter)^2^ × longest tumour diameter × 0.5*) was monitored 3 times per week. Mice were sacrificed when combined tumour volume reached 1200mm^3^ or 1 month following cell inoculation. In contrast to the SW837 model, SNU1411 and SW1463 xenografts both showed exponential growth (Supplementary Fig. S5A) and hence were taken forward in the efficacy study.

Tolerability study

The maximum tolerated dose of KRAS^G12C^ inhibitor AZ’8037 (oral gavage; 50 or 100mg/kg) in combination with Navitoclax (oral gavage; 50, 75 or 100mg/kg), was assessed in NOD/SCID mice in a dose-escalation study. Navitoclax was formulated in 10% ethanol, 30% polyethylene glycol 400, and 60% Phosal 50 PG and AZ’8037 in 1% (w/v) Pluronic F127. A single dose of the lowest dose of AZ’8037 50mg/kg PO (oral gavage), was combined with Navitoclax (50mg/kg). Doses were increased if no adverse events were noticed after 2 treatments with an interval of three days (to explore late toxicity) on the lower dose. Outward signs of distress and mouse weight were monitored daily was monitored. MTD was defined as the maximal dose of both drugs in combination which does not results in weight loss > 15% or death (Supplementary Fig. S5B).

**REFERENCES**

1. Hedvat M, Huszar D, Herrmann A, Gozgit JM, Schroeder A, Sheehy A , et al. The JAK2 inhibitor AZD1480 potently blocks Stat3 signaling and oncogenesis in solid tumors. Cancer Cell 2009; 16: 487-97.

2. Davies BR, Greenwood H, Dudley P, Crafter C, Yu DH, Zhang J , et al. Preclinical pharmacology of AZD5363, an inhibitor of AKT: pharmacodynamics, antitumor activity, and correlation of monotherapy activity with genetic background. Mol Cancer Ther 2012; 11: 873-87.

3. Oltersdorf T, Elmore SW, Shoemaker AR, Armstrong RC, Augeri DJ, Belli BA , et al. An inhibitor of Bcl-2 family proteins induces regression of solid tumours. Nature 2005; 435: 677-81.

4. <https://pubchem.ncbi.nlm.nih.gov/compound/53317853>.

5. Lam LT, Lu X, Zhang H, Lesniewski R, Rosenberg S,Semizarov D. A microRNA screen to identify modulators of sensitivity to BCL2 inhibitor ABT-263 (navitoclax). Mol Cancer Ther 2010; 9: 2943-50.

6. Hofmann MH, Gmachl M, Ramharter J, Savarese F, Gerlach D, Marszalek JR , et al. BI-3406, a Potent and Selective SOS1-KRAS Interaction Inhibitor, Is Effective in KRAS-Driven Cancers through Combined MEK Inhibition. Cancer discovery 2021; 11: 142-57.

7. Garcia Fortanet J, Chen CH, Chen YN, Chen Z, Deng Z, Firestone B , et al. Allosteric Inhibition of SHP2: Identification of a Potent, Selective, and Orally Efficacious Phosphatase Inhibitor. Journal of medicinal chemistry 2016; 59: 7773-82.

8. Ward RA, Colclough N, Challinor M, Debreczeni JE, Eckersley K, Fairley G , et al. Structure-Guided Design of Highly Selective and Potent Covalent Inhibitors of ERK1/2. Journal of medicinal chemistry 2015; 58: 4790-801.

9. Pearce LR, Alton GR, Richter DT, Kath JC, Lingardo L, Chapman J , et al. Characterization of PF-4708671, a novel and highly specific inhibitor of p70 ribosomal S6 kinase (S6K1). Biochem J 2010; 431: 245-55.

10. Wei J, Stebbins JL, Kitada S, Dash R, Placzek W, Rega MF , et al. BI-97C1, an optically pure Apogossypol derivative as pan-active inhibitor of antiapoptotic B-cell lymphoma/leukemia-2 (Bcl-2) family proteins. Journal of medicinal chemistry 2010; 53: 4166-76.

11. Saito A, Yamashita T, Mariko Y, Nosaka Y, Tsuchiya K, Ando T , et al. A synthetic inhibitor of histone deacetylase, MS-27-275, with marked in vivo antitumor activity against human tumors. Proc Natl Acad Sci U S A 1999; 96: 4592-7.

12. Chang YM, Bai L, Liu S, Yang JC, Kung HJ,Evans CP. Src family kinase oncogenic potential and pathways in prostate cancer as revealed by AZD0530. Oncogene 2008; 27: 6365-75.

13. Manfredi MG, Ecsedy JA, Chakravarty A, Silverman L, Zhang M, Hoar KM , et al. Characterization of Alisertib (MLN8237), an investigational small-molecule inhibitor of aurora A kinase using novel in vivo pharmacodynamic assays. Clin Cancer Res 2011; 17: 7614-24.

14. Havlicek L, Hanus J, Vesely J, Leclerc S, Meijer L, Shaw G , et al. Cytokinin-derived cyclin-dependent kinase inhibitors: synthesis and cdc2 inhibitory activity of olomoucine and related compounds. Journal of medicinal chemistry 1997; 40: 408-12.

15. Vassilev LT, Vu BT, Graves B, Carvajal D, Podlaski F, Filipovic Z , et al. In vivo activation of the p53 pathway by small-molecule antagonists of MDM2. Science 2004; 303: 844-8.

16. Mortlock AA, Foote KM, Heron NM, Jung FH, Pasquet G, Lohmann JJ , et al. Discovery, synthesis, and in vivo activity of a new class of pyrazoloquinazolines as selective inhibitors of aurora B kinase. Journal of medicinal chemistry 2007; 50: 2213-24.

17. Goldman JW, Raju RN, Gordon GA, El-Hariry I, Teofilivici F, Vukovic VM , et al. A first in human, safety, pharmacokinetics, and clinical activity phase I study of once weekly administration of the Hsp90 inhibitor ganetespib (STA-9090) in patients with solid malignancies. BMC Cancer 2013; 13: 152.

18. Damm K, Hemmann U, Garin-Chesa P, Hauel N, Kauffmann I, Priepke H , et al. A highly selective telomerase inhibitor limiting human cancer cell proliferation. EMBO J 2001; 20: 6958-68.

19. Meng D, Sorensen EJ, Bertinato P,Danishefsky SJ. Studies toward a Synthesis of Epothilone A: Use of Hydropyran Templates for the Management of Acyclic Stereochemical Relationships. J Org Chem 1996; 61: 7998-9.

20. Zabludoff SD, Deng C, Grondine MR, Sheehy AM, Ashwell S, Caleb BL , et al. AZD7762, a novel checkpoint kinase inhibitor, drives checkpoint abrogation and potentiates DNA-targeted therapies. Mol Cancer Ther 2008; 7: 2955-66.

21. Bartholomeusz GA, Talpaz M, Kapuria V, Kong LY, Wang S, Estrov Z , et al. Activation of a novel Bcr/Abl destruction pathway by WP1130 induces apoptosis of chronic myelogenous leukemia cells. Blood 2007; 109: 3470-8.

22. Cai Q, Sun H, Peng Y, Lu J, Nikolovska-Coleska Z, McEachern D , et al. A potent and orally active antagonist (SM-406/AT-406) of multiple inhibitor of apoptosis proteins (IAPs) in clinical development for cancer treatment. Journal of medicinal chemistry 2011; 54: 2714-26.

23. Dawson MA, Prinjha RK, Dittmann A, Giotopoulos G, Bantscheff M, Chan WI , et al. Inhibition of BET recruitment to chromatin as an effective treatment for MLL-fusion leukaemia. Nature 2011; 478: 529-33.

24. Grozinger CM, Chao ED, Blackwell HE, Moazed D,Schreiber SL. Identification of a class of small molecule inhibitors of the sirtuin family of NAD-dependent deacetylases by phenotypic screening. J Biol Chem 2001; 276: 38837-43.

25. Tanaka A, Konno M, Muto S, Kambe N, Morii E, Nakahata T , et al. A novel NF-kappaB inhibitor, IMD-0354, suppresses neoplastic proliferation of human mast cells with constitutively activated c-kit receptors. Blood 2005; 105: 2324-31.

26. Boyce M, Bryant KF, Jousse C, Long K, Harding HP, Scheuner D , et al. A selective inhibitor of eIF2alpha dephosphorylation protects cells from ER stress. Science 2005; 307: 935-9.

27. Zhang T, Inesta-Vaquera F, Niepel M, Zhang J, Ficarro SB, Machleidt T , et al. Discovery of potent and selective covalent inhibitors of JNK. Chemistry & biology 2012; 19: 140-54.

28. Benetatos CA, Mitsuuchi Y, Burns JM, Neiman EM, Condon SM, Yu G , et al. Birinapant (TL32711), a bivalent SMAC mimetic, targets TRAF2-associated cIAPs, abrogates TNF-induced NF-kappaB activation, and is active in patient-derived xenograft models. Mol Cancer Ther 2014; 13: 867-79.

29. Vu B, Wovkulich P, Pizzolato G, Lovey A, Ding Q, Jiang N , et al. Discovery of RG7112: A Small-Molecule MDM2 Inhibitor in Clinical Development. ACS medicinal chemistry letters 2013; 4: 466-9.

30. Keeton EK, McEachern K, Dillman KS, Palakurthi S, Cao Y, Grondine MR , et al. AZD1208, a potent and selective pan-Pim kinase inhibitor, demonstrates efficacy in preclinical models of acute myeloid leukemia. Blood 2014; 123: 905-13.

31. Konze KD, Ma A, Li F, Barsyte-Lovejoy D, Parton T, Macnevin CJ , et al. An orally bioavailable chemical probe of the Lysine Methyltransferases EZH2 and EZH1. ACS Chem Biol 2013; 8: 1324-34.

32. Meier T, Uhlik M, Chintharlapalli S, Dowless M, Van Horn R, Stewart J , et al. Tasisulam sodium, an antitumor agent that inhibits mitotic progression and induces vascular normalization. Mol Cancer Ther 2011; 10: 2168-78.

33. Gad H, Koolmeister T, Jemth AS, Eshtad S, Jacques SA, Strom CE , et al. MTH1 inhibition eradicates cancer by preventing sanitation of the dNTP pool. Nature 2014; 508: 215-21.

34. Vendetti FP, Lau A, Schamus S, Conrads TP, O'Connor MJ,Bakkenist CJ. The orally active and bioavailable ATR kinase inhibitor AZD6738 potentiates the anti-tumor effects of cisplatin to resolve ATM-deficient non-small cell lung cancer in vivo. Oncotarget 2015; 6: 44289-305.

35. Zhou HJ, Wang J, Yao B, Wong S, Djakovic S, Kumar B , et al. Discovery of a First-in-Class, Potent, Selective, and Orally Bioavailable Inhibitor of the p97 AAA ATPase (CB-5083). Journal of medicinal chemistry 2015; 58: 9480-97.

36. Durant ST, Zheng L, Wang Y, Chen K, Zhang L, Zhang T , et al. The brain-penetrant clinical ATM inhibitor AZD1390 radiosensitizes and improves survival of preclinical brain tumor models. Sci Adv 2018; 4: eaat1719.

37. Drygin D, Lin A, Bliesath J, Ho CB, O'Brien SE, Proffitt C , et al. Targeting RNA polymerase I with an oral small molecule CX-5461 inhibits ribosomal RNA synthesis and solid tumor growth. Cancer Res 2011; 71: 1418-30.

38. Nutley BP, Smith NF, Hayes A, Kelland LR, Brunton L, Golding BT , et al. Preclinical pharmacokinetics and metabolism of a novel prototype DNA-PK inhibitor NU7026. Br J Cancer 2005; 93: 1011-8.

39. Struck RF, Dykes DJ, Corbett TH, Suling WJ,Trader MW. Isophosphoramide mustard, a metabolite of ifosfamide with activity against murine tumours comparable to cyclophosphamide. Br J Cancer 1983; 47: 15-26.

40. Daigle SR, Olhava EJ, Therkelsen CA, Basavapathruni A, Jin L, Boriack-Sjodin PA , et al. Potent inhibition of DOT1L as treatment of MLL-fusion leukemia. Blood 2013; 122: 1017-25.

41. Tron AE, Belmonte MA, Adam A, Aquila BM, Boise LH, Chiarparin E , et al. Discovery of Mcl-1-specific inhibitor AZD5991 and preclinical activity in multiple myeloma and acute myeloid leukemia. Nature communications 2018; 9: 5341.

42. Cidado J, Boiko S, Proia T, Ferguson D, Criscione SW, San Martin M , et al. AZD4573 Is a Highly Selective CDK9 Inhibitor That Suppresses MCL-1 and Induces Apoptosis in Hematologic Cancer Cells. Clin Cancer Res 2020; 26: 922-34.

43. Wagner J, Kline CL, Ralff MD, Lev A, Lulla A, Zhou L , et al. Preclinical evaluation of the imipridone family, analogs of clinical stage anti-cancer small molecule ONC201, reveals potent anti-cancer effects of ONC212. Cell Cycle 2017; 16: 1790-9.

44. Han JH, Moon AR, Chang JH, Bae J, Choi JM, Lee SH , et al. Potentiation of TRAIL killing activity by multimerization through isoleucine zipper hexamerization motif. BMB Rep 2016; 49: 282-7.

45. Wang NS, Unkila MT, Reineks EZ,Distelhorst CW. Transient expression of wild-type or mitochondrially targeted Bcl-2 induces apoptosis, whereas transient expression of endoplasmic reticulum-targeted Bcl-2 is protective against Bax-induced cell death. J Biol Chem 2001; 276: 44117-28.

46. Oddo D, Sennott EM, Barault L, Valtorta E, Arena S, Cassingena A , et al. Molecular Landscape of Acquired Resistance to Targeted Therapy Combinations in BRAF-Mutant Colorectal Cancer. Cancer Res 2016; 76: 4504-15.

47. Aref-Eshghi E, Kerkhof J, Carere DA, Volodarsky M, Bhai P, Colaiacovo S , et al. Clinical and technical assessment of MedExome vs. NGS panels in patients with suspected genetic disorders in Southwestern Ontario. J Hum Genet 2021; 66: 451-64.

48. Li H, Handsaker B, Wysoker A, Fennell T, Ruan J, Homer N , et al. The Sequence Alignment/Map format and SAMtools. Bioinformatics 2009; 25: 2078-9.

49. Picard Toolkit. [Internet]. Broad Institute, GitHub Repository.; Available from: <https://broadinstitute.github.io/picard/>.

50. McKenna A, Hanna M, Banks E, Sivachenko A, Cibulskis K, Kernytsky A , et al. The Genome Analysis Toolkit: a MapReduce framework for analyzing next-generation DNA sequencing data. Genome research 2010; 20: 1297-303.

51. DePristo MA, Banks E, Poplin R, Garimella KV, Maguire JR, Hartl C , et al. A framework for variation discovery and genotyping using next-generation DNA sequencing data. Nat Genet 2011; 43: 491-8.

52. Van der Auwera GA, Carneiro MO, Hartl C, Poplin R, Del Angel G, Levy-Moonshine A , et al. From FastQ data to high confidence variant calls: the Genome Analysis Toolkit best practices pipeline. Curr Protoc Bioinformatics 2013; 43: 11 0 1- 0 33.

53. Wang K, Li M,Hakonarson H. ANNOVAR: functional annotation of genetic variants from high-throughput sequencing data. Nucleic acids research 2010; 38: e164.

54. Kandoth C. mskcc/vcf2maf: vcf2maf. .

55. McLaren W, Gil L, Hunt SE, Riat HS, Ritchie GR, Thormann A , et al. The Ensembl Variant Effect Predictor. Genome Biol 2016; 17: 122.

56. Mayakonda A, Lin DC, Assenov Y, Plass C,Koeffler HP. Maftools: efficient and comprehensive analysis of somatic variants in cancer. Genome research 2018; 28: 1747-56.

57. Lai Z, Markovets A, Ahdesmaki M, Chapman B, Hofmann O, McEwen R , et al. VarDict: a novel and versatile variant caller for next-generation sequencing in cancer research. Nucleic acids research 2016; 44: e108.

58. Chen X, Schulz-Trieglaff O, Shaw R, Barnes B, Schlesinger F, Kallberg M , et al. Manta: rapid detection of structural variants and indels for germline and cancer sequencing applications. Bioinformatics 2016; 32: 1220-2.

59. Cristiano Oliveira, Thomas Wolf: CNVPanelizer: Reliable CNV detection in targeted sequencing applications. R package version 1.22.0. (2020). <https://bioconductor.org/packages/release/bioc/html/CNVPanelizer.html>.

60. MSIsensor-pro: Fast, Accurate, and Matched-normal-sample-free Detection of Microsatellite Instability. <https://doi.org/10.1016/j.gpb.2020.02.001>.

61. A program for annotating and predicting the effects of single nucleotide polymorphisms, SnpEff. doi: 10.4161/fly.19695.
