# Supplementary material for: Bcl-xL Is a Key Mediator of Apoptosis Following KRASG12C Inhibition in KRASG12C-mutant Colorectal Cancer
Source: Mol Cancer Ther. 2022 Oct 22;22(1):135–49. doi: 10.1158/1535-7163.MCT-22-0301 (PMC9808374; doi:10.1158/1535-7163.MCT-22-0301)
Supplement: Supplementary Figures S1-S6 — Supplementary figures [file mct-22-0301_supplementary_figures_s1-s6_suppsf1-sf6.pdf]

A.

KRAS^{G12C} CRC cell lines	KRAS exon 2 Sanger sequencing	Additional characteristics	AZ'1569- IC_{50} ± SD (μM)
RW7213	p.G12C homo	TP53 p.L257R, MSS	0.26 ± 0.05
C106	p.G12C hetero	TP53 p.T125M, MSS	0.43 ± 0.08
SW1463	p.G12C homo	TP53 p.R248Q, MSS	1.39 ± 0.87
SW837	p.G12C hetero	TP53 p.R248W, MSS	1.54 ± 0.47
LIM2099	p.G12C homo	MSS	1.72 ± 0.37
SNU1411	p.G12C homo	TP53 p.S94*, R72P, MSS	2.96 ± 0.54
V481	p.G12C hetero	PTEN null, PIK3CA Q546P, MSI-H	3.4 ± 1.7
***KRAS*^{G13D} CRC cell line**			
HCT116			6.8 ± 2.6

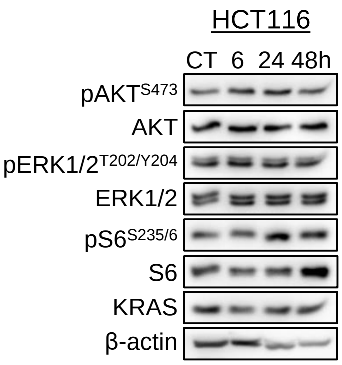

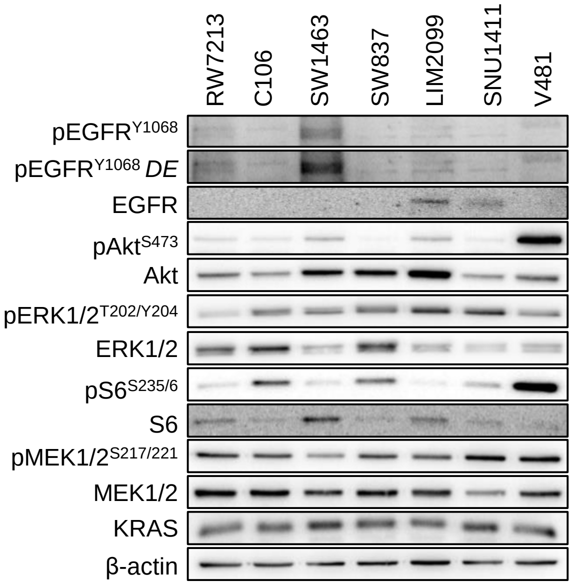

B.

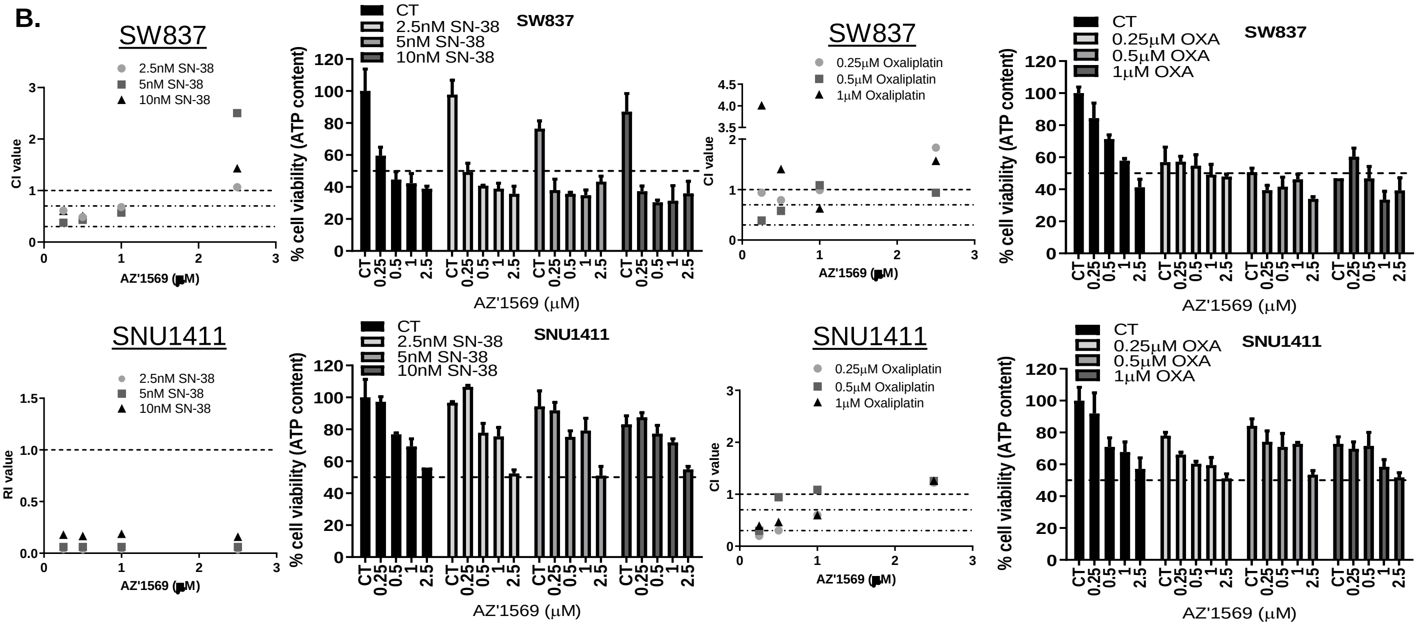

C.

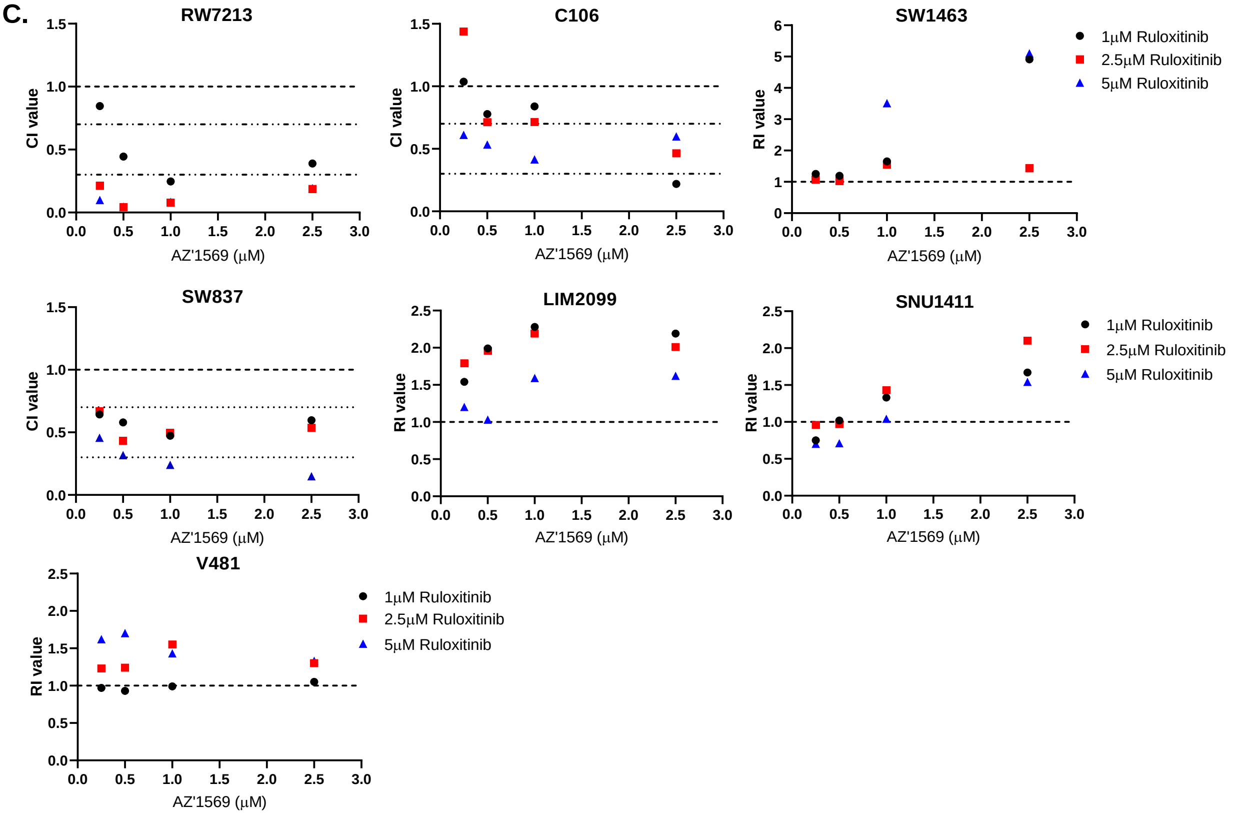

D.

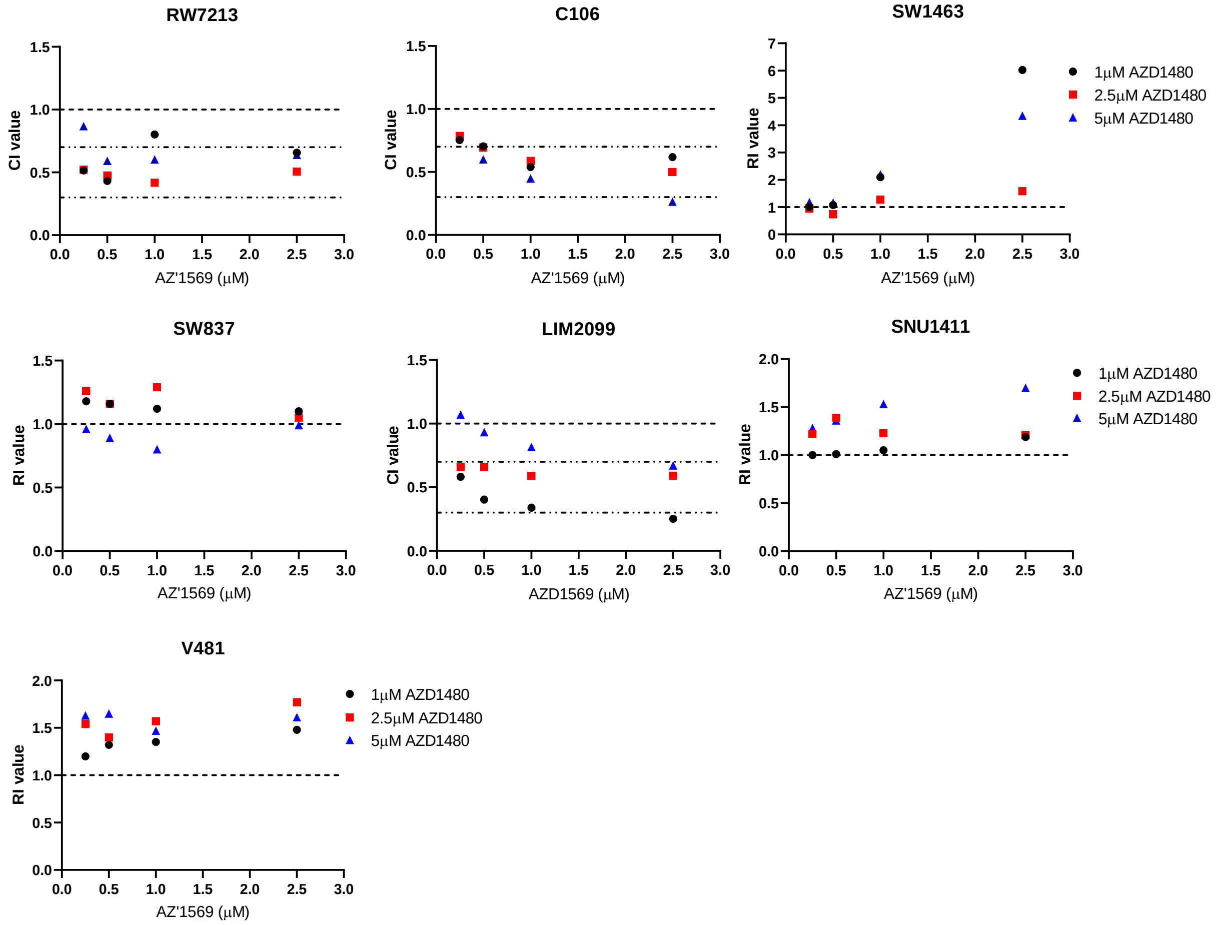
RW7213
C106
SW1463
SW837
LIM2099
SNU1411
V481
CI value
RI value
AZ'1569 (µM)
AZD1569 (µM)
1µM AZD1480
2.5µM AZD1480
5µM AZD1480

E.

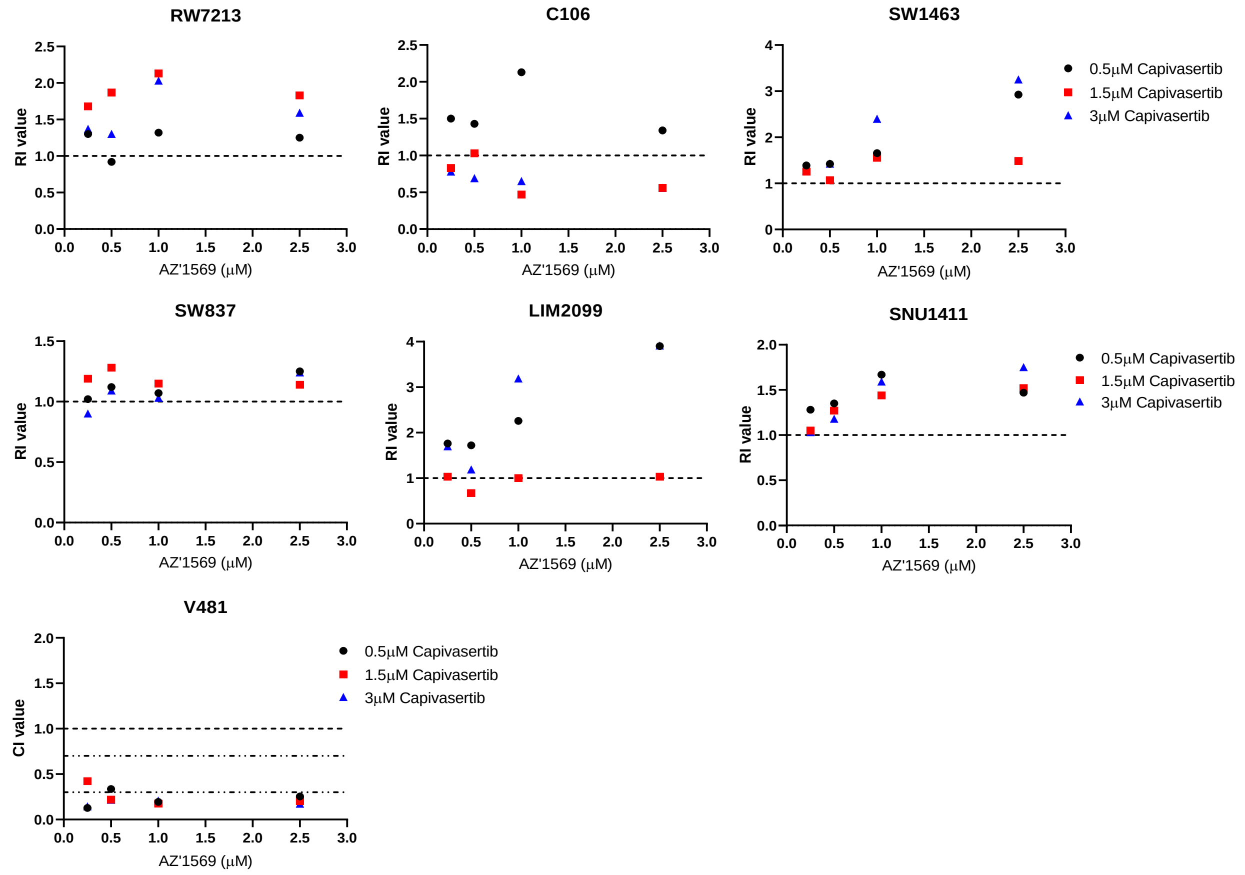
RW7213
C106
SW1463
SW837
LIM2099
SNU1411
V481
RI value
CI value
AZ'1569 (µM)
0.5µM Capivasertib
1.5µM Capivasertib
3µM Capivasertib

F.

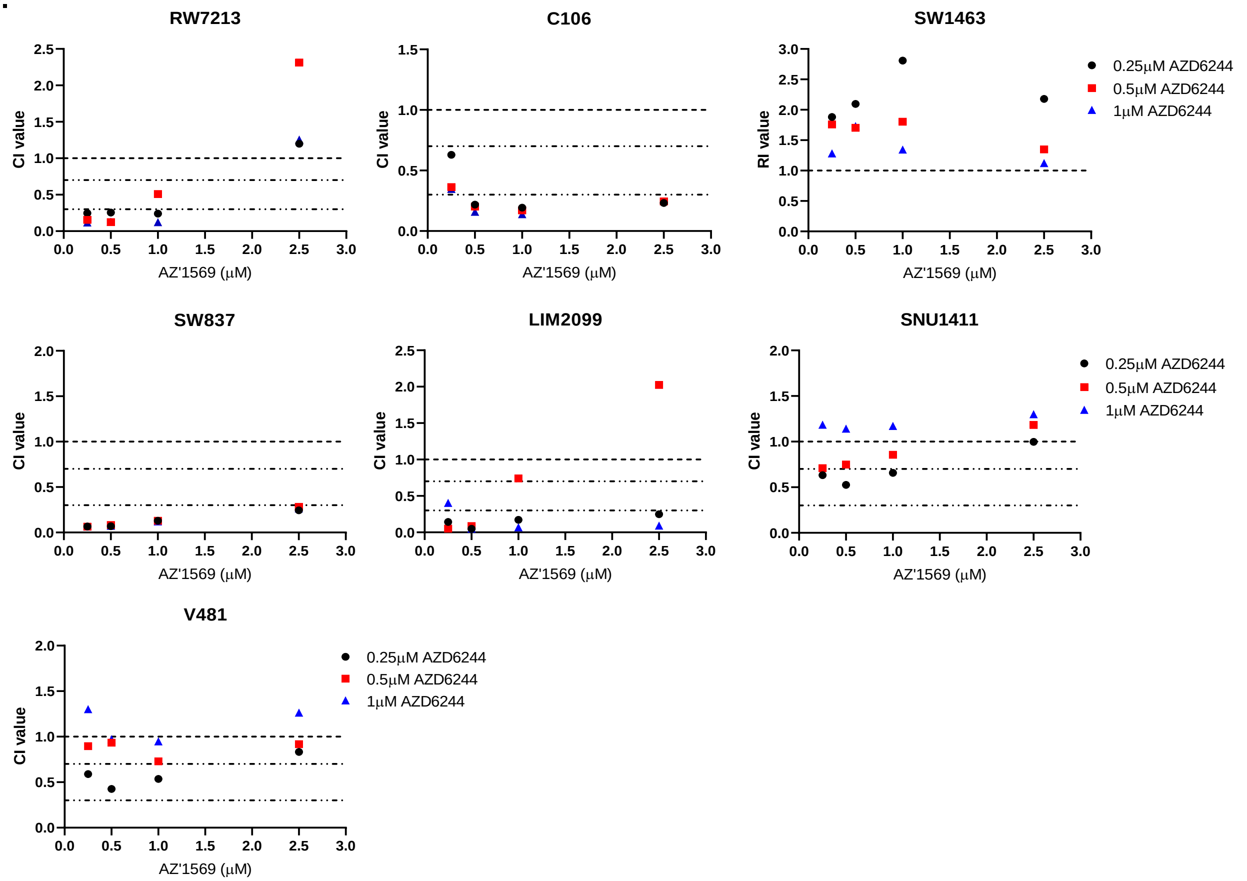
RW7213
C106
SW1463
SW837
LIM2099
SNU1411
V481
CI value
RI value
AZ'1569 (μM)
0.25μM AZD6244
0.5μM AZD6244
1μM AZD6244

G.

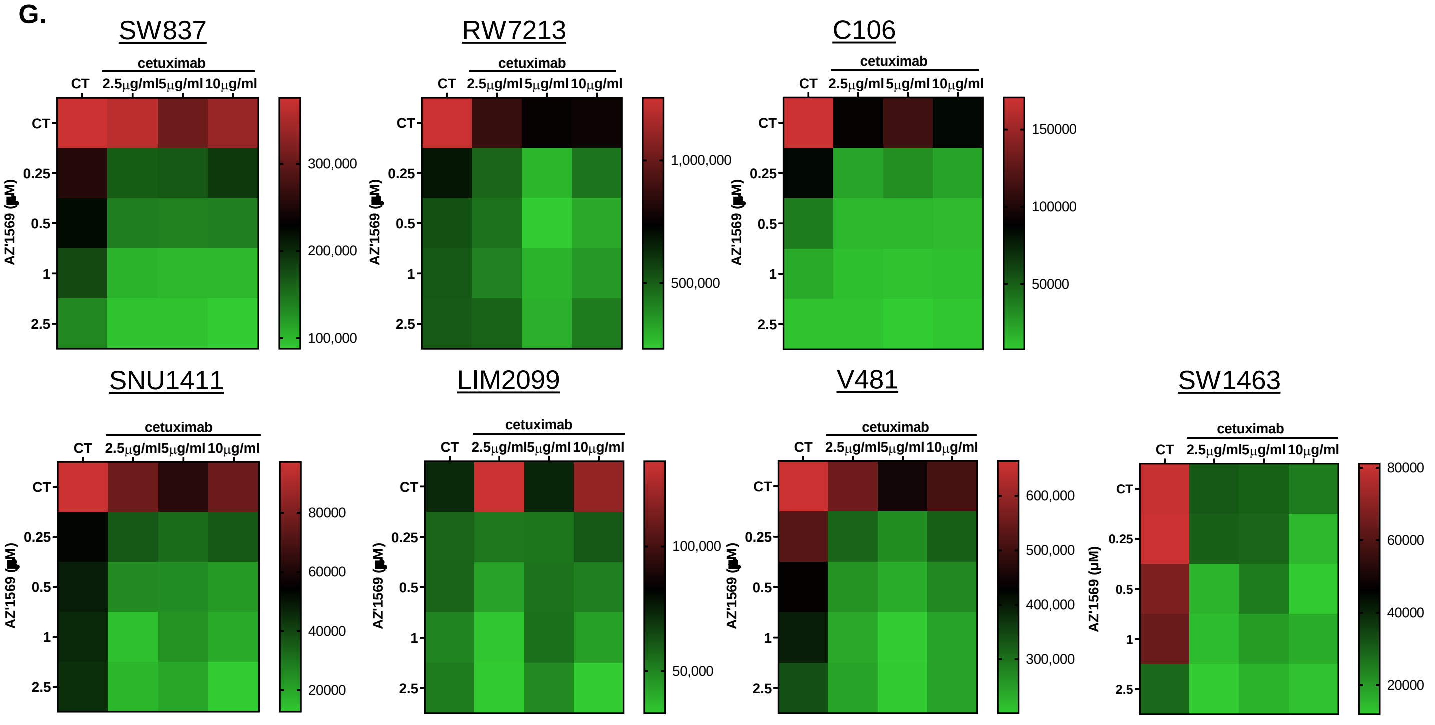
SW837
RW7213
C106
SNU1411
LIM2099
V481
SW1463
cetuximab
CT
2.5μg/ml
5μg/ml
10μg/ml
AZ'1569 (μM)
0.25
0.5
1
2.5
300,000
200,000
100,000
1,000,000
500,000
150000
100000
50000
80000
60000
40000
20000
100,000
50,000
600,000
500,000
400,000
300,000

H.

Cell line	AZD6244	AZD1480	Ruloxitinib	Capivasertib	Cetuximab
**RW7213**	Moderate – high synergy	Slight-Moderate synergy	Moderate-high synergy	Additive – moderate synergy	High synergy
**C106**	Moderate – high synergy	Slight-Moderate synergy	Slight-Moderate synergy	Antagonistic - Additive	Moderate synergy
**SNU1411**	Additive – slight synergy	Additive – slight synergy	Additive – slight synergy	Additive – slight synergy	High synergy
**LIM2099**	Moderate – high synergy	Moderate synergy	Slight - Moderate synergy	Additive – moderate synergy	High synergy
**SW837**	High synergy	Additive	Moderate synergy	Additive	Moderate-high synergy
**SW1463**	Additive – moderate synergy	Additive – moderate synergy	Additive – moderate synergy	Additive – moderate synergy	Moderate synergy
**V481**	Additive - Slight synergy	Additive - Slight synergy	Additive - Slight synergy	High synergy	Moderate-high synergy

Supplementary Figure 1. Response of *KRAS*^{G12C}MT CRC cells to KRAS^{G12C} inhibitor AZ'1569 alone, combined with standard-of-care chemotherapies, cetuximab or inhibitors of the KRAS downstream effectors. A. Left: KRAS exon 2 Sanger sequencing results for the panel of 7 *KRAS*^{G12C} MT CRC cells. Homozygous (homo), heterozygous (het). Additional mutational changes and MSI (Microsatellite instability) status is also presented (refs. 20-23). CRC cells were treated with AZ'1569 for 120h and cell viability determined using CellTiter-Glo® (CTG) assay. IC_{50} values were calculated using Prism software package. Mean of 3 independent experiments with Standard Deviation is presented in the table. (MSI-H = Microsatellite instability High; MSS = Microsatellite Stable. **Middle:** HCT116 *KRAS^{G13D}* cells were treated with 1µM AZ'1569 for 0, 6, 24 and 48h. WB was used to evaluate the protein expression of phosphorylated and total AKT, ERK1/2, S6 ribosomal protein, KRAS and β-actin. **Right:** Basal expression levels of proteins within the EGFR/KRAS signalling axis, as determined by Western blotting (WB). DE=darker exposure. **B.** CTG assays in CRC cells treated with no drug (control), SN-38 or oxaliplatin, AZ'1569, SN-38 or oxaliplatin in combination with AZ'1569 for 72h. CI values were calculated using the method of Chou and Talalay. CI values >1, <1, and equal to 1 indicate antagonism, synergy and additive effects for drug combinations, respectively. Dashed lines indicate CI values of 0.3, 0.7 and 1. RI values were used where a compound had little/no effect on cell viability. RI values >1, <1, and equal to 1 indicate synergy, antagonism and additive effects for drug combinations, respectively. Absolute cell viability for different combinations is also shown. Dashed line indicates 50% cell viability. Representative results of at least three experiments are shown. **C, D, E, F, G**. *KRAS*^{G12C}MT CRC cells were treated with AZ'1569 alone or combined with Ruloxitinib (**C**), AZD1480 (**D**), Capivasertib (**E**), AZD6244 (**F**) or cetuximab (**G**) for 120h and cell viability assessed using the CTG assay and CI/RI values were calculated. Heatmaps represent the absolute reduction in cell viability for combination of AZ'1569 with cetuximab. Representative results of at least three experiments are shown. **H.** Table summarising the nature of interaction between AZ'1569 and the different targeted drugs in the panel of *KRAS*^{G12C}MT CRC cells.

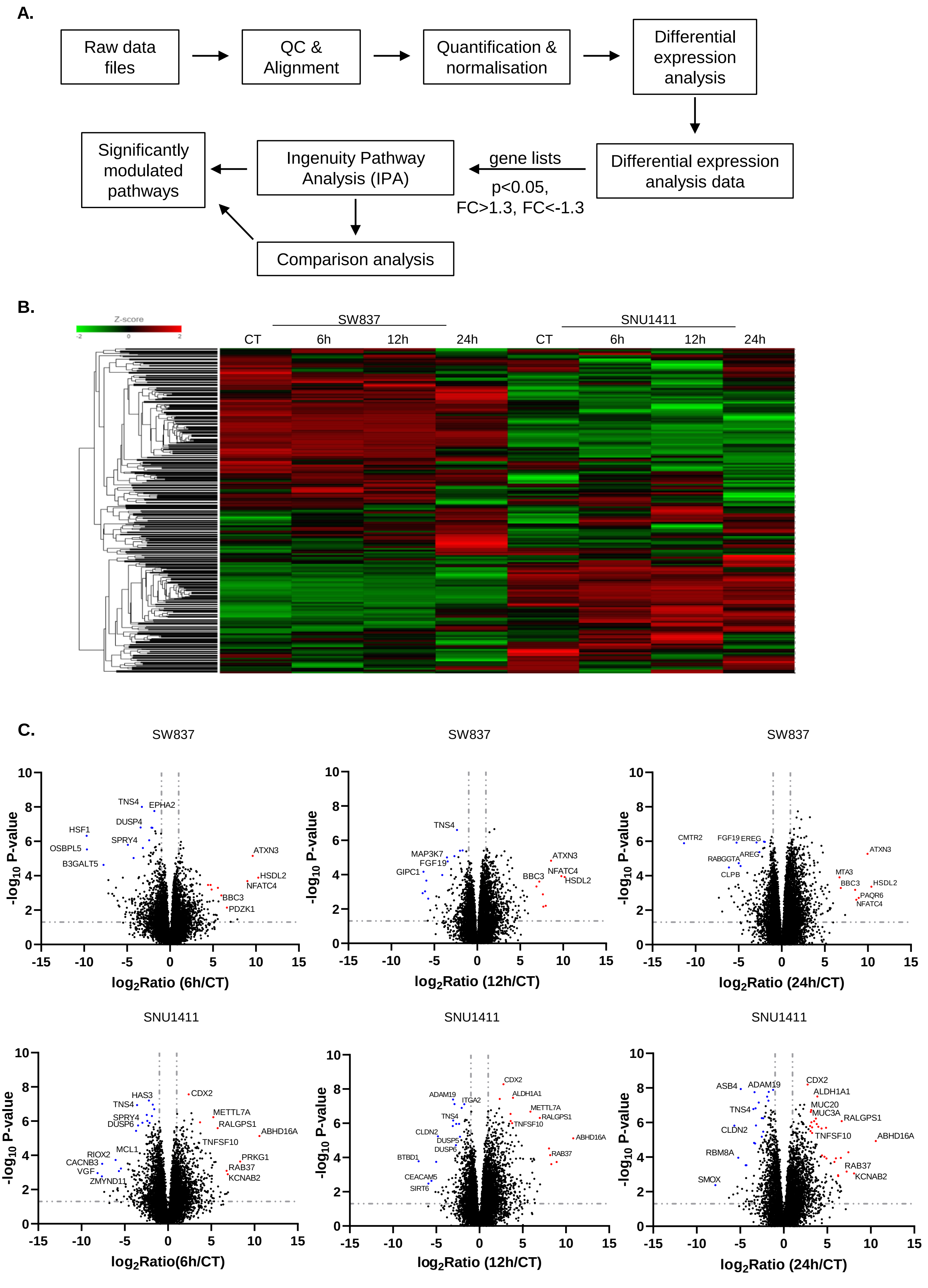
A.
Raw data files
QC & Alignment
Quantification & normalisation
Differential expression analysis
Differential expression analysis data
gene lists
p<0.05, FC>1.3, FC<-1.3
Ingenuity Pathway Analysis (IPA)
Comparison analysis
Significantly modulated pathways
B.
Z-score
-2
0
2
SW837
SNU1411
CT
6h
12h
24h
CT
6h
12h
24h
C.
SW837
TNS4
EPHA2
DUSP4
HSF1
SPRY4
OSBPL5
B3GALT5
ATXN3
HSDL2
NFATC4
BBC3
PDZK1
log2Ratio (6h/CT)
-log10 P-value
SW837
TNS4
MAP3K7
FGF19
GIPC1
ATXN3
BBC3
NFATC4
HSDL2
log2Ratio (12h/CT)
SW837
CMTR2
FGF19
EREG
RABGGTA
AREG
CLPB
ATXN3
MTA3
BBC3
HSDL2
PAQR6
NFATC4
log2Ratio (24h/CT)
SNU1411
HAS3
TNS4
SPRY4
DUSP6
RIOX2
MCL1
CACNB3
VGF
ZMYND11
CDX2
METTL7A
RALGPS1
ABHD16A
TNFSF10
PRKG1
RAB37
KCNAB2
log2Ratio(6h/CT)
SNU1411
ADAM19
ITGA2
TNS4
CLDN2
DUSP5
DUSP6
BTBD1
CEACAM5
SIRT6
CDX2
ALDH1A1
METTL7A
RALGPS1
TNFSF10
ABHD16A
RAB37
log2Ratio (12h/CT)
SNU1411
ASB4
ADAM19
TNS4
CLDN2
RBM8A
SMOX
CDX2
ALDH1A1
MUC20
MUC3A
RALGPS1
TNFSF10
ABHD16A
RAB37
KCNAB2
log2Ratio (24h/CT)

D.

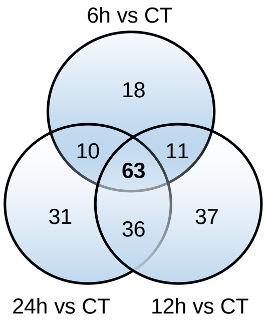

	SW837 -log_{10}pvalue			SNU1411 -log_{10}pvalue		
**Pathway**	**6h**	**12h**	**24h**	**6h**	**12h**	**24h**
Molecular Mechanisms of Cancer	8.066559	9.160485	10.57428	10.04854	14.2449	6.75044
EIF2 Signaling	5.86876	7.00092	15.20365	6.62961	3.546702	1.897453
Death Receptor Signaling	8.000209	6.777285	4.362492	3.503648	4.676862	1.718967
mTOR Signaling	4.451432	3.765041	7.519563	5.400755	5.094452	4.531991
Cell Cycle: G1/S Checkpoint Regulation	5.620854	8.242366	7.647154	3.963368	5.37469	2.854804
ERK/MAPK Signaling	6.308891	3.00745	3.190258	3.053669	5.95694	7.065112
Germ Cell-Sertoli Cell Junction Signaling	4.627643	3.056515	3.808487	4.169748	5.01844	7.698286
Sirtuin Signaling Pathway	2.141829	6.681894	15.37388	6.373353	9.461571	12.54052
HIPPO signaling	4.517063	4.73105	6.743086	3.694088	3.779193	5.108474
Glioblastoma Multiforme Signaling	5.055339	2.185464	4.107408	3.106733	3.141882	2.303813
Neuregulin Signaling	4.347798	2.842146	2.174521	3.796415	4.082892	6.719828
Chronic Myeloid Leukemia Signaling	5.008688	4.347044	4.183806	2.848538	7.074057	4.845524
Pancreatic Adenocarcinoma Signaling	4.45597	3.411957	7.827764	3.377138	5.33906	2.276024
HGF Signaling	6.046239	3.243559	3.235769	1.773783	2.681906	3.605607
Aryl Hydrocarbon Receptor Signaling	3.425917	5.63355	4.986279	4.367558	6.098434	5.574729
Cyclins and Cell Cycle Regulation	4.461027	6.785328	6.56417	2.922904	6.117661	4.348443
Senescence Pathway	3.877711	7.316643	5.66488	3.343923	11.18359	9.807846
Regulation of eIF4 and p70S6K Signaling	3.53107	6.509098	9.571548	3.628492	6.061464	4.833019
p53 Signaling	4.166847	5.333847	4.889511	2.580736	3.86517	5.496942
Integrin Signaling	3.976514	1.6583	1.906902	2.732539	4.853825	10.56651
PI3K/AKT Signaling	2.841681	2.816287	2.321825	3.596005	6.352229	5.763325
AMPK Signaling	2.145715	5.839138	4.375414	4.210971	7.001451	6.317038
Apoptosis Signaling	4.079121	2.605405	1.922188	2.210349	2.754154	2.480502
Necroptosis Signaling Pathway	2.924407	3.725963	4.962115	3.332631	4.994035	3.056993
Mouse Embryonic Stem Cell Pluripotency	3.363275	1.544675	3.186304	2.848538	3.037925	3.050145
Glioma Signaling	4.36974	2.405081	3.044134	1.82709	4.817612	3.055761
Hepatic Fibrosis Signaling Pathway	3.32774	2.692935	2.223829	2.835887	3.463347	2.884307
Huntington's Disease Signaling	3.852445	4.171777	3.696712	2.098702	3.233941	2.990968
RAR Activation	1.810508	3.19748	3.113958	4.080867	8.03045	4.375325
Superpathway of Inositol Phosphate Compounds	2.316563	1.369722	3.263612	3.547439	7.212149	4.925232
Telomerase Signaling	3.81739	3.246768	2.498456	1.995155	3.663634	4.375888
PDGF Signaling	3.545288	1.52025	1.768099	2.193894	3.295954	2.074923
Colorectal Cancer Metastasis Signaling	3.107716	1.5551	2.278083	2.625719	3.440302	2.193115
Protein Kinase A Signaling	2.718927	5.403933	3.54176	2.984975	5.001706	6.027761
Epithelial Adherens Junction Signaling	3.516023	4.111612	2.915876	2.060211	3.542839	8.081199
Wnt/β-catenin Signaling	2.670487	2.009373	2.252591	2.900464	3.116873	2.504296
Adipogenesis pathway	2.41708	2.746657	3.500025	3.110097	4.480369	4.388986
Regulation of the Epithelial-Mesenchymal Transition Pathway	2.823253	2.157012	2.408112	2.620357	2.892655	1.729734
ErbB2-ErbB3 Signaling	2.865778	2.829327	1.533991	2.507073	2.93823	2.349024
Estrogen Receptor Signaling	2.481793	4.080669	3.6723	2.887593	6.565692	4.545054
PTEN Signaling	1.993212	2.451554	2.627861	3.374309	4.942862	5.239909
FAK Signaling	2.847932	1.749024	2.006656	2.484043	3.452761	4.625694
ILK Signaling	3.768393	2.468829	3.672179	1.48336	4.337454	3.584841
ErbB Signaling	3.291081	1.558632	1.614358	1.960085	2.557678	3.24187
Hereditary Breast Cancer Signaling	2.973333	4.460687	7.270296	2.198599	4.932814	5.170671
ERK5 Signaling	3.093571	4.124698	1.223689	1.645719	5.567041	5.205068
Role of CHK Proteins in Cell Cycle Checkpoint Control	3.177071	3.77546	7.299524	1.451807	6.128736	2.466845
14-3-3-mediated Signaling	3.120791	2.944511	2.542891	1.462586	1.873977	3.737028
Renal Cell Carcinoma Signaling	2.839561	1.621615	2.317974	1.710091	2.836165	4.482347
Cell Cycle Regulation by BTG Family Proteins	2.400441	4.973676	4.107646	2.070882	5.593748	2.497677
RhoA Signaling	1.39444	2.375151	3.172541	2.965708	2.907493	7.296667
Endometrial Cancer Signaling	2.465495	3.388967	1.452742	1.867319	3.12552	2.890514
Sertoli Cell-Sertoli Cell Junction Signaling	2.382306	4.05599	5.261775	1.859315	5.705946	7.211127
B Cell Receptor Signaling	2.382306	3.231215	1.727015	1.666659	3.085727	1.355699
PFKFB4 Signaling Pathway	1.607917	2.893836	2.023514	2.382936	4.261462	1.817483
IL-15 Production	1.470977	2.787775	3.676793	2.238406	3.06506	1.749397
Tight Junction Signaling	1.905518	2.255402	2.383873	1.765485	6.736873	4.465814
Non-Small Cell Lung Cancer Signaling	2.243175	2.440347	1.611819	1.31747	3.19904	2.257231
Role of BRCA1 in DNA Damage Response	1.805638	1.368366	7.307542	1.710091	3.969201	1.974017
Axonal Guidance Signaling	1.772682	2.089048	2.595897	1.635474	5.259315	4.79811
Role of PKR in Interferon Induction and Antiviral Response	1.889369	3.403232	3.201751	1.480343	3.401816	2.235164
Inhibition of ARE-Mediated mRNA Degradation Pathway	1.43228	3.011667	2.467691	1.927513	5.004266	3.244749
Unfolded protein response	1.315897	2.586719	3.28722	1.843496	1.420228	2.578968
Pyridoxal 5'-phosphate Salvage Pathway	1.426833	2.454636	3.934055	1.517237	1.602019	2.703549

E.

Pathway	Genes
Death receptor signalling	*ACIN1, ACTB, ACTG1, APAF1, ARHGDIB, BID, BIRC2, CASP10, CASP3, CASP6, CASP7, CASP8, CFLAR, DAXX, DFFB, HSPB1, IKBKG, LIMK1, MAP3K5, MAP4K4, MAPK8, NAIP, NFKBIA, NFKBID, PARP1, PARP10, PARP12, PARP14, PARP2, PARP4, PARP6, PARP8, PARP9, SPTAN1, TIPARP, TNFRSF1A, TNFRSF1B, TNFRSF21, TNFRSF25, TNFSF15, XIAP*
Apoptosis Signalling	*ACIN1, APAF1, BAD, BAK1, BCL2L1, BCL2L10, BIRC2, BIRC6, CAPN5, CAPNS1, CASP10, CASP7, CASP8, CDK1, DFFB, ENDOG, HTRA2, IKBKB, IKBKG, KRAS, MAP2K7, MAP3K5, MAP4K4, MAPK3, MAPK8, MCL1, NFKBIA, NFKBIB, PARP1, PLCG1, PLCG2, PRKCA, PRKCQ, RALB, RAP2B, RASD2, RRAS, SPTAN1, TNFRSF1A,TNFRSF1B, TP53, XIAP*
Necroptosis signalling	*AXL,BIRC2,BIRC3,CAMK2D,CAPN1,CAPN2,CAPN5,CAPNS1,CASP10,CASP8,CFLAR,CHUK,CYB B,CYLD,DAPK1,DNM1L,FKBP1A,GLUL,IKBKB,IKBKG,IRF3,IRF9,JAK1,JMJD7-PLA2G4B, MAP3K7, MERTK, PAM16, PLA2G10, PLA2G12A, PLA2G4B, PPP3CA, PPP3CB, RBCK1, RBL1, SHARPIN, SLC25A3, SLC25A4, SLC25A6, TAB2, TIMM17B, TIMM8B, TNFRSF1A, TNFRSF1B,TNFSF10,TNIP1,TOMM40,TOMM40L,TOMM7,TOMM70, TP53,TRADD,TSPO,TYRO3,UBC,VDAC2,VDAC3*
p53 signalling	*AKT1,AKT2,APAF1,ATM,BCL2L1,BIRC5,BRCA1,CCNK,CDK2,CHEK1, CHEK2, COQ8A, CSNK1D, CTNNB1, DRAM1, E2F1, GADD45B, GNL3,HIF1A,HIPK2,KAT2B,MAPK14,MDM4,PIK3C3, PIK3CA, IK3CB,PIK3R1,PIK3R2,PMAIP1,PML,PRKDC,PTEN,SCO2, SERPINB5, ERPINE2,SFN,THBS1,TP53,TP53INP1,TP73,TRIM29*

Supplementary Figure 2. KRAS^{G12C} inhibition rewires the signalling network of *KRAS*^{G12C}MT CRC cells. A. Overview of the bioinformatics analysis of *in vitro* data in SW837 and SNU1411 cells to comprehensively map adaptive signalling following AZ’1569 inhibition. Generated FASTQ files were analysed using a workflow on Partek® Flow software, v10.0. Post-alignment QC and quantification of aligned reads to an annotation model (Partek E/M, default settings; min reads=10) was performed. Differential expression analysis was performed using the GSA (gene specific analysis) tool. A cut-off threshold of fold-change (FC) >1.3 or <-1.3, and p-value<0.05 was applied to gene lists. The resulting gene lists were imported into Ingenuity Pathway Analysis (IPA) software (Qiagen, UK) to identify significantly enriched pathways for each time-point following AZ’1569 treatment in both cell lines. Comparison analysis in IPA was used to compare significantly enriched ($-\log_{10}$ p-value >1.3) pathways across both cell lines. **B**. Two-dimensional hierarchical clustering analysis of genes that were induced or repressed following treatment of *KRAS*^{G12C}MT SW837 and SNU1411 cells with AZ’1569 for 6h, 12h and 24h compared to the untreated cells. **C**. Volcano plots show the up- and downregulated genes following AZ’1569 treatment in SW837 and SNU1411 cells at the indicated time-points. Dashed lines on the x and y-axis indicate $\log_2$ratio of 1/-1, and $-\log_{10}$ p-value=1.3, respectively. **D. Left:** Venn diagram of the up- and down regulated pathways at each of the time points in both SW837 and SNU1411 cells. CT = control. **Right**: Results of the IPA pathway analysis. List of the common 63 pathways altered following AZ’1569 treatment in both SW837 and SNU1411 cells at each of the time points. **E.** Table representing the significantly enriched pathways with gene sets in cell death-related signalling pathways.

A.

CatalogNumber	Gene Symbol	GeneId	Description
L-015375-00	ATG3	64422	ATG3 ON-TARGETplus SMARTpool - Human
L-004374-00	ATG5	9474	ATG5 ON-TARGETplus SMARTpool - Human
L-020112-00	ATG7	10533	ATG7 ON-TARGETplus SMARTpool - Human
L-003870-00	BAD	572	BAD ON-TARGETplus SMARTpool - Human
L-003305-00	BAK1	578	BAK1 ON-TARGETplus SMARTpool - Human
L-003308-01	BAX	581	BAX ON-TARGETplus SMARTpool - Human
L-004380-00	BBC3	27113	BBC3 ON-TARGETplus SMARTpool - Human
L-003306-00	BCL2A1	597	BCL2A1 ON-TARGETplus SMARTpool - Human
L-004383-00	BCL2L11	10018	BCL2L11 ON-TARGETplus SMARTpool - Human
L-003458-00	BCL2L1	598	BCL2L1 ON-TARGETplus SMARTpool - Human
L-004384-00	BCL2L2	599	BCL2L2 ON-TARGETplus SMARTpool - Human
L-003307-00	BCL2	596	BCL2 ON-TARGETplus SMARTpool - Human
L-010552-00	BECN1	8678	BECN1 ON-TARGETplus SMARTpool - Human
L-004387-00	BID	637	BID ON-TARGETplus SMARTpool - Human
L-004388-00	BIK	638	BIK ON-TARGETplus SMARTpool - Human
L-004390-00	BIRC2	329	BIRC2 ON-TARGETplus SMARTpool - Human
L-004099-00	BIRC3	330	BIRC3 ON-TARGETplus SMARTpool - Human
L-004393-00	BMF	90427	BMF ON-TARGETplus SMARTpool - Human
L-004394-00	BOK	666	BOK ON-TARGETplus SMARTpool - Human
L-004402-00	CASP10	843	CASP10 ON-TARGETplus SMARTpool - Human
L-004401-00	CASP1	834	CASP1 ON-TARGETplus SMARTpool - Human
L-003465-00	CASP2	835	CASP2 ON-TARGETplus SMARTpool - Human
L-004307-00	CASP3	836	CASP3 ON-TARGETplus SMARTpool - Human
L-004406-00	CASP6	839	CASP6 ON-TARGETplus SMARTpool - Human
L-004407-00	CASP7	840	CASP7 ON-TARGETplus SMARTpool - Human
L-003466-00	CASP8	841	CASP8 ON-TARGETplus SMARTpool - Human
L-003309-00	CASP9	842	CASP9 ON-TARGETplus SMARTpool - Human
L-003772-00	CFLAR	8837	CFLAR ON-TARGETplus SMARTpool - Human
L-003800-00	FADD	8772	FADD ON-TARGETplus SMARTpool - Human
L-003776-00	FAS	355	FAS ON-TARGETplus SMARTpool - Human
L-008216-00	HRK	8739	HRK ON-TARGETplus SMARTpool - Human
L-003585-00	MAP3K9	4293	MAP3K9 ON-TARGETplus SMARTpool - Human
L-004501-00	MCL1	4170	MCL1 ON-TARGETplus SMARTpool - Human
L-005275-00	PMAIP1	5366	PMAIP1 ON-TARGETplus SMARTpool - Human
L-003533-00	RELA	5970	RELA ON-TARGETplus SMARTpool - Human
L-004445-00	RIPK1	8737	RIPK1 ON-TARGETplus SMARTpool - Human
L-003534-00	RIPK3	11035	RIPK3 ON-TARGETplus SMARTpool - Human
L-003544-00	STAT3	6774	STAT3 ON-TARGETplus SMARTpool - Human
L-008090-00	TNFRSF10A	8797	TNFRSF10A ON-TARGETplus SMARTpool - Human
L-004448-00	TNFRSF10B	8795	TNFRSF10B ON-TARGETplus SMARTpool - Human
L-003329-00	TP53	7157	TP53 ON-TARGETplus SMARTpool - Human
L-004098-00	XIAP	331	XIAP ON-TARGETplus SMARTpool - Human

B.

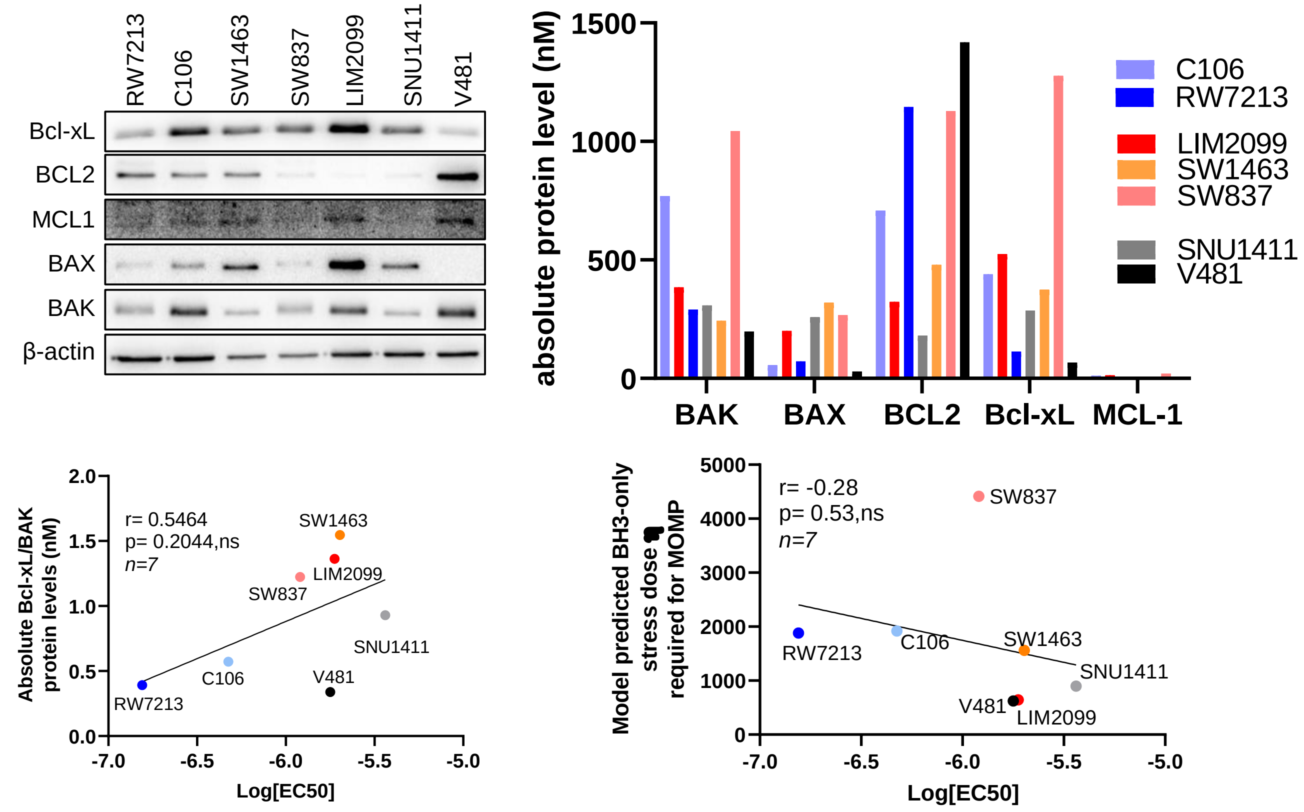

C.

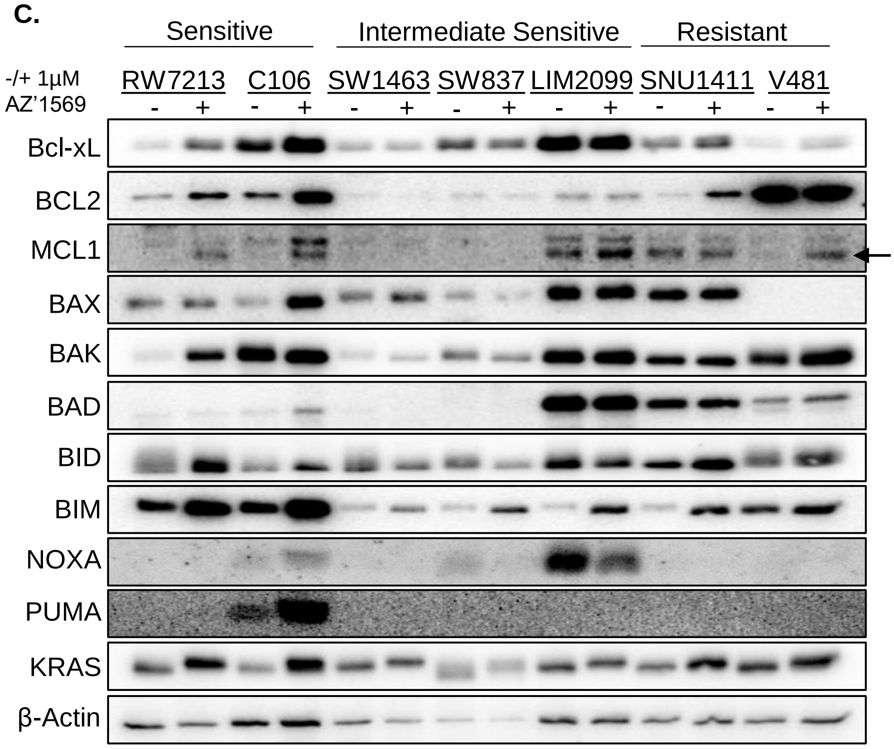

D.

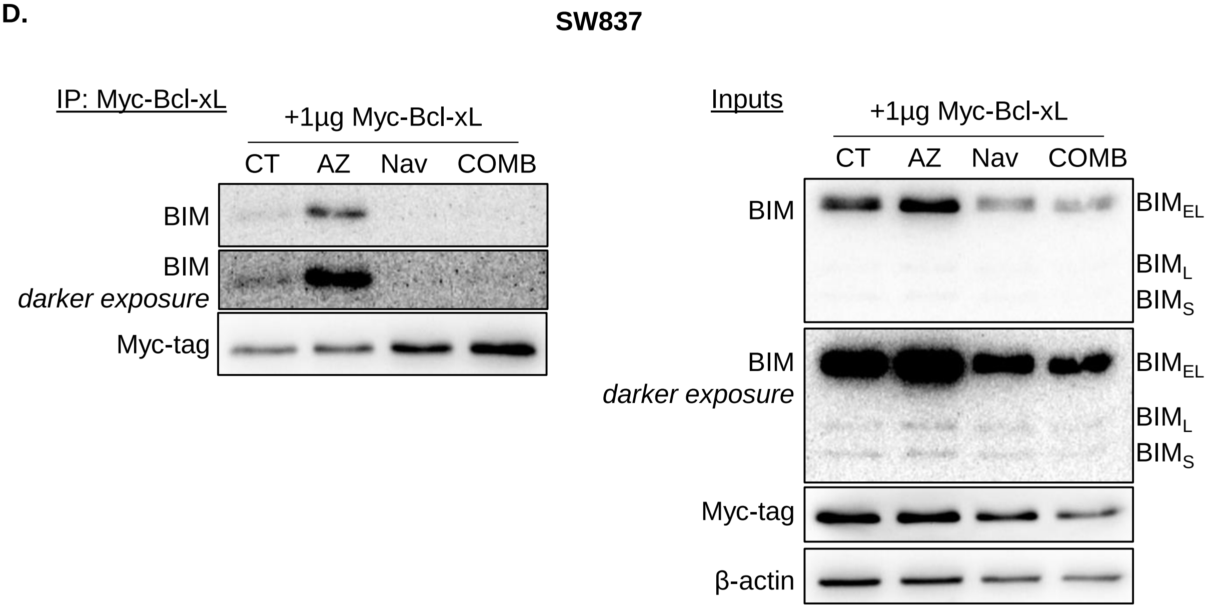

Supplementary figure 3. Bcl-xL is a major escape pathway following KRAS^{G12C} inhibition in *KRAS*^{G12C}MT CRC cells. A. Details of the ON-Targetplus siRNA library (Dharmacon) used to identify common targets of intrinsic resistance to KRAS^{G12C} inhibition in SW837 and SNU1411 cells. **B. Top Left:** Basal expression levels of Bcl-xL, BCL2, MCL1, BAX and BAK, as determined by WB. **Top right**: Absolute protein levels of the BCL2 family proteins BAK, BAX, BCL2, Bcl-xL and MCL1. Protein levels obtained from quantitative WB were first normalised to β-actin and eventually normalised to HCT116 cells to obtain absolute protein levels in nM. Protein levels in HCT116 cells were previously quantified as described in Lindner *et al.* 2013 (Ref. 16). **Bottom left:** Pearson correlation of absolute protein Bcl-xL/BAK ratio plotted against Log[EC_{50}] AZ'1569 in the panel of *KRAS*^{G12C}MT cell lines. **Bottom right:** Pearson correlation of the model predicted stress dose required to induce MOMP plotted against Log[EC_{50}] AZ'1569 in the panel of *KRAS*^{G12C} MT CRC cell lines. **C.** *KRAS*^{G12C}MT cell lines were treated with 1µM AZ'1569 for 48h. Expression of BCL2 family members was determined by WB. **D.** SW837 cells were transfected with 1µg Myc-tagged Bcl-xL for 24 hours, followed by treatment with 1µM AZ'1569 (AZ), 1µM Navitoclax (Nav), or combination (COMB) for a further 24 hours. Cells were harvested and lysed and Myc-tagged Bcl-xL was immunoprecipitated. WB was used to determine the expression of Myc-tag, Bim and β-actin. Data is representative of three independent experiments.

A.

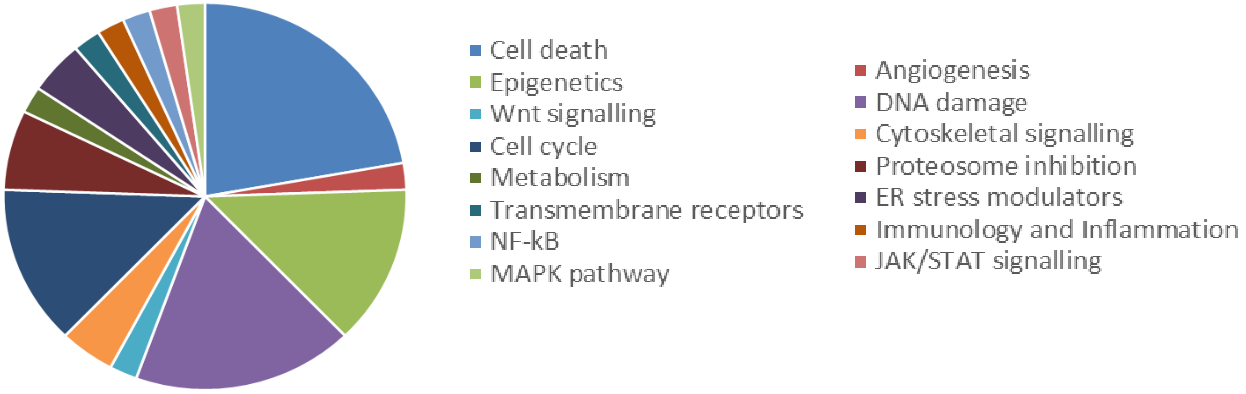

Name	Pathway	Target/Process
ABT-737	Apoptosis/cell death	Bcl-2, Bcl-xL, Bcl-w, ,Autophagy
Saracatinib (AZD0530)	Angiogenesis/ Src inhibitor	Src
Vorinostat (SAHA, MK0683)	Epigenetics	Autophagy,HDAC
Entinostat (MS-275)	Epigenetics	HDAC
Olaparib (AZD2281, Ku-0059436)	DNA damage	PARP
Nutlin-3	MDM2 antagonist/ p53/apoptosis	E3 Ligase ,Mdm2
Vismodegib (GDC-0449)	Wnt signalling pathway	Hedgehog/Smoothened
Alisertib (MLN8237)	Cell cycle	Aurora Kinase
Barasertib (AZD1152-HQPA)	Cell cycle	Aurora Kinase
Roscovitine (Seliciclib,CYC202)	Cell cycle	CDK
Ganetespib (STA-9090)	cytoskeletal signalling/ HSP90 inhibitor	HSP (e.g. HSP90)
BIBR 1532	DNA damage	Telomerase
Epothilone A	cytoskeletal signalling	Microtubule Associated
AZD7762	cell cycle	Chk
Ixazomib (MLN2238)	proteosome inhibitor	Proteasome
Degrasyn (WP1130)	proteosome inhibitor	Bcr-Abl,DUB
Rosiglitazone	Metabolism	PPAR
AT406 (SM-406)	Cell death/IAP	E3 Ligase ,IAP
I-BET151 (GSK1210151A)	Epigenetics	Epigenetic Reader Do
Sirtinol	Epigenetics	Sirtuin
Carfilzomib (PR-171)	proteosome inhibitor	Proteasome
IMD 0354	NF-kB	IκB/IKK
Salubrinal	ER stress/ UPR	PERK
JNK-IN-8	MAPK pathway	JNK
Birinapant	IAP inhibitor/cell death	IAP
RG-7112	Apoptosis/Mdm2 inhibitor	Mdm2
AZD1208	JAK/STAT	Pim
UNC1999	Epigenetics	Histone Methyltransferase
Tasisulam	caspase activator/cell death/	Caspase
TH287	DNA damage	MTH1
AZD6738	DNA damage	ATM/ATR
Venetoclax (ABT-199, GDC-0199)	Apoptosis	Bcl-2
Sabutoclax	Apoptosis	Bcl-2, Bcl-xL, Mcl-1, Bfl-1
CB-5083	Transmembrane transporters	ATPase
AZD1390	DNA damage	ATM/ATR
Palbociclib (PD-0332991) HCl	Cell cycle inhibitor	CDK
CX-5461	DNA damage	DNA/RNA Synthesis
NU7026	DNA damage	DNA-PK
Palifosfamide	DNA damage	DNA alkylator
EPZ5676	Epigenetics	Histone Methyltransferase
AZD5991	Cell death- Mcl-1 inhibitor	Mcl-1
AZD4573	Cell cycle	CDK9 inhibitor
Durvalumab	Immunology & Inflammation	PD-1/PDL-1 interaction
ONC206	ER stress/ UPR	DRD2 anatagonist, ER stress inducer
iz-TRAIL	Cell death	death receptor agonist

B.

Drug - concentration (+1μM AZ'1569)	CI value SW837	CI value SNU1411
ONC206 - 1μM	1.2	21.99
Degrasyn - 2μM	1.01	0.94
RG-7112 - 1μM	3.06	1.97
CX-5461- 0.2μM	0.69	1.53
AT-406 - 1μM	1.37*	0.90
IMD0354 - 0.5μM	0.93	0.54
AZD4573 - 1μM	1.00	1.44
CB-5083 - 1μM	1.12	1.14
UNC1999 - 3μM	0.78	1.31
JNK-IN-8 - 1μM	0.56	1.44
Entinostat - 1μM	0.266	0.46
ABT-737 - 1μM	0.57	0.53

C.

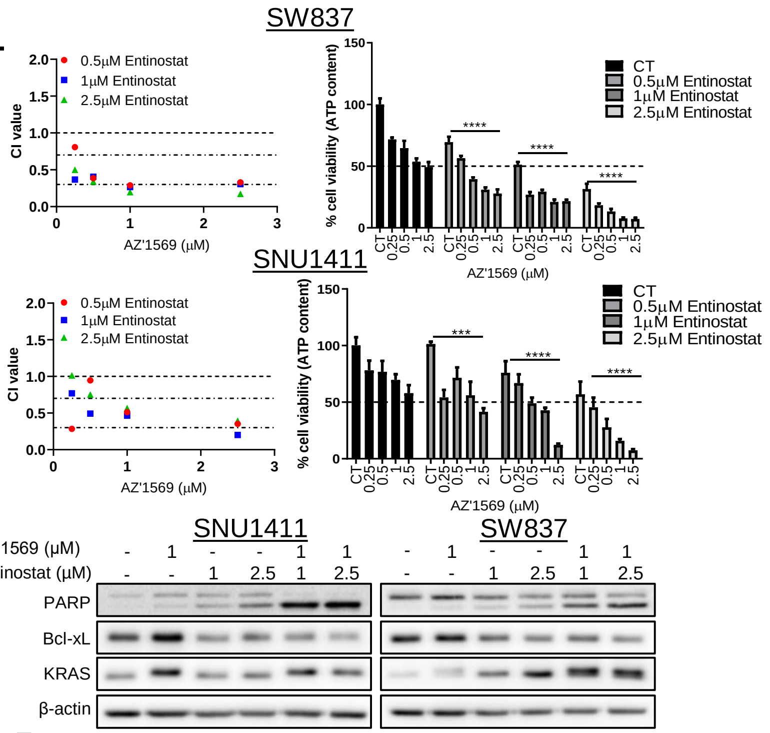

D.

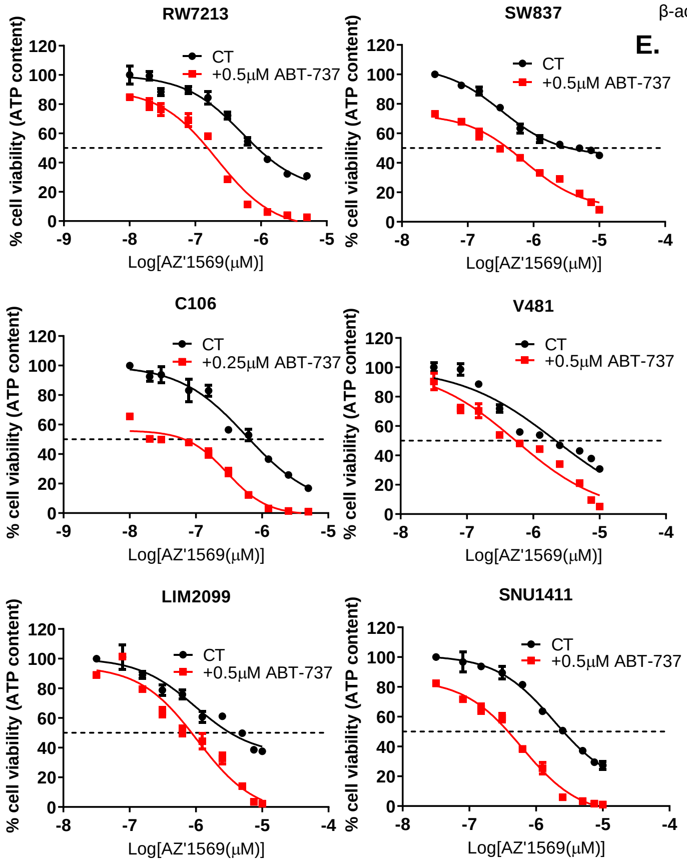

E.

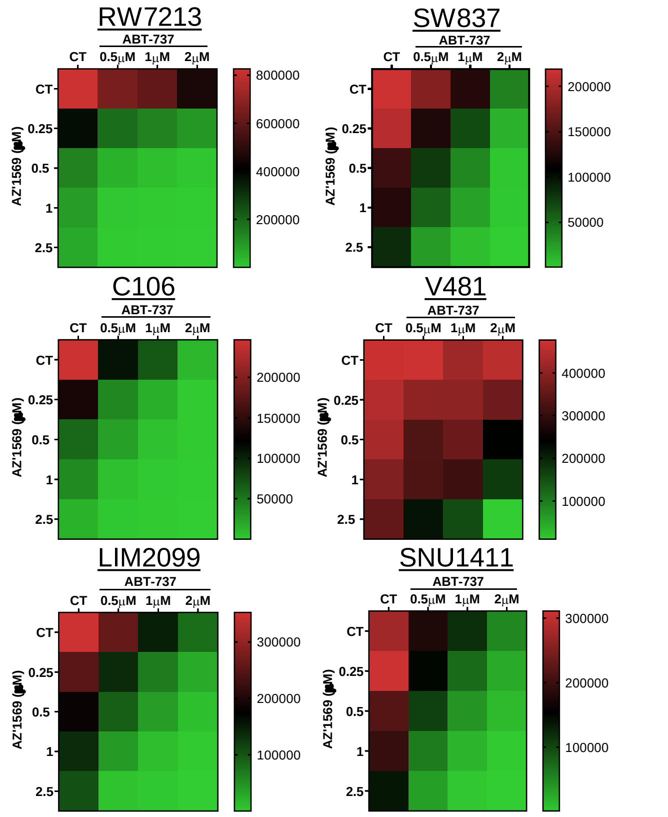

F.

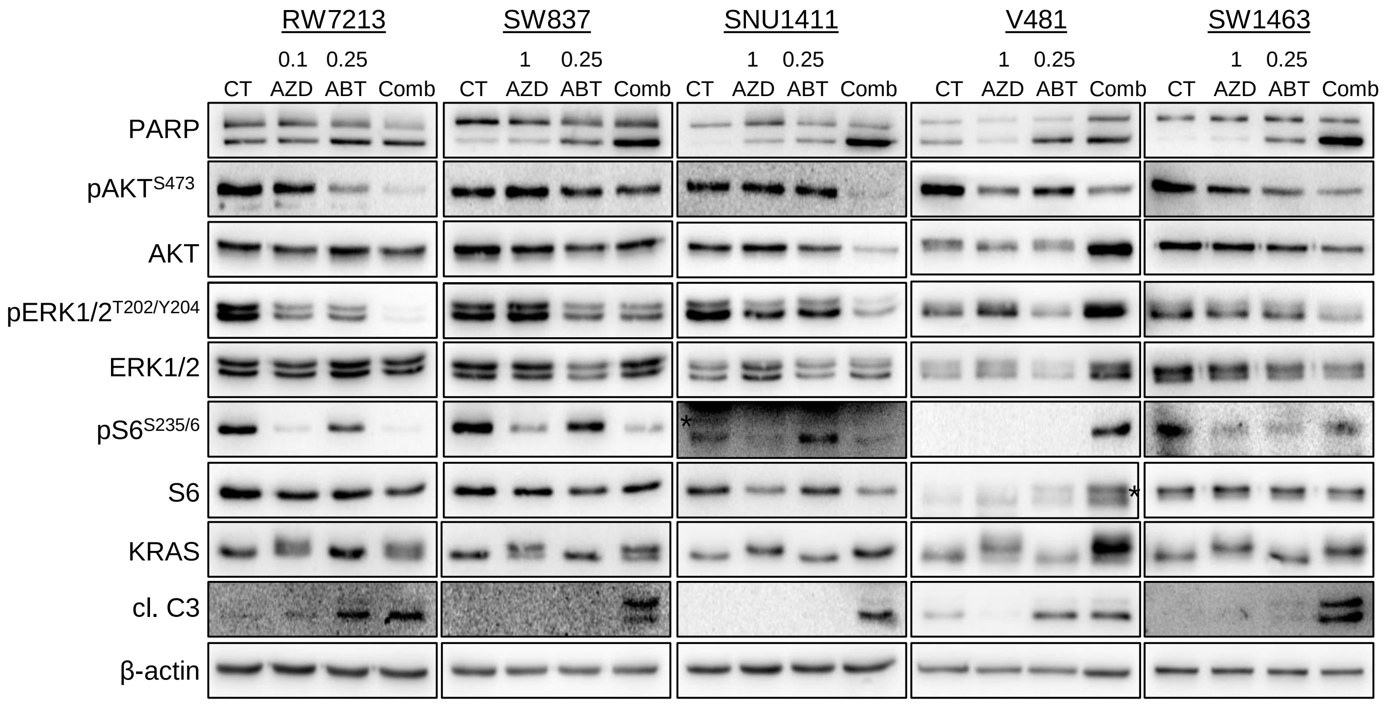

G.

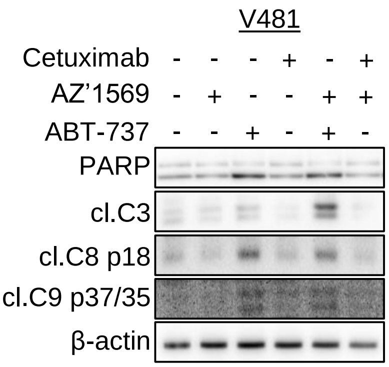

Supplementary figure 4. High-throughput drug screening in *KRAS*^{G12C}MT CRC cells. A. Top: Pie chart of pathways targeted by drugs included in the compound screen. **Bottom:** Table with 45 drugs in the primary drug screen, and pathways/targets modulated by these compounds. **B**. Positive hits from primary screen. SNU1411 and SW837 cells were treated with AZ'1569 alone or combined with each of 12 compounds for 72h and CI/RI values determined. CI/RI values for one concentration of the 12 drugs with 1µM AZ'1569 is shown. * = RI value. **C. Upper/middle**: SW837 and SNU1411 were co-treated with Entinostat (drug 11) and AZ'1569 for 72h and CI values calculated. Absolute cell viability for different combinations is also shown. Dashed line indicates 50% cell viability. **Lower**: PARP, Bcl-xL and KRAS in CRC cells co-treated with Entinostat and AZ'1569. **D**. Dose response curves for AZ'1569 in absence and presence of ABT-737 (drug 12) for 72h. Cell viability was assessed using a CTG assay. Dashed lines represent 50% cell viability. **E**. CTG assays in CRC cells, co-treated with ABT-737 and AZ'1569 for 72h. Heatmaps show absolute cell viability. **F.** *KRAS*^{G12C} cell lines were treated with either AZ'1569, ABT-737, or combination. RW7213 cells were treated with 0.1µM AZ'1569 and SW837, SW1463, SNU1411 and V481 cells were treated with 1µM AZ'1569. All cell lines were co-treated with 0.25µM ABT-737. Cells were collected 24h (RW7213, SW837, SW1463 and SNU1411) and 48h post-treatment (V481cells). WB was used to evaluate the protein expression of PARP, phosphorylated and total AKT, ERK1/2, S6 ribosomal protein, KRAS, cleaved caspase-3 (cl. C3) and β-actin. **G.** PARP, Cleaved-C3/C8/C9 in V481 cells treated with AZ'1569/cetuximab or AZ'1569/ABT-737. Data is representative of three independent experiments.

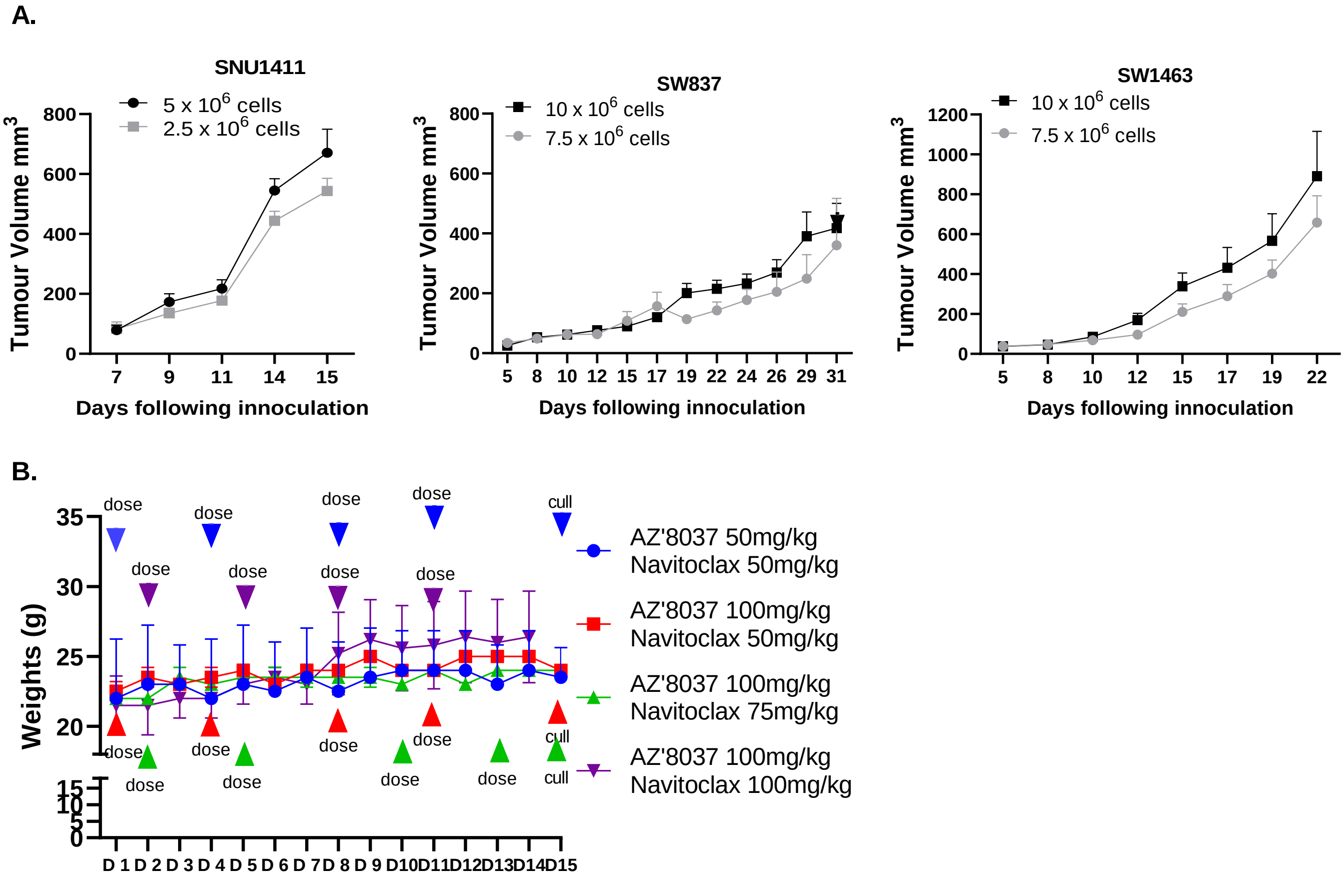

Supplementary figure 5. Treatment of *KRAS*^{G12C}MT CRC mouse models with AZ'8037, Navitoclax or combination. A. Tumour growth curves in NOD-SCID mice for *KRAS*^{G12C}MT SW837, SNU1411 and SW1463 CRC xenografts following inoculation with the indicated cell numbers. **B**. Weights of non-tumour-bearing NOD-SCID mice following 2 weeks treatment with AZ'8037/Navitoclax. Dose levels and days of treatments are indicated on the graphs.

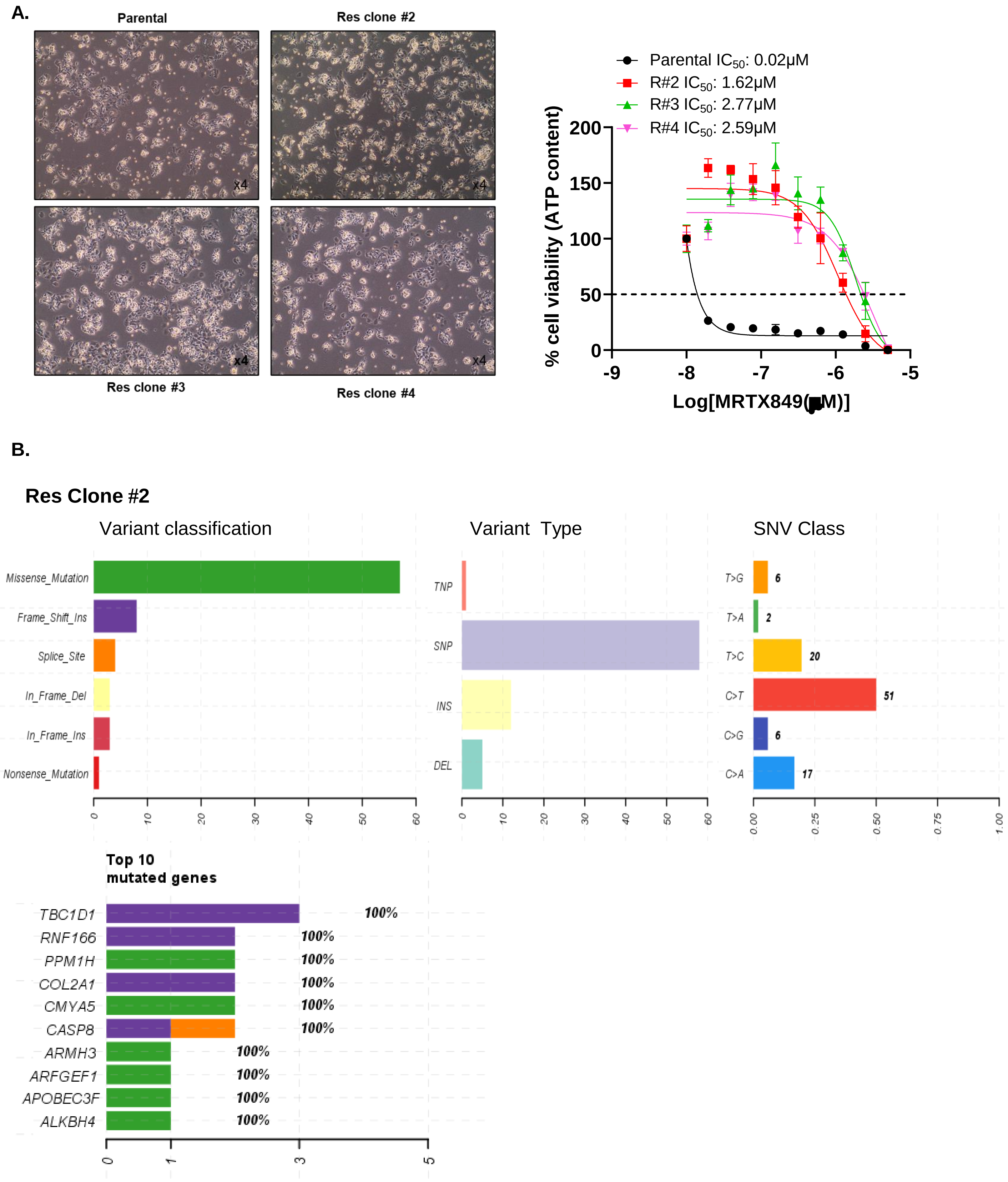
A.
Parental
Res clone #2
Res clone #3
Res clone #4
x4
Parental IC50: 0.02μM
R#2 IC50: 1.62μM
R#3 IC50: 2.77μM
R#4 IC50: 2.59μM
% cell viability (ATP content)
0
50
100
150
200
-9
-8
-7
-6
-5
Log[MRTX849(μM)]
B.
Res Clone #2
Variant classification
Missense_Mutation
Frame_Shift_Ins
Splice_Site
In_Frame_Del
In_Frame_Ins
Nonsense_Mutation
Variant Type
TNP
SNP
INS
DEL
SNV Class
T>G 6
T>A 2
T>C 20
C>T 51
C>G 6
C>A 17
0 10 20 30 40 50 60
0.00 0.25 0.50 0.75 1.00
Top 10
mutated genes
TBC1D1 100%
RNF166 100%
PPM1H 100%
COL2A1 100%
CMYA5 100%
CASP8 100%
ARMH3 100%
ARFGEF1 100%
APOBEC3F 100%
ALKBH4 100%
0 1 3 5

B. continued

Res Clone #3

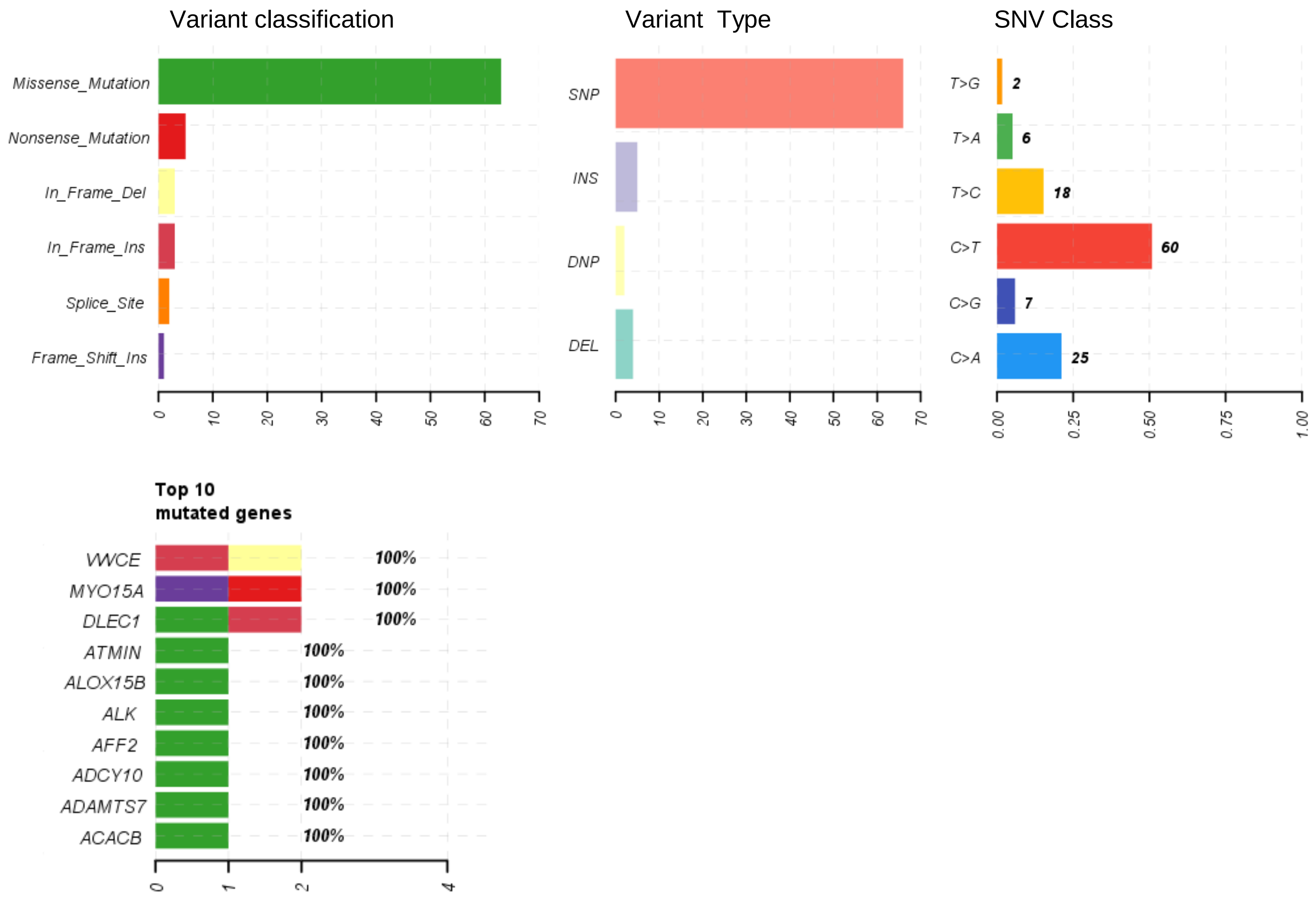

Res Clone #4

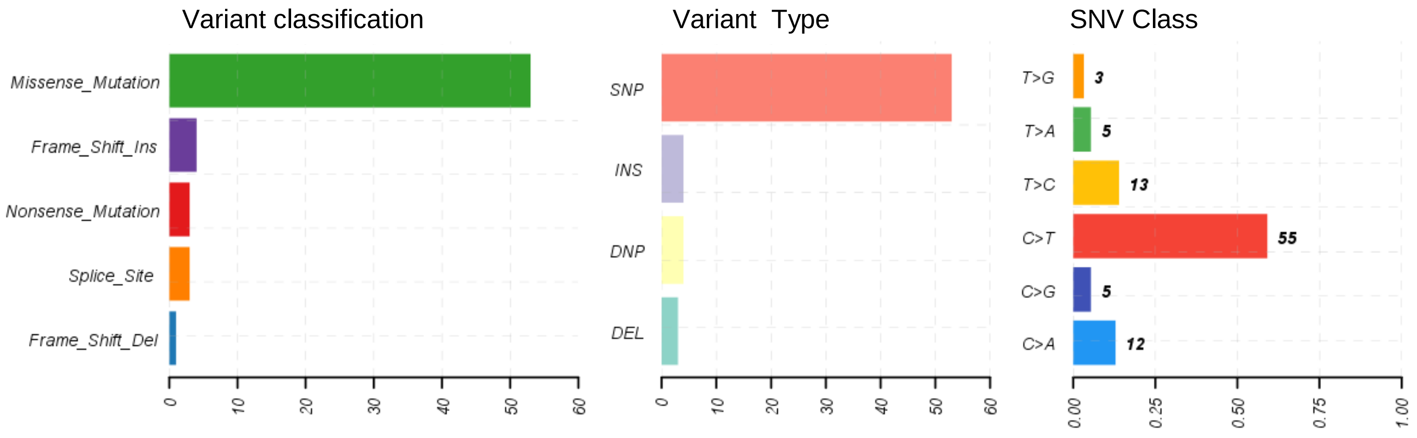

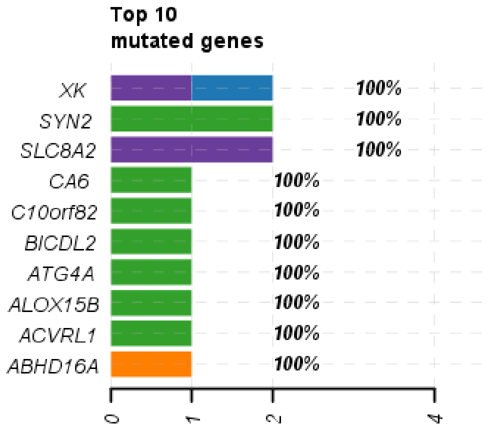

C.

KRAS^{G12C}MT CRC RW7213	Additional basal gene alterations	Acquired mutations
Parental	*TP53* p.L257R, *BRCA2* amplification	None
Res clone #2	*TP53* p.L257R, *BRCA2* amplification	*KRAS* amplification
Res clone #3	*TP53* p.L257R, *BRCA2* amplification	*KRAS* amplification, *EGFR* amplification
Res clone #4	*TP53* p.L257R, *BRCA2* amplification	*KRAS* amplification

D.

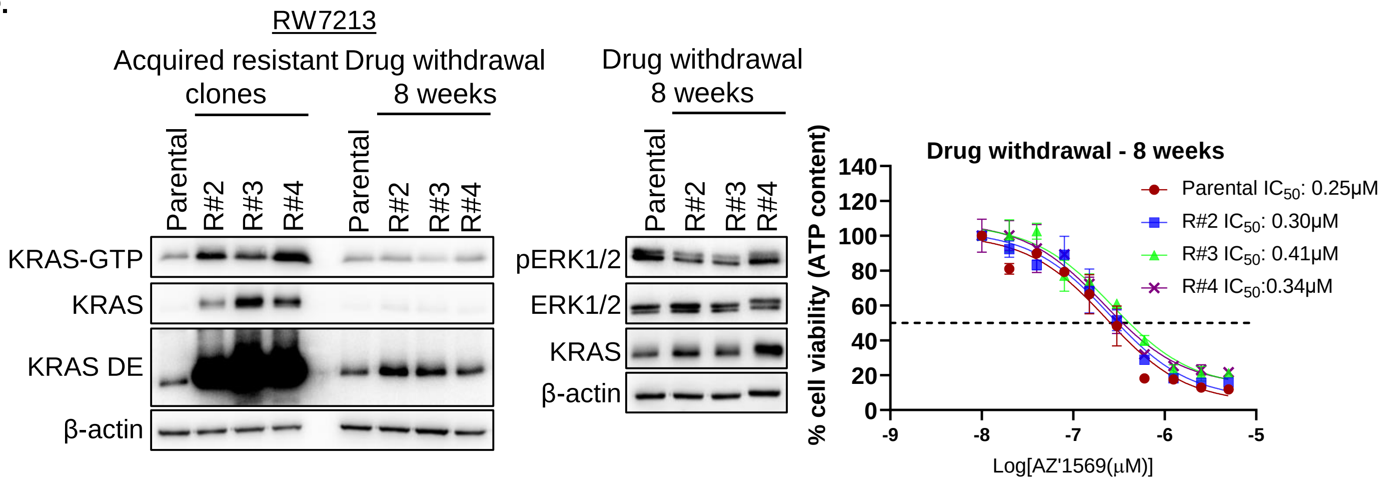
RW7213
Acquired resistant clones
Drug withdrawal 8 weeks
Drug withdrawal 8 weeks
Parental
R#2
R#3
R#4
KRAS-GTP
KRAS
KRAS DE
β-actin
pERK1/2
ERK1/2
KRAS
β-actin
Drug withdrawal - 8 weeks
Parental IC50: 0.25μM
R#2 IC50: 0.30μM
R#3 IC50: 0.41μM
R#4 IC50:0.34μM
% cell viability (ATP content)
Log[AZ'1569(μM)]

E.

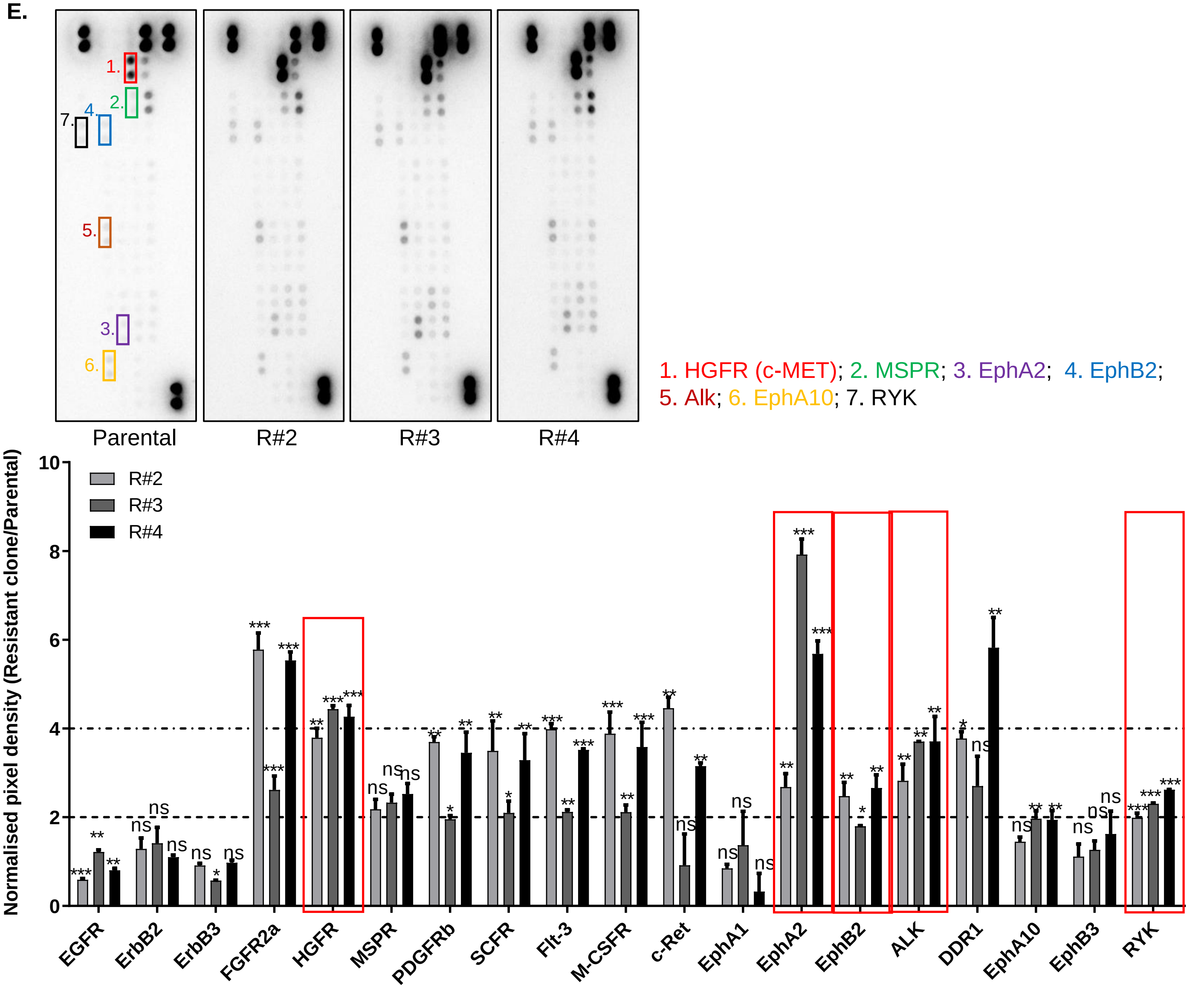
Parental
R#2
R#3
R#4
1. HGFR (c-MET); 2. MSPR; 3. EphA2; 4. EphB2;
5. Alk; 6. EphA10; 7. RYK
Normalised pixel density (Resistant clone/Parental)
R#2
R#3
R#4
EGFR
ErbB2
ErbB3
FGFR2a
HGFR
MSPR
PDGFRb
SCFR
Flt-3
M-CSFR
c-Ret
EphA1
EphA2
EphB2
ALK
DDR1
EphA10
EphB3
RYK

F.

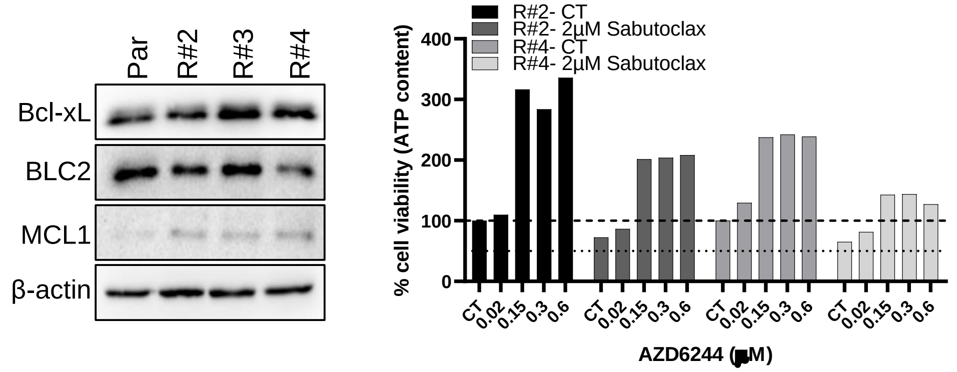
Par
R#2
R#3
R#4
Bcl-xL
BLC2
MCL1
β-actin
R#2- CT
R#2- 2μM Sabutoclax
R#4- CT
R#4- 2μM Sabutoclax
% cell viability (ATP content)
AZD6244 (μM)

G.

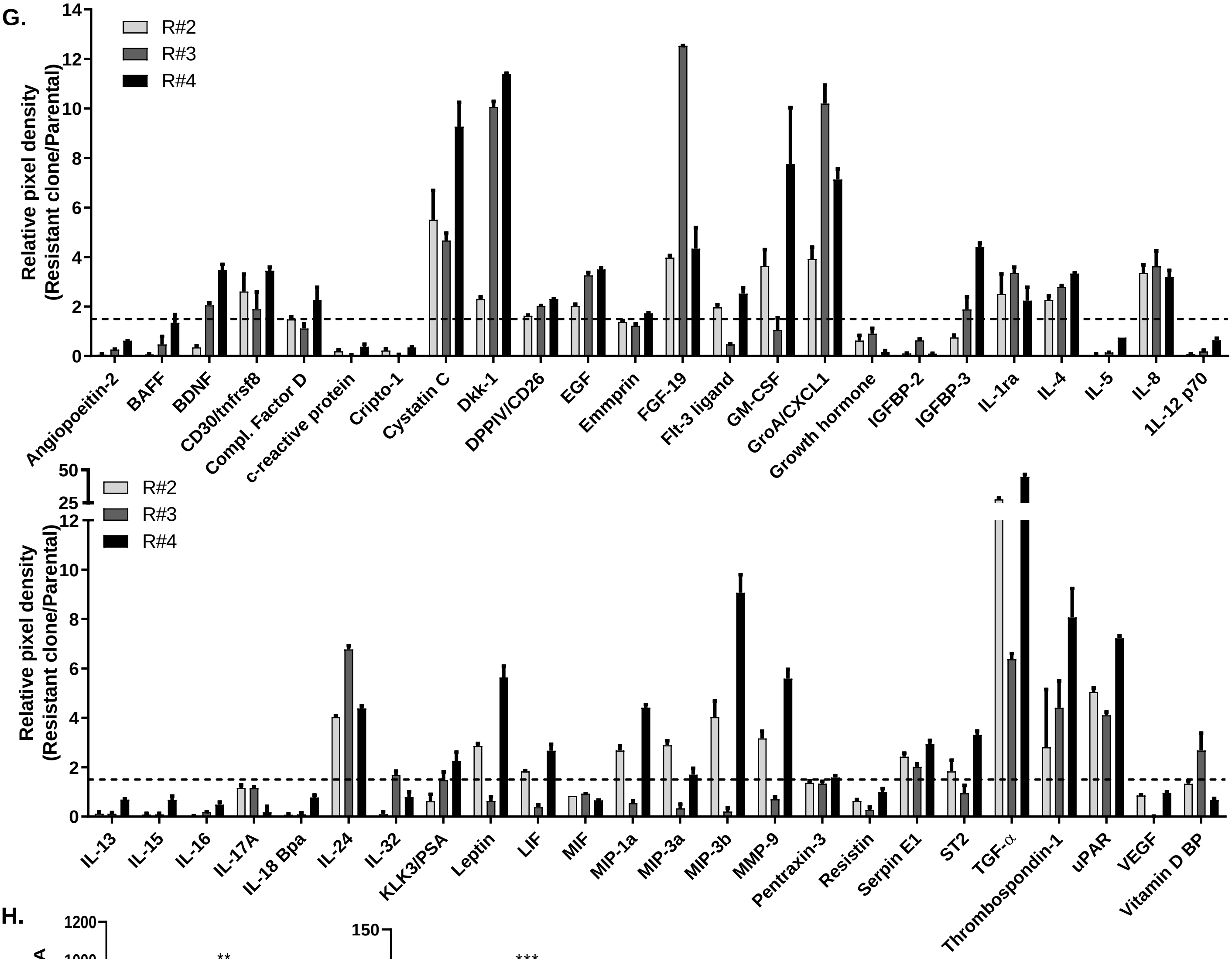

H.

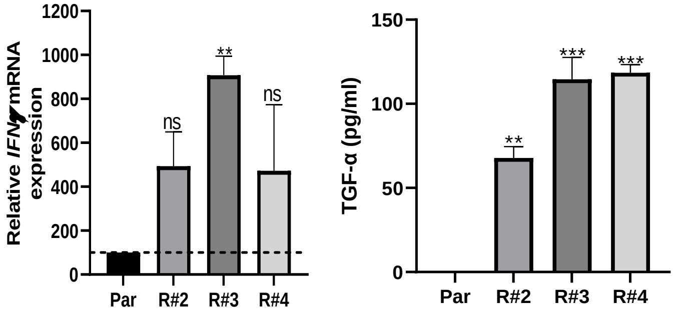

Supplementary figure 6. Characterization of AZ'1569-R RW7213 cells. A. Left: Images of morphology of parental and AZ'1569-resistant RW7213 derivatives (#2, #3, #4), obtained using an EVOS microscope (x4 magnification). **Right:** Parental and AZ'1569-R derivatives were treated with MRTX849 for 72h and cell viability determined using CTG assay. IC_{50} values were calculated using Prism software package. **B**. Medexome sequencing of RW7213 parental and AZ'1569-resistant RW7213 clones. Analysis was performed using maftools package in R. Variant classification, type SNV class and top 10 acquired mutated genes for each of the clones are shown. **C**. Results of NGS sequencing of RW7213 Par and AZ'1569-R clones are shown. **D**. **Left panel:** Basal KRAS-GTP and total KRAS expression in RW7213 parental cells, AZ'1569-resistant derivatives and AZ'1569-resistant RW7213 derivatives following removal of AZ'1569 for 8 weeks. **Middle:** pERK1/2, ERK1/2 and KRAS levels in RW7213 parental cells and AZ'1569-resistant derivatives following removal of AZ'1569 for 8 weeks. **Right**: Dose response curve for AZ'1569 in RW7213 parental cells and AZ'1569-R derivatives following removal of AZ'1569 for 8 weeks. **E.** Human phospho-receptor tyrosine kinase array in RW7213 parental and AZ'1569-R clones. The cell extracts were incubated with membranes containing antibodies to 49 different receptor tyrosine kinases. The membranes were washed and incubated with a cocktail of biotinylated detection antibodies to measure the levels of active kinases. Densitometry was performed on the array panels using ImageJ software. **F. Left:** Basal expression levels of Bcl-xL, BCL2 and MCL-1 in RW7213 parental and AZ'1569-resistant clones. **Right:** AZ'1569-resistant RW7213 clone 2 and clone 4 were treated with increasing concentrations of MEK1/2 inhibitor AZD6244 alone or combined with Sabutoclax for 72h and cell viability determined using CTG assay. **G.** Human cytokine array using conditioned medium of RW7213 parental and AZ'1569-resistant clones. Densitometry was performed on the array panels using ImageJ software. **H. Left:** *IFNγ* mRNA was quantified using RT-PCR. Raw values were normalised to *ACTB* and *GAPDH* expression and were analysed using the ΔΔCT method. A one-way ANOVA was used to calculate statistical significance. **Right.** TGF-α ELISA using conditioned medium from cells. Data is representative of three independent experimental repeats.
